# Systematics of treefrogs of the *Hypsiboas calcaratus* and *Hypsiboas fasciatus* species complex (Anura, Hylidae) with the description of four new species

**DOI:** 10.3897/zookeys.370.6291

**Published:** 2014-01-15

**Authors:** Marcel A. Caminer, Santiago R. Ron

**Affiliations:** 1 Museo de Zoología, Escuela de Biología, Pontificia Universidad Católica del Ecuador, Av. 12 de Octubre y Roca, Aptdo. 17-01-2184, Quito, Ecuador; 2 Current address: Museo Nacional de Ciencias Naturales, Departamento de Biologia Evolutiva y Biodiversidad, José Gutierrez Abascal 2, Madrid 28006, Spain

**Keywords:** Advertisement call, cryptic diversity, conservation status, morphology, new species

## Abstract

We review the systematics of the *Hypsiboas calcaratus* species complex, a group of widely distributed Amazonian hylid frogs. A comprehensive analysis of genetic, morphological, and bioacoustic datasets uncovered the existence of eleven candidate species, six of which are confirmed. Two of them correspond to *Hypsiboas fasciatus* and *Hypsiboas calcaratus* and the remaining four are new species that we describe here. *Hypsiboas fasciatus* *sensu stricto* has a geographic range restricted to the eastern Andean foothills of southern Ecuador while *Hypsiboas calcaratus* *sensu stricto* has a wide distribution in the Amazon basin. *Hypsiboas almendarizae* **sp. n.** occurs at elevations between 500 and 1950 m in central and northern Ecuador; the other new species (*H. maculateralis* **sp. n.**, *H. alfaroi* **sp. n.**, and *H. tetete* **sp. n.**) occur at elevations below 500 m in Amazonian Ecuador and Peru. The new species differ from *H. calcaratus* and *H. fasciatus* in morphology, advertisement calls, and mitochondrial and nuclear DNA sequences. Five candidate species from the Guianan region, Peru, and Bolivia are left as unconfirmed. Examination of the type material of *Hyla steinbachi*, from Bolivia, shows that it is not conspecific with *H. fasciatus* and thus is removed from its synonymy.

## Abstract (es)

Revisamos la sistemática del complejo de especies *Hypsiboas calcaratus*, un grupo de ranas hílidas de amplia distribución en la Amazonía. Un análisis exhaustivo de datos genéticos, morfológicos y bioacústicos revelaron la existencia de once especies candidatas, de las cuales seis son confirmadas. Dos de ellas corresponden a *Hypsiboas fasciatus* e *Hypsiboas calcaratus* y las cuatro restantes son especies nuevas que describimos aquí. *Hypsiboas fasciatus* *sensu stricto* tiene un rango geográfico restringido a las estribaciones orientales de los Andes del sur del Ecuador, mientras que *Hypsiboas calcaratus* *sensu stricto* tiene una distribución amplia en la cuenca amazónica. *Hypsiboas almendarizae* **sp. n.** se encuentra a alturas entre los 500 y 1950 m en el centro y el norte del Ecuador, mientras que las otras nuevas especies (*H. maculateralis* **sp. n.**, *H. alfaroi* **sp. n.** y *H. tetete* **sp. n.**) se encuentran a elevaciones bajo 500 m en la Amazonía de Ecuador y Perú. Las nuevas especies difieren de *H. calcaratus* e *H. fasciatus* en morfología, cantos de anuncio y secuencias de ADN mitocondrial y nuclear. Cinco especies candidatas de la región de las Guayanas, Perú y Bolivia quedan como no confirmadas. La examinación del material tipo de *Hyla steinbachi* indica que no es conespecífica con *H. fasciatus* y por lo tanto se remueve de su sinonimia.

## Introduction

Management and conservation planning of biodiversity requires proper species identifications and comprehensive inventories. Recent DNA-based studies of Amazonian amphibians have shown the existence of a large proportion of undescribed species that have escaped detection in morphology-based assessments (Elmer et al. 2013; Elmer and Cannatella 2008; Fouquet et al. 2012; Fouquet et al. 2007; Funk et al. 2012; Jungfer et al. 2013; Ron et al. 2012). The discovery of these taxonomic voids highlights the need of renewed and intensive efforts to discover and catalogue amphibians in the Amazon region. This is particularly urgent because recent evaluations of the potential impact of climate change have shown that Amazonian amphibians are highly vulnerable (Foden et al. 2013). Conservation measures could be ineffective without reliable baseline data on species identity and distribution.

Among the species complexes that have been proven to contain a large proportion of hidden species richness is the *Hypsiboas calcaratus*-*Hypsiboas fasciatus* species complex (Funk et al. 2012). Both species are widely distributed in the Amazon basin (Azevedo-Ramos et al. 2010; Icochea et al. 2004) and are characterized by having brown dorsal coloration, basal hand webbing, and presence of dark marks or bars on the flanks and concealed surfaces of the thighs (Duellman 1973). According to most current accounts *Hypsiboas calcaratus* is a species with large calcars and vertical bars on the flanks and thighs while *Hypsiboas fasciatus* has small calcars and irregular black spots on the flanks and thighs (e.g., De la Riva et al. 2000; Duellman 2005; Rodríguez and Duellman 1994). Presumably, these accounts are based on the taxonomic review by Duellman (1973), who included them in the *Hyla geographica* group. In their phylogeny of Hylidae, Faivovich et al. (2005) demonstrated that the “*Hyla geographica*” group was paraphyletic. *Hypsiboas calcaratus* and *Hypsiboas fasciatus* were assigned to the *Hypsiboas albopunctatus* group, which was diagnosed by genetic characters. The *Hypsiboas albopunctatus* group, according to the definition of Faivovich et al. (2005), contains nine formally described species: *Hypsiboas albopunctatus* (Spix, 1824), *Hypsiboas calcaratus* (Troschel, 1848), *Hypsiboas dentei* (Bokermann, 1967), *Hypsiboas fasciatus* (Günther, 1858), *Hypsiboas heilprini* (Noble, 1923), *Hypsiboas lanciformis* (Cope, 1871), *Hypsiboas leucocheilus* (Caramaschi & Niemeyer, 2003), *Hypsiboas multifasciatus* (Günther, 1859) and *Hypsiboas raniceps* (Cope, 1862). Wiens et al. (2010) found strong support for the *Hypsiboas albopunctatus* group with *Hypsiboas calcaratus* and *Hypsiboas fasciatus* being sister to each other. Pyron and Wiens (2011), however, show *Hypsiboas fasciatus* as sister of *Hypsiboas dentei* and *Hypsiboas calcaratus* as sister of a clade formed by *Hypsiboas lanciformis*, *Hypsiboas multifasciatus* and *Hypsiboas albopunctatus*.

Recently published genetic, morphological and bioacoustic evidence suggests that *Hypsiboas fasciatus* and *Hypsiboas calcaratus* are a species complex. A phylogenetic analysis based on mitochondrial DNA (mtDNA) sequences revealed the presence of unconfirmed candidate species (Fouquet et al. 2007a). More recently, Funk et al. (2012) analyzed variation in mitochondrial and nuclear DNA, acoustic and morphological characters of populations of both species and found the existence of nine candidate species. Here in we incorporate additional genetic and morphological data and identify 11 candidate species of which six are confirmed candidate species. We describe four of them including their advertisement calls and variation in external morphology.

## Methods

Morphological terminology follows Duellman (1970 and 1973). Examined specimens (listed in Appendix) are housed in Museo de Zoología de la Pontificia Universidad Católica del Ecuador (QCAZ), Museo de Historia Natural Gustavo Orcés at Escuela Politécnica Nacional (EPN), National Museum of Natural History (USNM), and Natural History Museum (BMNH). We also examined the type material of *Hyla leptoscelis* (holotype BMNH 1947.2.23.10), *Hypsiboas fasciatus* (holotype BMNH 58.4.25.22) and *Hyla steinbachi* (syntypes BMNH 1947.2.13.61–63) deposited at the British Museum of Natural History.

Adult specimens were measured for the following variables: (1) snout-vent length; (2) head length; (3) head width; (4) femur length; (5) tibia length; (6) foot length; (7) tympanum diameter; and (8) calcar length. Measurements were made according to the methodology described in Duellman (1970) with digital calipers (nearest 0.01 mm) from specimens fixed in 10% formalin and preserved in 70% ethanol. Sex was determined by gonadal inspection or by the presence of prepollical spines and/or vocal sac folds in males.

Multivariate analyses of variance (MANOVA) and Principal Components Analysis (PCA) were used to assess the degree of morphometric differentiation among adult individuals of all species considered. To remove the effect of covariation in size, the MANOVA and PCA were applied to the residuals from the linear regressions between six morphometric variables and SVL; this procedure was performed separately for males (*n* = 136) and females (*n* = 34). For the PCA, only components with eigenvalues > 1 were retained. Finally, variables were compared between species with Student’s *t*-tests after ensuring their fit to a normal distribution.

Multivariate morphometric analyses were based in 170 adult specimens (populations of origin in parentheses): 25 *Hypsiboas fasciatus* (including the holotype; Centro Shuar Yawi, La Pradera, Las Orquídias, Limón, Romerillos Alto, Miazi Alto, Tink and Zamora), 27 *Hypsiboas almendarizae* sp. n. (Baños, El Rosario, Guamote, Limón Indanza and Río Hollín), 39 *Hypsiboas calcaratus* (Añangu, Canelos, Chiroisla, Comunidad Santa Rosa, Edén, El Coca, Kapawi, Estación Científica Yasuní PUCE, La Primavera, Puerto Bolivar, San Vicente, Selva Lodge and Tena), 28 *Hypsiboas maculateralis* sp. n. (Bataburo Lodge, Chiroisla, Comunidad Santa Rosa, Cuyabeno, Edén, El Coca, Huiririma, La Primavera, Selva Lodge, San Vicente, Santa Teresita and Zábalo), 44 *Hypsiboas alfaroi* sp. n. (Añangu, Chiroisla, Cuyabeno, Edén, El Coca, Estación Científica Yasuní PUCE, Huiririma, La Primavera, Lago Agrio, Nuevo Rocafuerte, Pañacocha, Puerto Bolivar, San Vicente and Selva Lodge), and 7 *Hypsiboas tetete* sp. n. (Comunidad Santa Rosa and Jatun Sacha). See Tables 1 and 2 and Appendix for detailed locality information. Statistical analyses were carried out in SPSS ® (2009, version 17.0 for Windows).

**Table 1.** Descriptive statistics for morphometric measurements of male *Hypsiboas alfaroi*, *Hypsiboas almendarizae*, *Hypsiboas calcaratus*, *Hypsiboas fasciatus*, *Hypsiboas maculateralis*, and *Hypsiboas tetete* used for Principal Component Analysis. Mean ± SD is given with range below. Bold figures represent combined values for males of all populations. Abbreviations are: SVL = Snout-vent length; FOOT = Foot length; HL = Head length; HW = Head width; ED = Eye diameter; TD = Tympanum diameter; TL = Tibia length; FL = Femur length; CL = Calcar length. All measurements are in mm.

Species	SVL	FOOT	HL	HW	ED	TD	TL	FL	CL
***Hypsiboas alfaroi*** **(*n* = 32)**	**32.80 ± 1.97** **27.91–36.27**	**12.54 ± 0.65** **11.33–13.80**	**8.95 ± 0.98** **7.13–11.84**	**10.07 ± 0.61** **8.72–10.95**	**3.25 ± 0.36** **2.52–4.03**	**2.28 ± 0.28** **1.70–3.01**	**18.50 ± 0.95** **16.54–20.50**	**16.25 ± 0.96** **14.71–17.91**	**Calcar absent**
Yasuní PUCE (*n* = 7)	32.20 ± 1.98 27.91–35.19	12.73 ± 0.61 11.33–13.46	8.35 ± 0.60 7.40–8.99	10.10 ± 0.53 8.79–10.80	3.29 ± 0.36 2.90–4.03	2.24 ± 0.11 2.08–2.50	18.66 ± 1.10 16.54–20.50	16.25 ± 0.98 14.81–17.89	Calcar absent
Huiririma (*n* = 2)	33.61–34.22	12.95–13.80	9.69–9.75	10.37–10.77	3.25–3.32	2.22–2.51	18.10–19.32	15.50–17.17	Calcar absent
Nuevo Rocafuerte (*n* = 5)	33.99 ± 2.13 30.34–35.88	12.51 ± 0.81 11.41–13.71	9.91 ± 1.16 8.98–11.84	10.23 ± 0.63 9.26–10.95	3.11 ± 0.41 2.52–3.61	2.05 ± 0.23 1.70–2.28	18.82 ± 1.23 16.95–20.05	16.96 ± 1.22 14.92–17.91	Calcar absent
Pañacocha (*n* = 3)	33.04 ± 0.52 32.64–33.63	12.10 ± 0.19 11.95–12.31	9.58 ± 0.10 9.46–9.64	9.65 ± 0.68 8.87–10.14	3.34 ± 0.33 2.97–3.62	2.29 ± 0.05 2.25–2.34	17.97 ± 0.29 17.66–18.22	16.27 ± 0.20 16.04–16.41	Calcar absent
Selva Lodge (*n* = 2)	32.11–33.85	12.72–13.14	8.58–9.06	9.18–10.53	2.73–3.61	2.11–2.91	18.47–18.90	14.71–16.21	Calcar absent
***Hypsiboas almendarizae*** **(*n* = 23)**	**37.64 ± 2.01** **34.31–44.56**	**15.04 ± 0.82** **12.99–16.00**	**9.74 ± 0.86** **8.33–11.35**	**11.8 1 ± 0.60** **10.80–13.07**	**4.02 ± 0.34** **3.36–4.56**	**2.27 ± 0.24** **1.63–2.74**	**21.27 ± 0.94** **19.53–23.04**	**19.24 ± 1.06** **15.84–21.52**	**1.51 ± 0.24** **1.09–1.88**
Baños (*n* = 2)	37.39–37.98	13.43–15.16	9.51–9.69	11.88–12.00	4.22–4.32	2.31–2.30	19.53–21.21	18.48–19.38	1.32–1-39
El Rosario (*n* = 3)	38.90 ± 1.79 37.56–40.92	15.29 ± 0.40 14.89–15.70	11.06 ± 0.46 10.52–11.34	12.40 ± 0.27 12.15–12.68	4.42 ± 0.12 4.35–4.56	2.30 ± 0.20 2.13–2.53	22.45 ± 0.66 21.74–22.58	19.37 ± 0.23 19.10–19.50	1.64 ± 0.07 1.56–1.68
Limón Indanza (*n* = 12)	36.72 ± 1.23 34.31–38.60	14.97 ± 0.88 12.99–16.00	9.19 ± 0.39 8.33–9.74	11.73 ± 0.56 10.80–12.68	3.94 ± 0.34 3.36–4.54	2.25 ± 0.23 2.02–2.74	21.01 ± 0.73 19.64–22.24	19.13 ± 1.19 15.84–20.23	1.51 ± 0.28 1.09–1.88
Río Hollín (*n* = 2)	38.46–44.56	15.73–15.75	10.20–11.35	11.72–13.07	3.60–4.38	1.63–2.25	21.80–22.62	18.83–21.52	1.33–1.57
***Hypsiboas calcaratus*** **(*n* = 35)**	**36.82 ± 2.59** **27.61–42.50**	**15.00 ± 1.31** **10.68–17.44**	**10.14 ± 1.06** **7.71–12.63**	**11.93 ± 1.01** **9.16–13.72**	**3.63 ± 0.37** **2.47–4.52**	**2.48 ± 0.21** **1.92–2.94**	**22.10 ± 1.57** **16.76–24.61**	**19.03 ± 1.48** **13.82–21.44**	**2.03 ± 0.33** **1.32–2.65**
Canelos (*n* = 3)	36.59 ± 1.49 34.89–37.69	14.64 ± 0.59 14.08–15.26	10.48 ± 0.55 10.05–11.10	11.68 ± 0.59 11.01–12.13	3.87 ± 0.30 3.69–4.22	2.43 ± 0.24 2.18–2.66	21.78 ± 0.34 21.39–22.03	18.28 ± 0.53 17.67–18.66	2.06 ± 0.25 1.89–2.34
Tena (*n* = 5)	35.23 ± 2.80 32.10–39.15	13.87 ± 1.10 12.65–15.20	9.12 ± 1.15 8.18–10.69	11.95 ± 1.24 10.69–13.72	3.71 ± 0.31 3.33–4.04	2.57 ± 0.23 2.39–2.94	20.69 ± 1.29 18.77–21.98	18.28 ± 1.13 16.51–19.43	2.05 ± 0.39 1.56–2.54
Edén (*n* = 2)	36.88–37.09	14.39–14.57	9.75–10.72	11.30–12.24	3.29–3.55	2.41–2.71	20.70–21.85	17.42–17.63	1.84–2.13
El Coca (*n* = 2)	34.83–34.96	14.21–14.42	9.05–9.16	11.19–11.31	2.47–2.94	2.10–2.37	20.04–22.52	16.46–19.22	1.68–1.98
Estación Científica Yasuní PUCE (*n* = 18)	37.78 ± 1.53 35.21–40.10	15.73 ± 0.89 14.54–17.44	10.43 ± 0.89 8.99–12.63	12.20 ± 0.90 9.59–13.33	3.71 ± 0.30 3.36–4.52	2.48 ± 0.17 2.23–2.87	22.99 ± 0.98 21.12–24.61	19.91 ± 0.85 18.40–21.44	2.06 ± 0.36 1.32–2.47
Parque Nacional Yasuní, Pompeya (*n* = 2)	36.11–36.31	13.50–15.52	10.42–11.43	11.02–13.13	3.60–4.12	2.43–2.63	21.14–23.47	19.38–19.55	1.90–2.04
***Hypsiboas fasciatus*** **(*n* = 19)**	**35.40 ± 1.65** **32.65–37.74**	**14.00 ± 0.59** **12.85–15.24**	**9.651 ± 0.66** **8.42–11.53**	**11.39 ± 0.59** **10.41–12.33**	**3.71 ± 0.34** **3.15–4.42**	**2.42 ± 0.25** **2.03–2.78**	**20.07 ± 0.89** **18.63–21.71**	**18.18 ± 0.87** **16.12–19.94**	**1.35 ± 0.15** **1.08–1.63**
Centro Shuar Yawi (*n* = 4)	35.79 ± 2.14 36.22–37.72	13.81 ± 1.02 12.85–15.24	9.69 ± 0.57 9.03–10.43	11.60 ± 0.71 10.66–12.33	3.91 ± 0.38 3.50–4.42	2.40 ± 0.25 2.20–2.73	20.55 ± 1.34 18.63–21.71	18.14 ± 1.42 16.12–19.42	1.35 ± 0.07 1.25–1.41
La Pradera (*n* = 4)	34.69 ± 0.82 33.74–35.74	14.18 ± 0.14 14.00–14.22	9.61 ± 0.31 9.36–10.06	10.98 ±0.43 10.41–11.44	3.66 ± 0.16 3.56–3.82	2.31 ± 0.33 2.03–2.78	19.27 ± 0.43 19.06–19.83	18.00 ± 0.53 17.30–18.44	1.50 ± 0.10 1.39–1.63
Tiink (*n* = 6)	34.79 ± 1.89 32.65–37.50	13.96 ± 0.52 13.46–14.77	9.72 ± 1.06 8.42–11.53	11.17 ± 0.63 10.60–12.10	3.55 ± 0.42 3.15–4.09	2.50 ± 0.19 2.26–2.72	20.30 ± 0.80 19.25–21.12	18.44 ± 1.00 17.29–19.94	1.23 ± 0.16 1.08–1.47
Zamora (*n* = 2)	34.55–36.95	13.52–13.58	8.94–9.55	11.54–11.85	3.85–3.95	2.51–2.78	19.21–19.61	17.71–18.04	1.25–1.40
***Hypsiboas maculateralis*** **(*n* = 22)**	**36.00 ± 1.92** **31.86–39.17**	**13.55 ± 1.03** **11.10–16.22**	**9.59 ± 1.18** **6.62–11.19**	**11.08 ± 0.70** **9.21–12.29**	**3.55 ± 0.49** **2.63–4.39**	**2.17 ± 0.28** **1.26–2.63**	**20.67 ± 1.48** **16.94–23.23**	**18.18 ± 1.40** **15.31–20.57**	**1.67 ± 0.32** **1.15–2.52**
Bataburo Lodge (*n* = 2)	34.20–38.18	13.02–13.90	9.58–11.10	10.27–11.81	3.36–4.24	2.09–2.14	18.60–22.21	17.77–19.19	1.24–1.47
Edén (*n* = 3)	34.74 ± 2.68 31.86–37.17	12.65 ± 1.37 11.10–13.68	7.93 ± 1.34 6.62–9.30	10.38 ± 1.02 9.21–11.09	3.29 ± 0.63 2.63–3.88	2.12 ± 0.16 1.94–2.24	18.83 ± 1.64 16.94–19.82	16.85± 1.35 15.31–17.84	1.81 ± 0.22 1.56–1.96
Reserva de Producción Faunística Cuyabeno PUCE (*n* = 7)	36.40 ± 1.53 33.71–38.69	13.59 ± 0.60 12.57–14.19	10.23 ± 0.75 9.15–11.19	11.32 ± 0.45 10.69–12.14	3.97 ± 0.26 3.67–4.39	2.29 ± 0.15 2.10–2.57	21.65 ± 0.69 20.63–22.43	19.56 ± 0.83 18.36–20.57	1.89 ± 0.35 1.55–2.52
La Primavera (*n* = 2)	35.26–38.49	13.41–14.58	9.36–11.01	10.90–11.14	3.02–3.67	1.26–2.28	20.84–20.91	17.20–17.69	1.27–1.86
Selva Lodge (*n* = 2)	37.48–39.17	12.98–16.22	8.77–9.94	11.72–12.29	3.24–3.33	2.43–2.63	20.86–23.23	16.12–19.90	1.61–1.90
***Hypsiboas tetete*** **Comunidad Santa Rosa** **(*n* = 5)**	**31.72 ± 0.42** **31.15–32.24**	**12.22 ± 0.15** **12.01–12.40**	**8.13 ± 0.53** **7.48–8.75**	**10.32 ± 0.24** **9.97–10.64**	**3.60 ± 0.25** **3.38–4.02**	**2.62 ± 0.35** **2.25–3.01**	**17.62 ± 0.24** **17.30–17.93**	**16.50 ± 0.36** **16.09–17.00**	**Calcar absent**

**Table 2.** Descriptive statistics for morphometric measurements of female *Hypsiboas alfaroi*, *Hypsiboas almendarizae*, *Hypsiboas calcaratus*, *Hypsiboas fasciatus*, *Hypsiboas maculateralis*, and *Hypsiboas tetete* used for Principal Component Analysis. Mean ± SD is given with range below. Bold figures represent combined values for females from all populations. Abbreviations are: SVL = Snout-vent length; FOOT = Foot length; HL = Head length; HW = Head width; ED = Eye diameter; TD= Tympanum diameter; TL = Tibia length; FL = Femur length; CL = Calcar length. All measurements are in mm.

Species	SVL	FOOT	HL	HW	ED	TD	TL	FL	CL
***Hypsiboas alfaroi*** **(*n* = 12)**	**44.51 ± 3.09** **39.68–49.21**	**16.72 ± 1.96** **11.39–18.94**	**11.59 ± 1.17** **8.90–13.09**	**14.21 ± 1.33** **1.55–15.76**	**4.12 ± 0.56** **3.23–5.00**	**2.96 ± 0.32** **2.22–3.44**	**25.47 ± 1.93** **22.88–9.44**	**22.77 ± 1.64** **19.88–25.66**	**Calcar absent**
Estación Científica Yasuní PUCE (*n* = 4)	42.81 ± 2.08 40.27–45.37	15.82 ± 2.99 11.39–17.71	11.71 ± 0.66 11.03–12.28	13.93 ± 1.05 12.41–14.70	4.12 ± 0.36 3.59–4.34	2.87 ± 0.23 2.60–3.14	24.83 ± 1.52 23.18–26.80	22.91 ± 1.32 21.17–24.35	Calcar absent
Playas de Cuyabeno (*n* = 2)	44.51–48.01	16.60–17.41	12.15–13.09	14.99–15.71	4.91–5.00	3.18–3.44	24.61–25.11	21.16–23.38	Calcar absent
***Hypsiboas almendarizae*** **(*n* = 4)**	**48.11 ± 6.88** **37.80–51.94**	**19.36 ± 2.92** **15.11–21.64**	**11.59 ± 1.96** **8.70–12.99**	**14.77 ± 2.45** **11.15–16.59**	**4.08 ± 0.66** **3.45–4.68**	**2.52 ± 0.41** **2.07–3.00**	**25.81 ± 6.88** **15.51–29.74**	**24.46 ± 3.41** **19.40–26.62**	**1.89 ± 0.55** **1.20–2.53**
Nueve de Octubre (*n* = 3)	51.54 ± 0.35 51.26–51.94	20.78 ± 0.88 19.87–21.64	12.55 ± 0.43 12.14–12.99	15.98 ± 0.54 15.61–16.59	4.28 ± 0.62 3.57–4.68	2.68 ± 0.34 2.32–3.00	29.25 ± 0.44 28.87–29.74	26.15 ± 0.60 25.47–26.62	2.12 ± 0.36 1.87–2.53
***Hypsiboas calcaratus*** **(*n* = 4)**	**50.92 ± 4.80** **45.94–56.29**	**20.56 ± 2.05** **18.44–23.17**	**13.08 ± 2.03** **11.04–15.83**	**16.46 ± 1.85** **14.59–18.42**	**4.14 ± 0.86** **3.57–5.42**	**3.12 ± 0.50** **2.67–3.77**	**31.00 ± 3.13** **28.10–35.29**	**26.05 ± 2.15** **24.47–29.09**	**2.62 ± 0.16** **2.42–2.78**
***Hypsiboas fasciatus*** **(*n* = 5)**	**51.89 ± 3.18** **47.16–54.84**	**20.44 ± 1.23** **18.79–21.98**	**13.91 ± 0.79** **12.59–14.53**	**16.57 ± 0.72** **15.80–17.38**	**4.83 ± 0.43** **4.28–5.32**	**3.25 ± 0.38** **2.70–3.77**	**29.58 ± 1.46** **28.55–32.09**	**27.02 ± 1.34** **25.74–29.20**	**1.95 ± 0.15** **1.73–2.09**
***Hypsiboas maculateralis*** **(*n* = 6)**	**45.18 ± 9.39** **32.04–55.31**	**16.27 ± 3.29** **11.25–19.77**	**11.76 ± 2.40** **8.28–14.10**	**14.21 ± 2.95** **10.01–17.37**	**3.78 ± 0.26** **3.34–4.06**	**2.43 ± 0.52** **1.70–3.00**	**25.23 ± 4.42** **18.04–29.66**	**21.90 ± 4.22** **15.48–26.54**	**1.61 ± 0.40** **1.09–2.22**
Zábalo (*n* = 2)	32.04–38.26	11.25–14.58	8.28–9.28	10.01–12.38	3.34–3.61	1.70–2.03	18.04–22.82	15.48–19.15	1.09–1.39
***Hypsiboas tetete*** **Jatun Sacha** **(*n* = 2)**	**45.33–45.85**	**16.81–18.17**	**11.15–12.96**	**13.66–14.11**	**4.09–4.96**	**3.56–3.85**	**25.45–25.78**	**21.18–21.81**	**Calcar absent**

In the Diagnosis sections, coloration refers to preserved specimens unless otherwise noted. Seven qualitative morphological characters were evaluated: (1) dorsal coloration, (2) ventral coloration, (3) iris coloration, (4) middorsal stripe [i. present, ii. absent], (5) black flecks on the neck and chest [i. present, ii. absent], (6) marks on flank and hidden surfaces of the thighs [i. dark transversal bars, ii. thin dark transversal bars, iii. dark blotches, iv. dark dots] and (7) size and shape of calcar [i. large and triangular, ii. large and conical, iii. small and conical, iv. small and tubercular, v. absent]. Color in life was obtained from color photographs.

Sound recordings were made with a Sennheiser K6–ME67^TM^ directional microphone with digital recorders Olympus LS-10^TM^ and Marantz PMD660^TM^. Calls were analyzed using Raven 1.3 software (Cornell Lab of Ornithology 2003-2008) at a sampling rate of 44.1 kHz and a frequency resolution of 10.8 Hz. Measured call variables are defined in Table 3. If available, several calls or notes were analyzed per individual to calculate an individual average. Temporal variables were measured on the oscillogram, spectral variables on the power spectrum. Five call variables were used to run a Principal Components Analysis (PCA) to assess the degree of acoustic differentiation between calls from five males of *Hypsiboas fasciatus* (from La Pradera and Zamora), five males of *Hypsiboas almendarizae* sp. n. (from Limón Indanza), seven males of *Hypsiboas calcaratus* (from Estación Científica Yasuní PUCE and Tena), one male of *Hypsiboas maculateralis* sp. n. (from Comunidad Santa Rosa), four males of *Hypsiboas alfaroi* sp. n. (from Estación Científica Yasuní PUCE), and four males of *Hypsiboas tetete* sp. n. (from Comunidad Santa Rosa). Original recordings are deposited in the audio archive of the QCAZ and are available at the AmphibiaWebEcuador website (http://zoologia.puce.edu.ec/vertebrados/anfibios/).

**Table 3.** Call traits of *Hypsiboas* spp. analyzed in this study. See text for details.

Character	Description
Call duration	Time from the beginning of the first note to the end of the last note of the call
Number of notes	Number of notes in the call
Rise time	Time from the beginning of the call to the point of its maximum amplitude
Call dominant frequency	The frequency with the greatest amount of sound energy along all the call
Call fundamental frequency	The frequency with the greatest amount of sound energy in the first harmonic, measured along all the call
Dominant frequency at the beginning to the third note	The frequency with the greatest amount of sound energy measured at the beginning of the third note
Fundamental frequency at the beginning to the third note	The frequency of the first harmonic measured at the beginning of the third note
Dominant frequency at the end to the third note	The frequency with the greatest amount of sound energy measured at the end of the third note
Fundamental frequency at the end to the third note	The frequency of the first harmonic measured at the end of the third note
Number of pulses	Number of pulses per note
Pulse repetition rate	Number of pulses per second
Interval between calls	Time from end of call to the beginning of next call

The conservation status of each species was evaluated according to the IUCN Red List categories and criteria (IUCN 2001). The estimates of the distribution area were based on the minimum convex polygon (the smallest polygon in which no internal angle exceeds 180 degrees and which contains all known localities). Vegetation types are based on WWF Ecoregions (available at http://www.eoearth.org/view/article/151948) except for Ecuadorian localities, which are based in the more detailed classification of Sierra et al. (1999).

### Phylogenetic analyses

Phylogenetic analyses are an expansion of those presented by Funk et al. (2012) to include additional sequences of *Hypsiboas calcaratus*, *Hypsiboas dentei*, *Hypsiboas fasciatus* and *Hypsiboas raniceps* published by Darst and Cannatella (2004), Faivovich et al. (2004, 2005), Salducci et al. (2002, 2005), Jansen et al. (2011), Wiens et al. (2005, 2006) and 19 sequences of the nuclear gene RAG-1 (recombination activating gene 1). GenBank accession numbers for the sequences not included in Funk et al. (2012) are shown in Table 4. The same matrix and alignment of mitochondrial DNA sequence data (genes 12S rRNA, 16S rRNA and COI mtDNA) was employed. For the nuclear DNA, preliminary alignment of RAG-1 and POMC sequences were done with CLUSTAL X 2.0 (Larkin et al. 2007). Manual adjustments to the alignment were made using Mesquite version 2.75 (Maddison and Maddison 2009).

**Table 4.** Genbank accession numbers for DNA sequences used in the phylogenetic analysis. Accession numbers for mitochondrial sequences not included here are listed in Funk et al. (2012).

Museum No.	Species	Genbank Accession No.					Reference
		*12S*	*16S*	*COI*	*POMC*	*RAG1*	
QCAZ 44351	*Hypsiboas alfaroi*	JN970413	JN970549	JN970682	JN970804	KF955320	Funk et al. 2012; This study
QCAZ 44425	*Hypsiboas alfaroi*	JN970415	JN970551	JN970684	JN970806	KF955321	Funk et al. 2012; This study
QCAZ 44858	*Hypsiboas alfaroi*	JN970469	JN970605	JN970737	JN970860	KF955322	Funk et al. 2012; This study
QCAZ 50785	*Hypsiboas alfaroi*	KF955303	KF955305	KF955306	KF955307	--	This study
QCAZ 31452	*Hypsiboas almendarizae*	JN970482	JN970618	--	JN970873	KF955311	Funk et al. 2012; This study
QCAZ 32645	*Hypsiboas almendarizae*	JN970386	JN970522	JN970658	JN970777	KF955312	Funk et al. 2012; This study
QCAZ 39650	*Hypsiboas almendarizae*	JN970394	JN970530	JN970665	JN970785	KF955313	Funk et al. 2012; This study
QCAZ 51809	*Hypsiboas almendarizae*	KF955304	--	--	--	--	This study
QCAZ 43256	*Hypsiboas calcaratus*	JN970444	JN970580	JN970713	JN970835	KF955314	Funk et al. 2012; This study
QCAZ 43789	*Hypsiboas calcaratus*	JN970412	JN970548	JN970681	JN970803	KF955315	Funk et al. 2012; This study
QCAZ 44177	*Hypsiboas calcaratus*	JN970417	JN970553	JN970686	JN970808	KF955316	Funk et al. 2012; This study
KU 202911	*Hypsiboas calcaratus*	AY326056	AY326056	--	--	--	Darst and Cannatella 2004
KU 221856	*Hypsiboas calcaratus*	DQ380352	--	--	--	--	Wiens et al. 2006
13MC	*Hypsiboas dentei*	EF376018	AF467270	--	--	--	Salducci et al. 2002; Salducci et al. 2005
QCAZ 17030	*Hypsiboas fasciatus*	JN970399	JN970535	JN970669	JN970790	KF955310	Funk et al. 2012; This study
QCAZ 48583	*Hypsiboas fasciatus*	JN970490	JN970626	--	JN970881	KF955308	Funk et al. 2012; This study
QCAZ 48584	*Hypsiboas fasciatus*	JN970388	JN970524	--	JN970779	KF955309	Funk et al. 2012; This study
QCAZ 20641	*Hypsiboas lanciformis*	JN970512	JN970648	JN970767	JN970898	KF955325	Funk et al. 2012; This study
QCAZ 30936	*Hypsiboas lanciformis*	JN970510	JN970646	JN970765	JN970896	KF955326	Funk et al. 2012; This study
QCAZ 40082	*Hypsiboas maculateralis*	JN970405	JN970541	JN970675	JN970796	KF955317	Funk et al. 2012; This study
QCAZ 44248	*Hypsiboas maculateralis*	JN970423	JN970559	JN970692	JN970814	KF955318	Funk et al. 2012; This study
QCAZ 44452	*Hypsiboas maculateralis*	JN970416	JN970552	JN970685	JN970807	KF955319	Funk et al. 2012; This study
115MC	*Hypsiboas raniceps*	EF376021	AF467269	--	--	--	Salducci et al. 2002; Salducci et al. 2005
MACN 37795	*Hypsiboas raniceps*	AY843657	AY843657	--	--	--	Faivovich et al. 2005
USNM 174173	*Hypsiboas raniceps*	AY819375	--	--	--	--	Wiens et al. 2005
QCAZ 40080	*Hypsiboas tetete*	JN970403	JN970539	JN970673	JN970794	KF955323	Funk et al. 2012; This study
QCAZ 40081	*Hypsiboas tetete*	JN970404	JN970540	JN970674	JN970795	KF955324	Funk et al. 2012; This study
MNKA 9467	*Hypsiboas* sp.	--	JF790135	--	--	--	Jansen et al. 2011
MNKA 9468	*Hypsiboas* sp.	--	JF790136	--	--	--	Jansen et al. 2011
MNKA 9469	*Hypsiboas* sp.	--	JF790137	--	--	--	Jansen et al. 2011
MNKA 9477	*Hypsiboas* sp.	--	JF790138	--	--	--	Jansen et al. 2011
AMNH-A 164081	*Hypsiboas* sp.	--	AY549335	--	--	--	Faivovich et al. 2004
NMP6V 71250	*Hypsiboas* sp.	--	AY843613	--	--	--	Faivovich et al. 2005

Because it is likely that each of our sampled genes (or codon positions in protein coding genes) evolved under different processes, we partitioned the matrices according to gene and codon position to analyze each partition under separate models of evolution. We used PartitionFinder v. 1.1.1 (Lanfear et al. 2012) to find the best-fit model for each partition and also the best partition strategy. In the mitochondrial matrix, we defined five *a priori* partitions (12S, 16S and one partition for each codon position of COI). In the nuclear matrix we defined six *a priori* partitions (corresponding to each codon position of our two genes).

Phylogenetic relationships were inferred separately for mitochondrial and nuclear genes using maximum-likelihood and Bayesian inference. Maximum-likelihood and Bayesian analyses were conducted in GARLI v. 2.0 (Zwickl 2006) and MRBAYES v. 3.2.1 (Ronquist et al. 2012) respectively using the same methodology described by Funk et al. (2012).

## Results

### Phylogenetic relationships

The phylogenetic relationships recovered from the analysis of the mitochondrial DNA sequences (Fig. 1) were consistent with those reported by Funk et al. (2012). The following sections describe the relationships of the samples not included in Funk et al. (2012).

**Figure 1.**
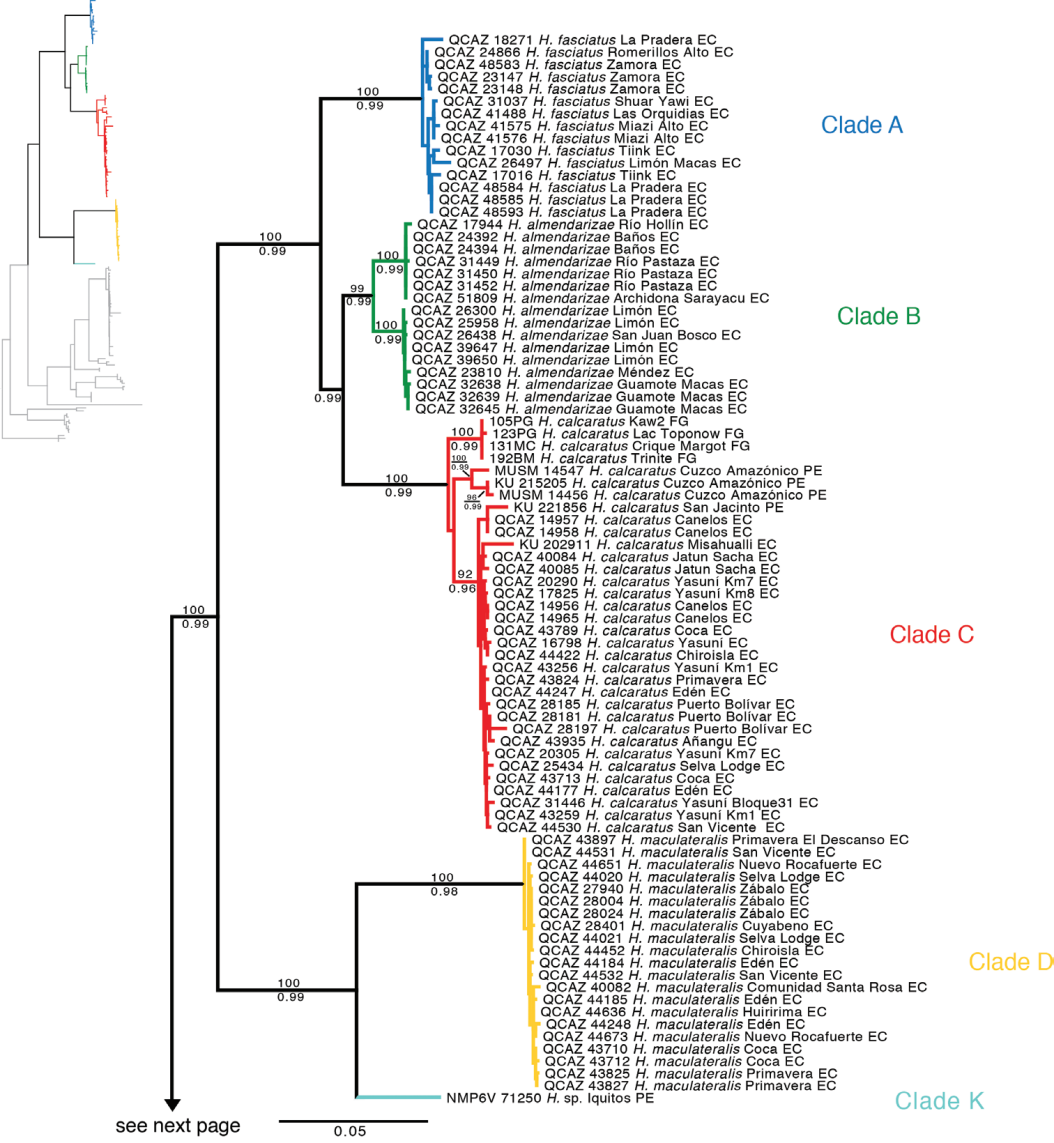
Maximum likelihood phylogram depicting relationships within *Hypsiboas*. The phylogram was derived from analysis of 2400 bp of mitochondrial DNA (gene fragments *12S*, *16S*, *COI*). Museum catalog number and locality are shown for each sample. Bootstrap values are shown above the branches and Bayesian posterior probabilities are shown below; missing values indicate values below 50 (bootstrap) or 0.5 (posterior probability). Outgroup species (*Hypsiboas lanciformis*, *Hypsiboas pellucens* and*Hypsiboas rubracylus*) are not shown. Abbreviations are: **ARG** Argentina, **BO** Bolivia, **EC** Ecuador, **FG** French Guiana, **GUY** Guyana, **PE** Peru, **SU** Suriname.

**Figure 1.**
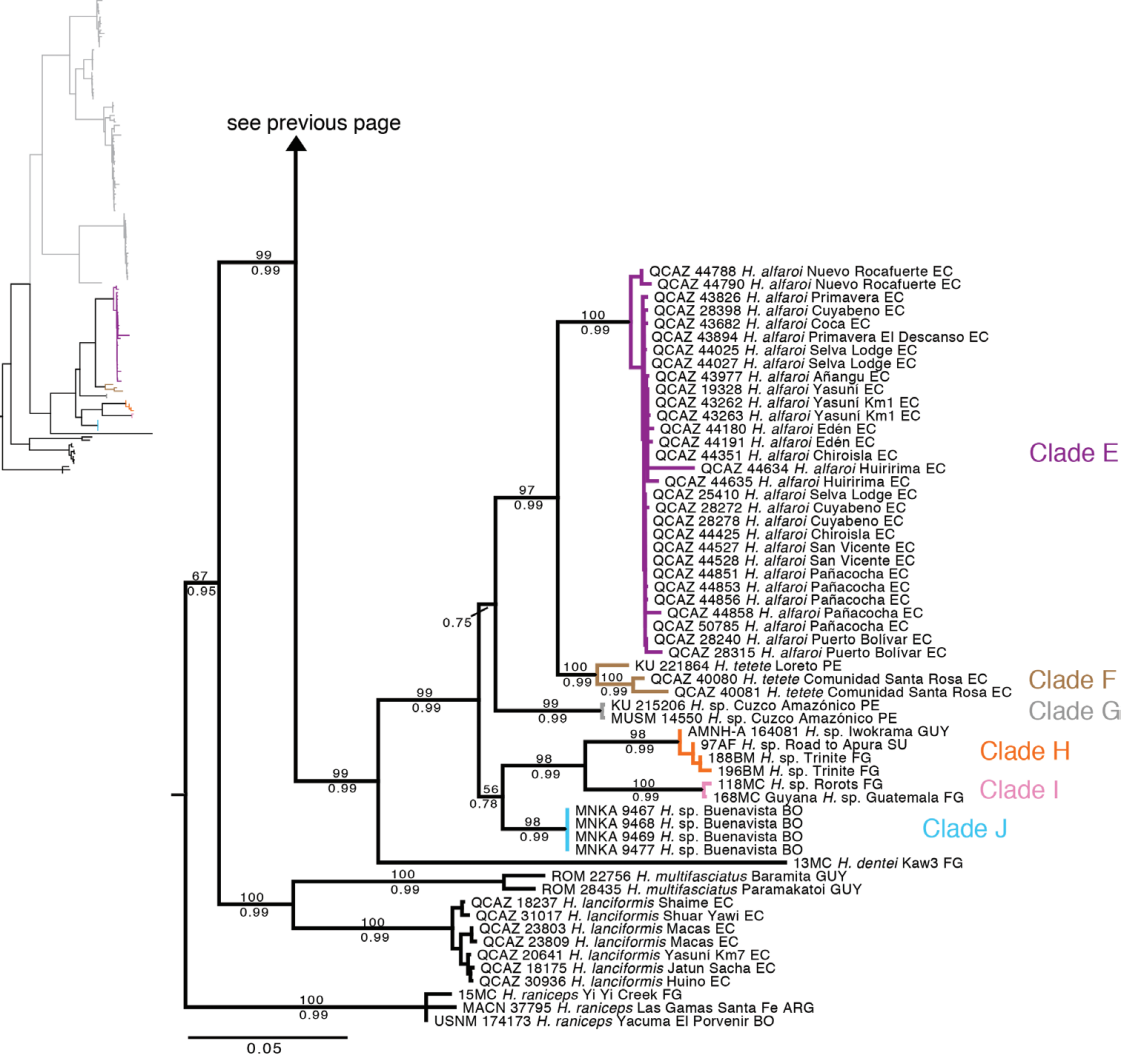
Continue

We found strong support for a clade that includes *Hypsiboas dentei* and the *Hypsiboas calcaratus*-*Hypsiboas fasciatus* species complex. This clade is sister to *Hypsiboas lanciformis* + *Hypsiboas multifasciatus*. There are eleven candidate species, two more than those reported by Funk et al. (2012). The population from Bolivia (clade J) is sister to clades I and H from French Guiana, Guyana, and Suriname. The branch lengths in the phylogeny and the genetic distances between J and I-H (uncorrected *p*-distance range 3.1–5.8% in gen 16S; Table 5) suggest that J is not conspecific to any of the candidate species reported by Funk et al. (2012). An additional unconfirmed candidate species (clade K) is represented by a single sample (NMP6V 71250; reported as *Hypsiboas calcaratus* by Faivovich et al. 2005) from 50 km W of Iquitos (Peru). The genetic distance relative to its closest relative (clade D) ranges from 4.8 to 5.9% (16S), strongly suggesting that it is a separate species. The branch lengths that separate clade K from clade D are longer than the lengths that separate pairs of some confirmed candidate species (e.g., *Hypsiboas fasciatus* vs. *Hypsiboas almendarizae* sp. n.) further implying that K is a valid undescribed species.

**Table 5.** Pairwise genetic distances (uncorrected *p*) of 16S DNA sequences among members of the *Hypsiboas calcaratus* species complex. Mean ± SD is given with range in parentheses (below diagonal). Number of individuals compared is shown above diagonal. Diagonal shows intra-clade genetic distances.

	Clade A *Hypsiboas fasciatus*	Clade B *Hypsiboas almendarizae*	Clade C *Hypsiboas calcaratus*	Clade D *Hypsiboas maculateralis*	Clade E *Hypsiboas alfaroi*	Clade F *Hypsiboas tetete*	Clade G	Clade H	Clade I	Clade J	Clade K
**Clade A** ***Hypsiboas fasciatus***	0.004 ± 0.004 (0.007–0.017)	*n* = 31	*n* = 49	*n* = 36	*n* = 45	*n* = 18	*n* = 17	*n* = 19	*n* = 17	*n* = 19	*n* = 16
**Clade B** ***Hypsiboas almendarizae***	0.036 ± 0.009 (0.035–0.049)	0.008 ± 0.0006 (0.001–0.012)	*n* = 50	*n* = 37	*n* = 46	*n* = 19	*n* = 18	*n* = 20	*n* = 18	*n* = 20	*n* = 17
**Clade C** ***Hypsiboas calcaratus***	0.057 ± 0.011 (0.038–0.078)	0.041 ± 0.003 (0.034–0.050)	0.009 ± 0.008 (0.001–0.031)	*n* = 55	*n* = 64	*n* = 37	*n* = 36	*n* = 38	*n* = 36	*n* = 38	*n* = 35
**Clade D** ***Hypsiboas maculateralis***	0.077 ± 0.003 (0.072–0.100)	0.074 ± 0.003 (0.069–0.086)	0.086± 0.006 (0.064–0.102)	0.002 ± 0.0008 (0.001–0.004)	*n* = 51	*n* = 24	*n* = 23	*n* = 25	*n* = 23	*n* = 25	*n* = 22
**Clade E** ***Hypsiboas alfaroi***	0.082 ± 0.004 (0.074–0.100)	0.080 ± 0.003 (0.072–0.090)	0.089 ± 0.004 (0.073–0.105)	0.107 ± 0.004 (0.097–0.120)	0.002 ± 0.002 (0.003–0.017)	*n* = 33	*n* = 32	*n* = 34	*n* = 32	*n* = 34	*n* = 31
**Clade F** ***Hypsiboas tetete***	0.085 ± 0.002 (0.083–0.095)	0.078 ± 0.001 (0.073–0.082)	0.084 ± 0.003 (0.074–0.096)	0.100 ± 0.002 (0.093–0.107)	0.028 ± 0.002 (0.023–0.037)	0.013 ± 0.009 (0.001–0.019)	*n* = 5	*n* = 7	*n* = 5	*n* = 7	*n* = 4
**Clade G**	0.082 ± 0.002 (0.081–0.092)	0.077 ± 0.002 (0.073–0.079)	0.085 ± 0.003 (0.075–0.091)	0.097 ± 0.003 (0.091–0.107)	0.039 ± 0.002 (0.033–0.045)	0.041 ± 0.001 (0.039–0.043)	0	*n* = 6	*n* = 4	*n* = 6	*n* = 3
**Clade H**	0.088 ± 0.003 (0.083–0.101)	0.082 ± 0.002 (0.077–0.087)	0.095 ± 0.003 (0.088–0.108)	0.098 ± 0.003 (0.094–0.107)	0.055 ± 0.003 (0.050–0.068)	0.053 ± 0.002 (0.049–0.056)	0.052 ± 0.002 (0.050–0.055)	0.004 ± 0.002 (0.001–0.007)	*n* = 6	*n* = 8	*n* = 5
**Clade I**	0.088 ± 0.004 (0.081–0.097)	0.083 ± 0.003 (0.077–0.089)	0.098 ± 0.004 (0.093–0.115)	0.097 ± 0.005 (0.091–0.112)	0.059 ± 0.002 (0.056–0.067)	0.056 ± 0.001 (0.055–0.058)	0.058 ± 0.001 (0.057–0.059)	0.031 ± 0.002 (0.028–0.034)	0.003	*n* = 6	*n* = 3
**Clade J**	0.081 ± 0.002 (0.077–0.087)	0.082 ± 0.003 (0.079–0.090)	0.084 ± 0.004 (0.079–0.097)	0.094 ± 0.004 (0.091–0.108)	0.053 ± 0.004 (0.049–0.075)	0.052 ± 0.002 (0.048–0.055)	0.035 ± 0.0008 (0.034–0.035)	0.038 ± 0.005 (0.031–0.045)	0.056 ± 0.002 (0.054–0.058)	0	*n* = 5
**Clade K**	0.073 ± 0.002 (0.071–0.081)	0.068 ± 0.001 (0.065–0.071)	0.072 ± 0.008 (0.047–0.085)	0.051 ± 0.004 (0.048–0.059)	0.101 ± 0.004 (0.094–0.112)	0.099 ± 0.001 (0.098–0.100)	0.097	0.102 ± 0.002 (0.100–0.105)	0.103	0.086 ± 0.0002 (0.085–0.086)	0

**Figure 2.**
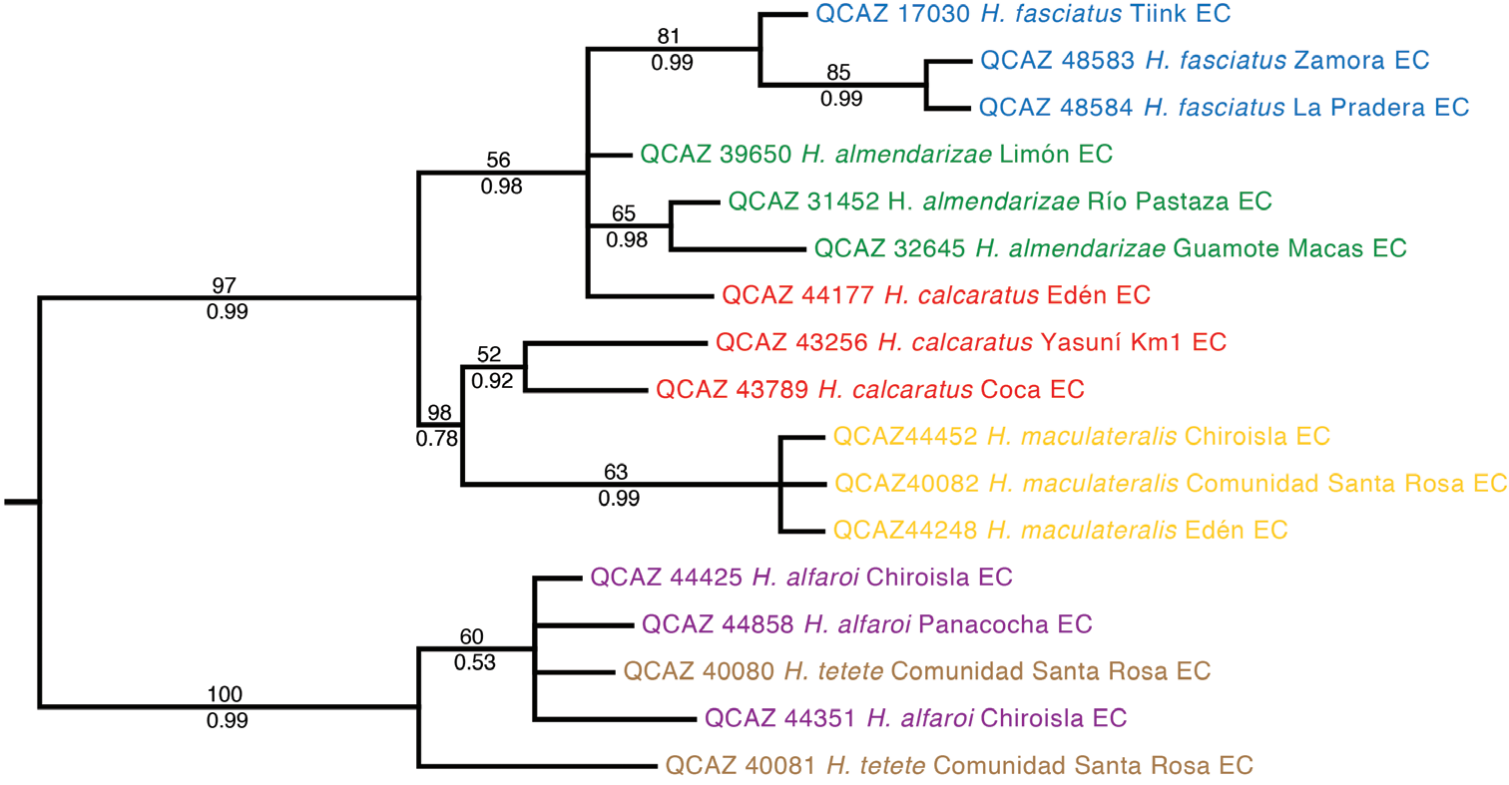
Maximum likelihood phylogram depicting relationships within *Hypsiboas*. The phylogram was derived from analysis of 1272 bp of nuclear DNA (gene fragments *RAG-1* and *POMC*). Museum catalog number and locality are shown for each sample. Bootstrap values are shown above the branches and Bayesian posterior probabilities are shown below; values below 50 (bootstrap) or 0.5 (posterior probability) are not shown. Colors refer to clades identified using mtDNA (see Figure 1).

The phylogeny based on the nuclear genes (RAG-1 and POMC) is generally consistent with the mitochondrial phylogeny but is less resolved (Fig. 2). Similarly to the mitochondrial phylogeny, it shows strong support for two basal clades (Clades A–D and Clades E–F). At the species level, it provides strong support for clades A and D.

The only strongly supported disagreement between the nuclear and the mitochondrial phylogeny is the placement of individual QCAZ 44177 because in the nuclear tree it appears within a clade formed by individuals from the mitochondrial clades A and B, while in the mitochondrial tree it appears as a member of clade C. The morphological data shows that QCAZ 44177 is a member of clade C because it has its distinctive characters (e.g., large and triangular calcar).

## Taxonomic review

The available names for the populations sampled in our phylogenies are *Hypsiboas calcaratus* (Troschel, 1848), *Hypsiboas fasciatus* (Günther, 1858), *Hyla leptoscelis* Boulenger, 1918, and *Hyla steinbachi* Boulenger, 1905. Examination of three of the holotypes and published descriptions from the literature allowed us to assign the available names to clades A and C (Fig. 1). We document those assignments in the following section.

### Taxonomic status of *Hypsiboas fasciatus* and *Hypsiboas calcaratus*

**Figure 3.**
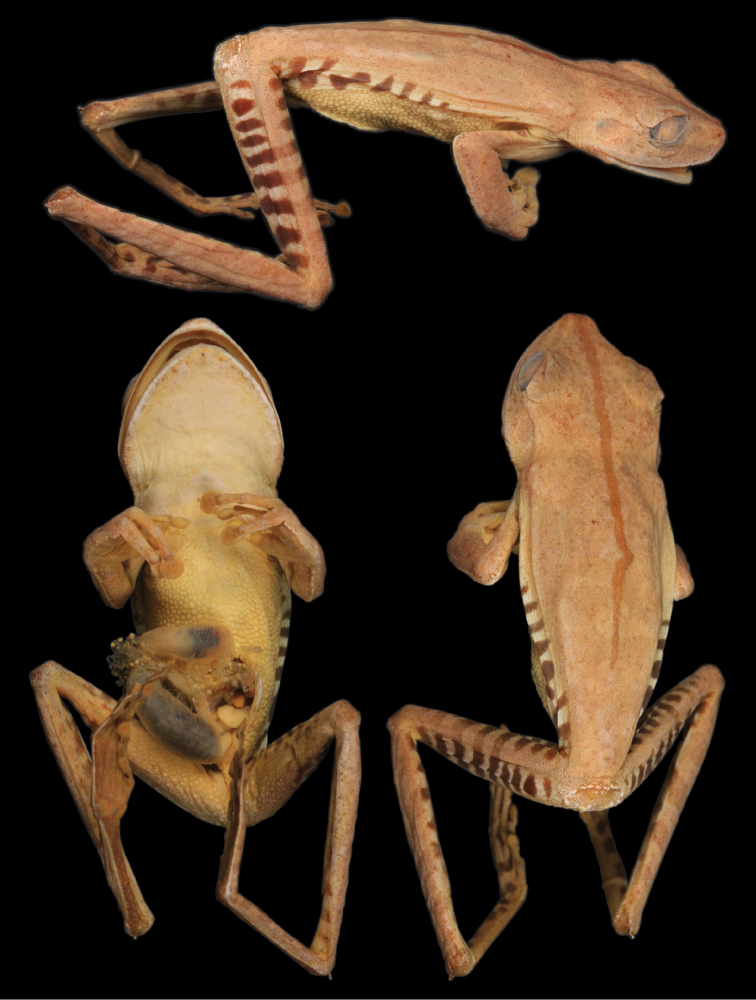
Dorsolateral (top), ventral (left), and dorsal (right) views of the holotype of *Hypsiboas fasciatus* (BMNH 58.4.25.22).

**Figure 4.**
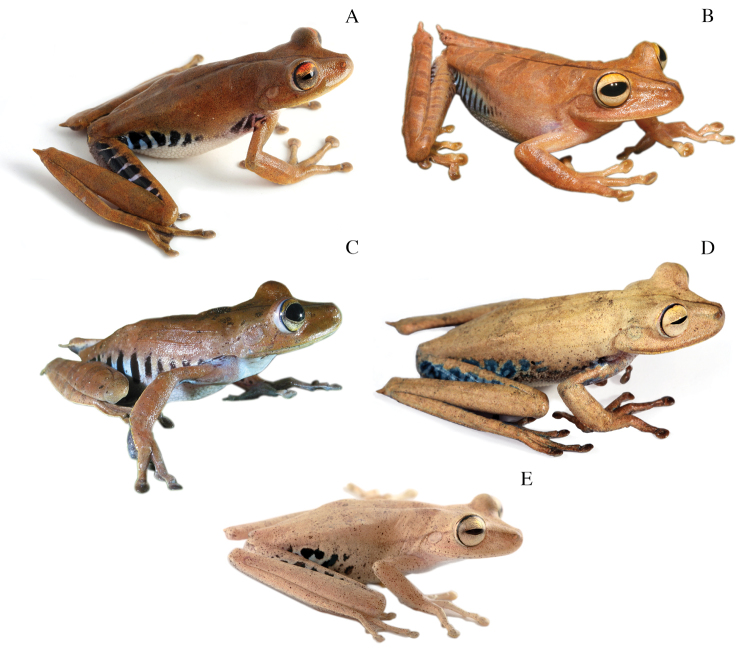
Dorsolateral views of adult females of **A** *Hypsiboas fasciatus*, QCAZ 48611, SVL = 51.79 mm **B** *Hypsiboas almendarizae*, QCAZ 32638, SVL = 51.26 mm **C** *Hypsiboas calcaratus*, QCAZ 24282, SVL = 51.26 mm **D** *Hypsiboas maculateralis*, QCAZ 43825, SVL = 55.31 mm **E** *Hypsiboas alfaroi*, QCAZ 43252, SVL = 45.37 mm.

**Figure 5.**
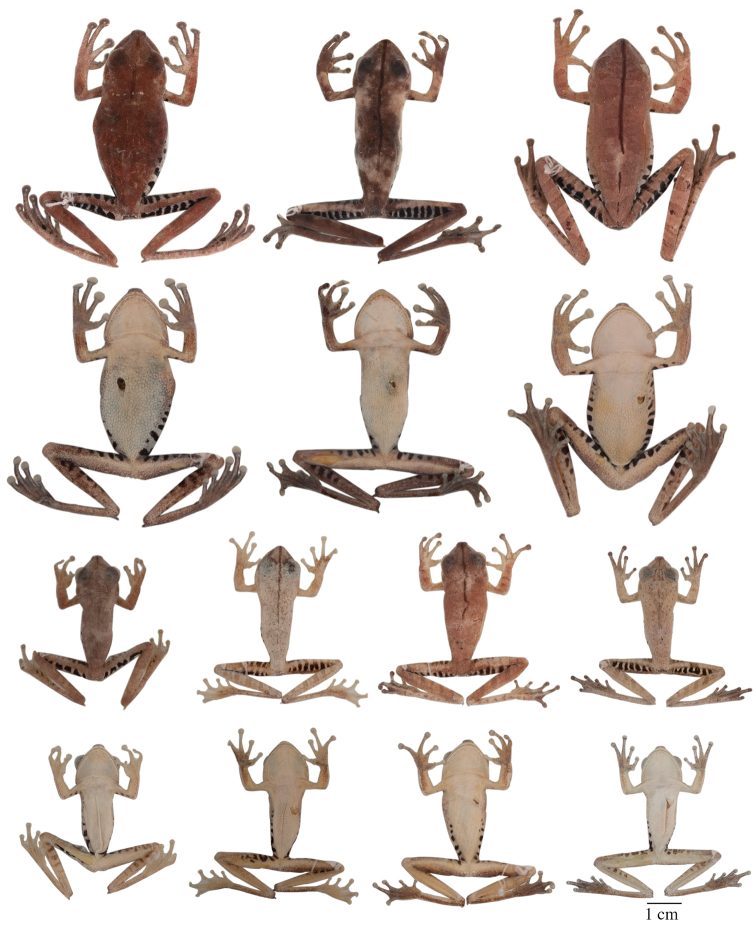
Adult *Hypsiboas fasciatus* showing variation in dorsal and ventral coloration of preserved specimens. From left to right, first and second rows: QCAZ 24866, 26497, 41575 (females); third and fourth rows: QCAZ 18271, 41488, 41576, 48584 (males). See Appendix for locality data. All specimens are shown at the same scale.

**Figure 6.**
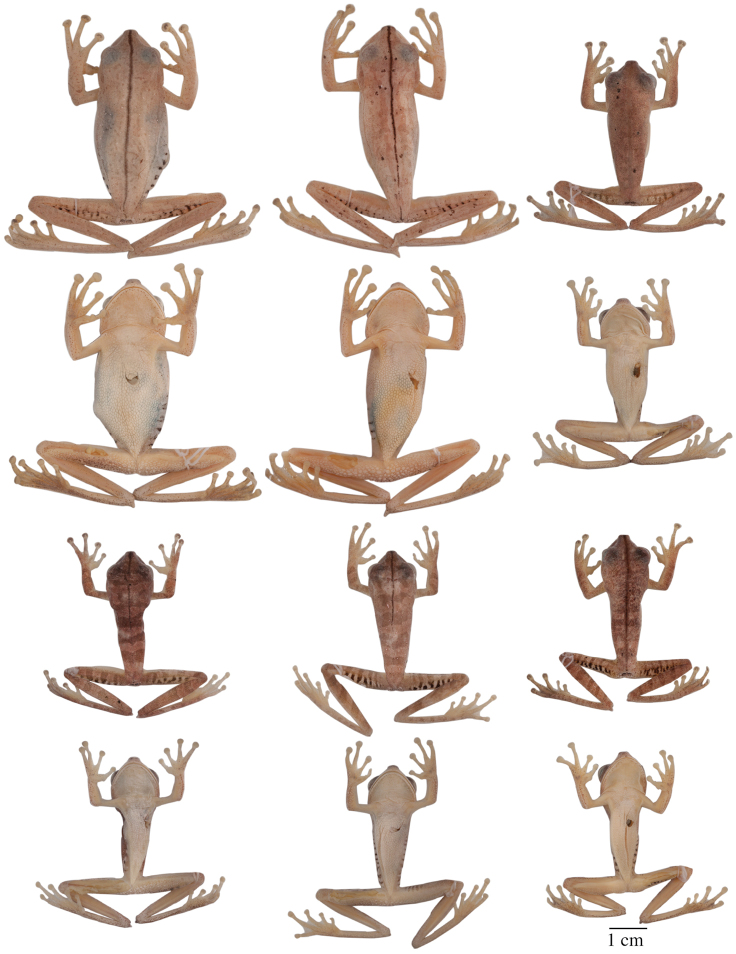
Adult *Hypsiboas almendarizae* showing variation in dorsal and ventral coloration of preserved specimens. From left to right, first and second rows: QCAZ 32638, 32645 (females), 24392 (male); third and fourth rows: QCAZ 39647, 39650, 24394 (males). See Appendix for locality data. All specimens are shown at the same scale.

**Figure 7.**
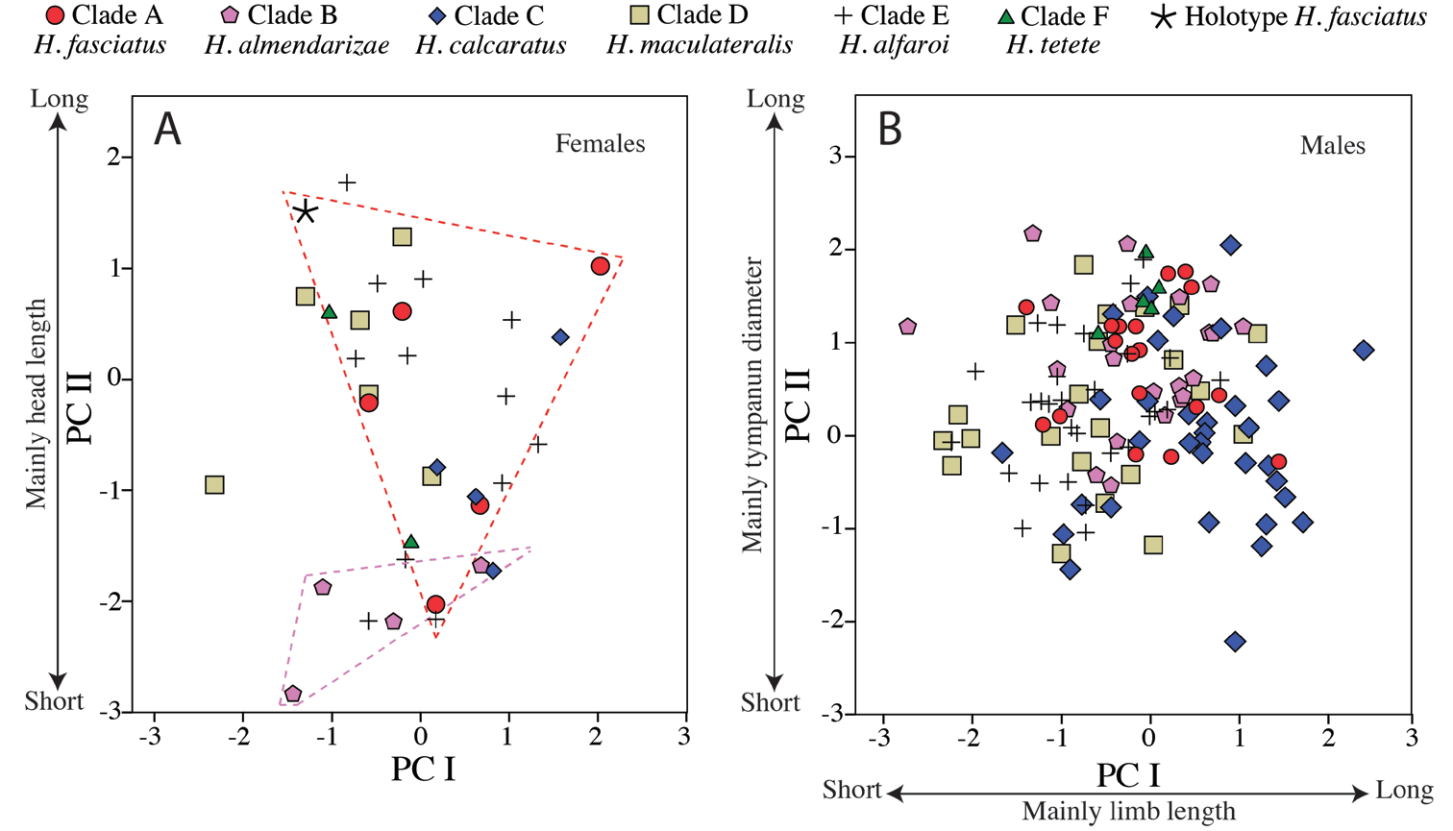
Principal components from analysis of six size-corrected morphological variables for **A** 34 females and **B** 136 males of *Hypsiboas*. See Table 8 for character loadings on each component.

The holotype of *Hypsiboas fasciatus* is an adult female (SVL = 58.59 mm; BM 1858.7.25.22, reported as “BM 58.4.25.22” by Duellman 1973; Fig. 3) collected by Louis Fraser. It closely resembles individuals from clade A and B (Fig. 4A–B), which also have a small calcar and black bars on the flanks and thighs (Figs 5 and 6). We assign the binomial *Hypsiboas fasciatus* to clade A based on morphometric data showing a closer affinity to the holotype (Fig. 7A). Clade B (*Hypsiboas almendarizae* sp. n.) further differs from the holotype in having narrower transversal dark bars on the flanks and thighs. The assignment of *Hypsiboas fasciatus* to clade A is also supported by geographic distribution. Although in the species description by Günther (1858) the type locality is vaguely stated as “Anden von Ecuador”, it is almost certain that the holotype was collected either in Gualaquiza or Zamora, Morona Santiago and Zamora Chinchipe provinces, Ecuador. We infer this from letters and records of the collections of the travels of Louis Fraser (Gardner 1983 and references therein). During his trip, which lasted from 1857 to 1859, the only collection sites within the distribution range of the *Hypsiboas fasciatus*-*Hypsiboas calcaratus* species complex are Gualaquiza (elevation 1100 m) and Zamora (1000 m). Both localities overlap with the distribution range of clade A exclusively. Clade A has an elevation range from 700 to 1600 m.

**Figure 8.**
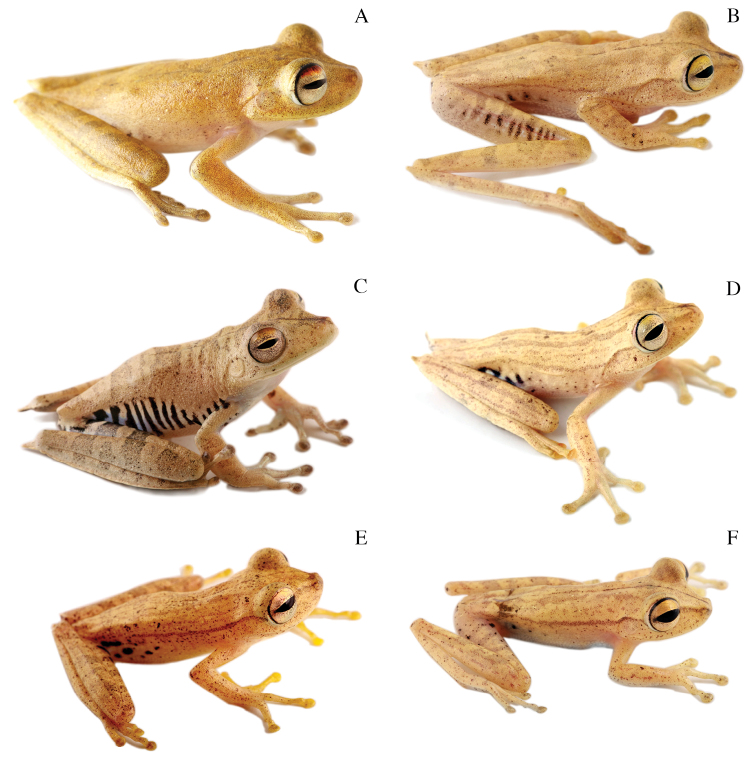
Dorsolateral views of adult males of **A** *Hypsiboas fasciatus*, QCAZ 48584, SVL = 33.77 mm **B** *Hypsiboas almendarizae*, QCAZ 39649, SVL = 36.54 mm **C** *Hypsiboas calcaratus*, QCAZ 43256, SVL = 40.07 mm **D** *Hypsiboas maculateralis*, QCAZ 40082, SVL = 36.16 mm **E** *Hypsiboas alfaroi*, QCAZ 43260, SVL = 30.35 mm **F** *Hypsiboas tetete*, QCAZ 40081, SVL = 31.15 mm.

Duellman (1973) characterized *Hypsiboas fasciatus* as having, in the majority of the specimens, brown flecks on the throat and chest and black spots on the flanks and thighs. The holotype of *Hypsiboas fasciatus* differs from this diagnosis because it has dark bars instead of dots on the flanks and thighs and lacks flecks on the throat and chest. Most of the specimens reported by Duellman (1973) as “*Hypsiboas fasciatus*” are from localities below 700 m and resemble individuals from our clades D, E, and F (Figs 4D–E and 8D–F). Although Duellman (1973) noted the aforementioned differences between the holotype and the other individuals, he considered them conspecific.

**Figure 9.**
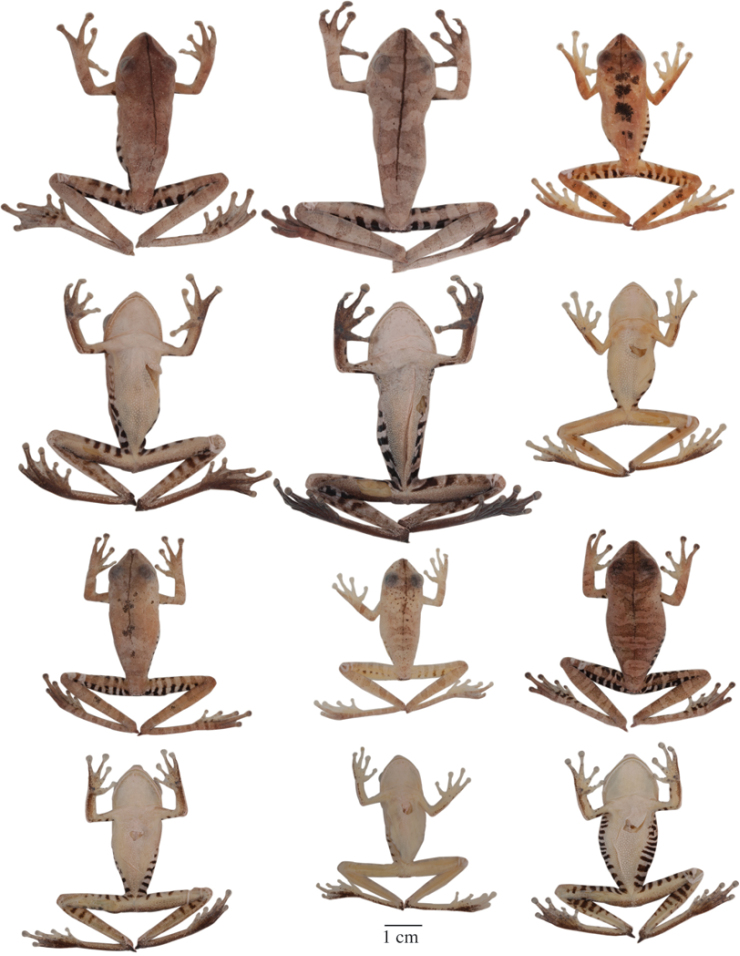
Adult *Hypsiboas calcaratus* showing variation in dorsal and ventral coloration of preserved specimens. From left to right, first and second rows: QCAZ 44422, 44530 (females), 14957 (male); third and fourth rows: QCAZ 43259, 40085, 43256 (males). See Appendix for locality data. All specimens are shown at the same scale.

The holotype of *Hypsiboas calcaratus* could not be examined because it is lost (Duellman 1973; Frost 2013). Nevertheless, the description of the holotype of *Hypsiboas calcaratus* by Troschel (1848) and the location of the type locality (British Guiana) suggest that the binomial *Hypsiboas calcaratus* is conspecific to clade C (Fig. 1). The holotype was described as having a long calcar, perpendicular short black stripes on the flanks, and transversal bars on the legs, which are characteristic of clade C. This designation is also supported by the type locality “British Guiana” because it overlaps with the distribution of clade C. The presence of clade C in the Republic of Guyana (formerly known as British Guiana) is documented by photographs and the morphological description of “*Hypsiboas calcaratus*” by Kok and Kalamandeen (2008). Clade C is the only species with developed calcar and dark bars on the legs and flanks that occurs below 500 m (Fig. 9). Based on the available evidence, we assign the binomial *Hypsiboas calcaratus* to clade C.

## Taxonomic status of *Hyla leptoscelis* and *Hyla steinbachi*

**Figure 10.**
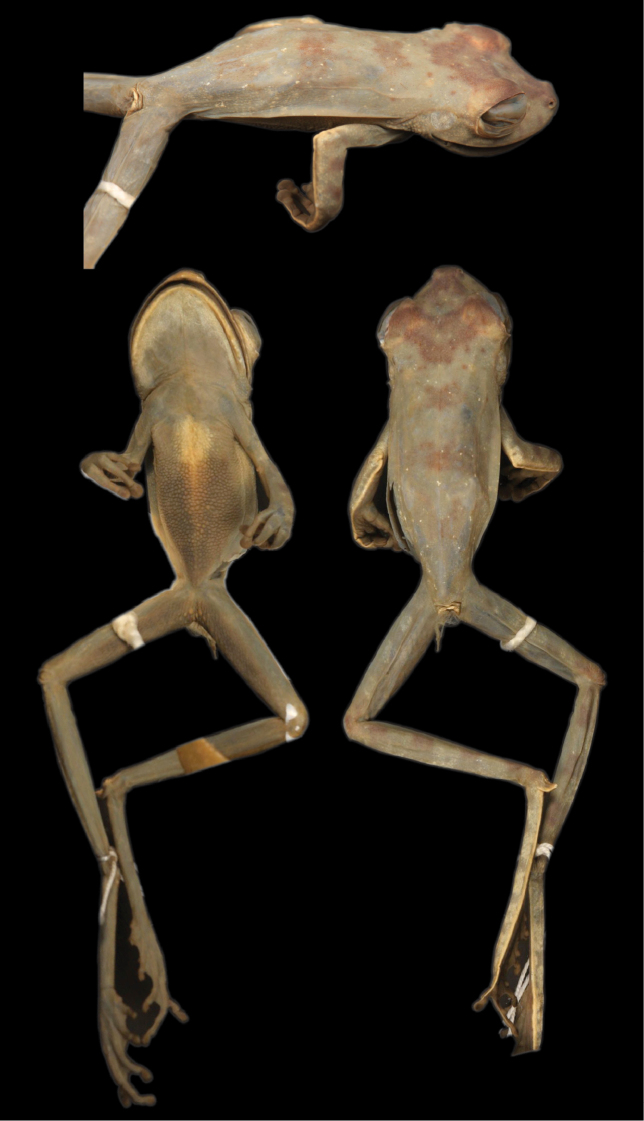
Holotype of *Hyla leptoscelis* (BM 1947.2.23.10). Above: dorsolateral view; below: ventral (left) and dorsal (right) views.

Other available names for the *Hypsiboas fasciatus*-*Hypsiboas calcaratus* species group are *Hyla leptoscelis* Boulenger, 1918 and *Hyla steinbachi* Boulenger, 1905. *Hyla leptoscelis* was synonymized under *Hypsiboas calcaratus* by Duellman (1973). The holotype is a juvenile female with SVL = 27 mm (BM 1947.2.23.10; Fig. 10). As noted by Duellman (1973) and Lutz (1973), the specimen is in poor condition, discolored, and predominantly gray. However, it has large calcars and lacks webbing on the hands, indicating that it belongs to *Hypsiboas calcaratus* and, thus, the synonymy proposed by Duellman (1973) seems to be correct.

**Figure 11.**
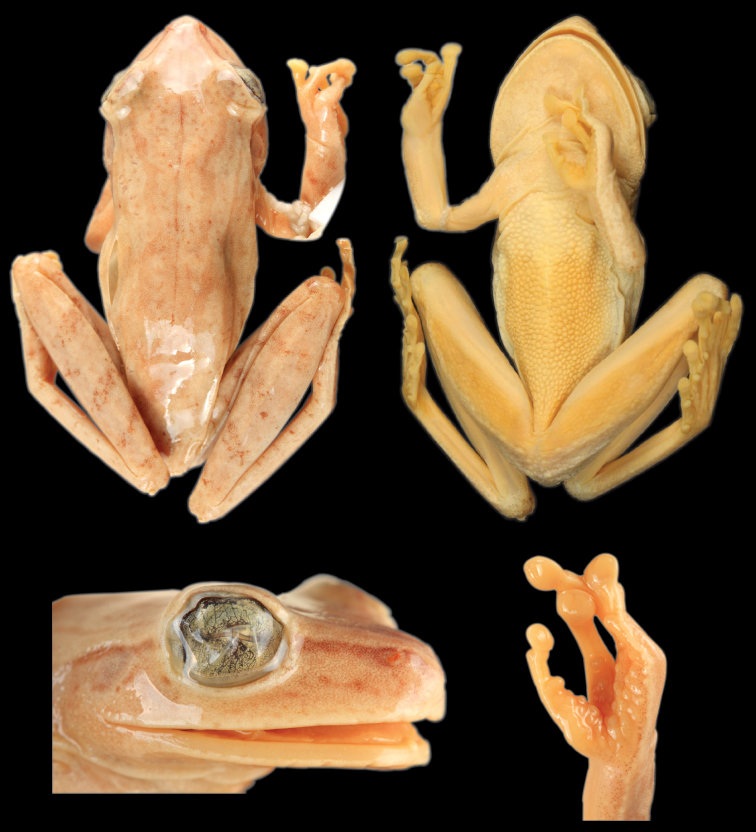
Syntype of *Hyla steinbachi* (BM 1947.2.13.61). Above: dorsal (left) and ventral (right) views; below: lateral view of head and ventral view of left hand.

*Hyla steinbachi* Boulenger, 1905 (Fig. 11) was synonymized under *Hyla fasciata* by De la Riva (1990). There are three syntypes, two are adults of unknown sex (BM 1947.2.13.61 and BM 1947.2.13.62; SVL 36.20 and 32.85 mm, respectively) and one is a juvenile (BM 1947.2.13.63, SVL 22.04 mm). The adult specimens have ill-defined calcars and prominent and abundant supernumerary tubercles on the hands (Fig. 11). These characters distinguish it from *Hypsiboas fasciatus*, *Hypsiboas calcaratus*, and the new species described herein. Thus, we remove it from its synonymy with *Hypsiboas fasciatus* and assign it to the genus *Hypsiboas*: *Hypsiboas steinbachi* new. comb.

## Systematic accounts

### Hypsiboas calcaratus

(Troschel, 1848)

http://species-id.net/wiki/Hypsiboas_calcaratus

Hyla calcarata Troschel, 1848: 660. Type material not designated and likely lost. Type locality “Britisch-Guiana” (= Guyana; Frost 2013).

#### Diagnosis.

*Hypsiboas calcaratus* (Figs 4C, 8C, and 9) is characterized by: (1) mean SVL 36.82 mm in males (range 27.61–42.50; *n* = 36), 50.92 mm in females (range 45.94–56.29; *n* = 4); (2) basal webbing on the fingers; (3) calcar large and triangular; (4) dorsal background color ranging from reddish brown to cream, pinkish white or grayish brown, in most cases dark marks are present (e.g., broad transversal marks, large black stains); (5) often middorsal dark brown line present; (6) flanks pale cream or gray (in life, blue in large females and light blue or white in males) with dark brown vertical bars; (7) hidden surfaces of thighs pale cream or gray (in life, blue in large females and light blue or white in males) with dark brown transversal bars; (8) ventral surfaces of thighs creamy white, yellowish white or brown; (9) venter creamy white or yellowish white; (10) webbing on feet; (11) in life, iris creamy silver or bronze with upper yellow to orange band; (12) prepollical spine present in males.

**Figure 12.**
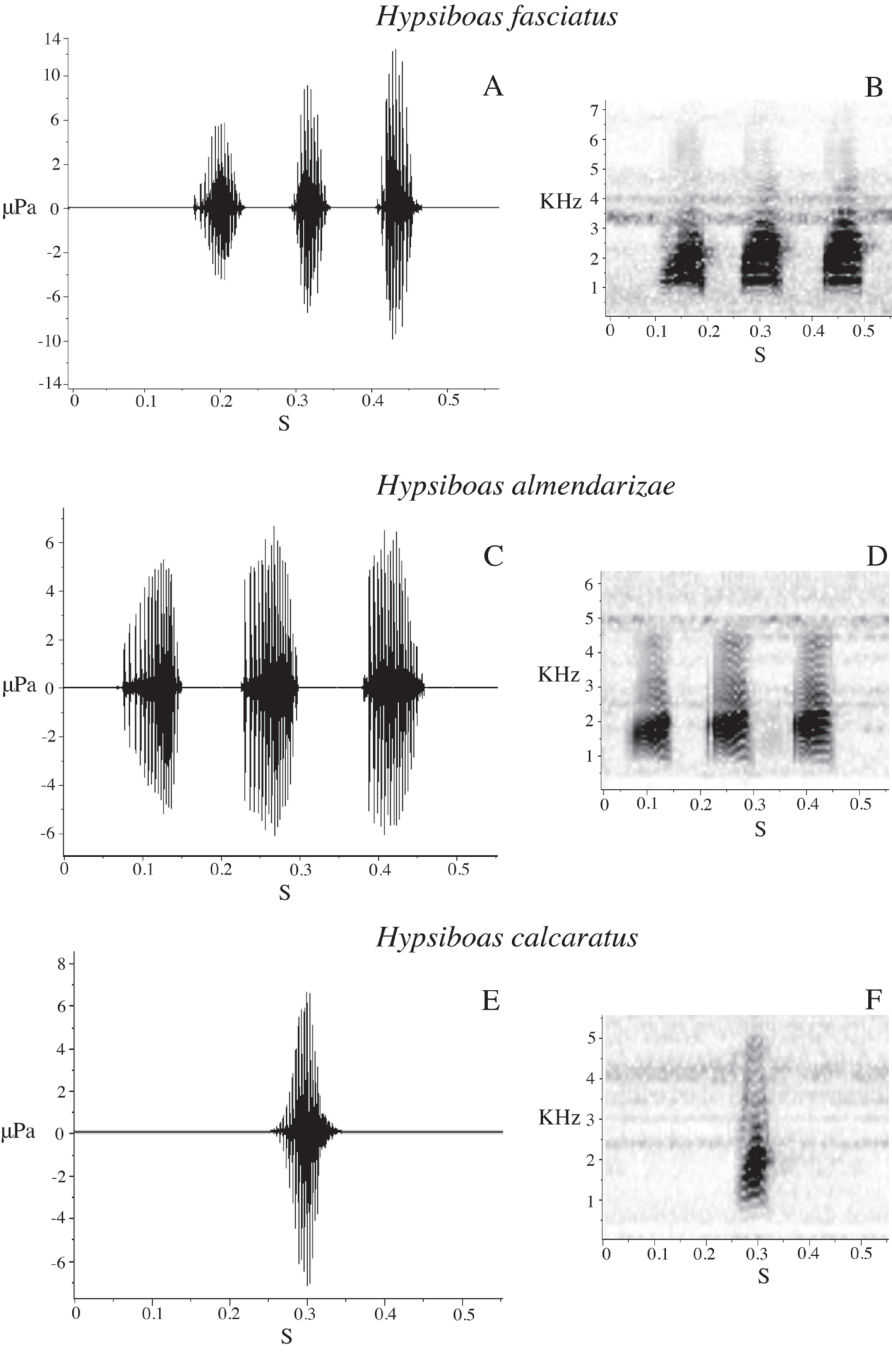
Calls of the *Hypsiboas calcaratus* species complex. **A–B** *Hypsiboas fasciatus* (QCAZ 48585) from La Pradera, Provincia Morona Santiago **C–D** *Hypsiboas almendarizae* (QCAZ 39650) from Limón, Provincia Morona Santiago **E–F** *Hypsiboas calcaratus* (QCAZ 40085) from Tena, Provincia Napo. **A, C, E** are oscilograms and **B, D, F** spectrograms.

*Hypsiboas calcaratus* is most similar to *Hypsiboas fasciatus* and *Hypsiboas almendarizae* sp. n. It differs from both species by the shape of the calcar (large and triangular in *Hypsiboas calcaratus*, small and conical in *Hypsiboas fasciatus*, and large and conical in *Hypsiboas almendarizae* sp. n.; Fig. 14C–D) and by the number of notes in the advisement call (Fig. 12). *Hypsiboas calcaratus* can be further distinguished from *Hypsiboas fasciatus* by the color of the upper band in the iris: red to reddish brown in *Hypsiboas fasciatus*, yellow to orange in *Hypsiboas calcaratus*. *Hypsiboas almendarizae* sp. n. differs from *Hypsiboas calcaratus* in having narrower transversal dark bars on the flanks and thighs (mean width of bars on thighs = 5.05% of femur length, SD = 0.61, in *Hypsiboas almendarizae* sp. n. vs. 7.89%, SD = 1.2, in *Hypsiboas calcaratus*; differences are significant: *t* = -6.72, df = 18, *P* < 0.001) and smaller calcars.

**Figure 13.**
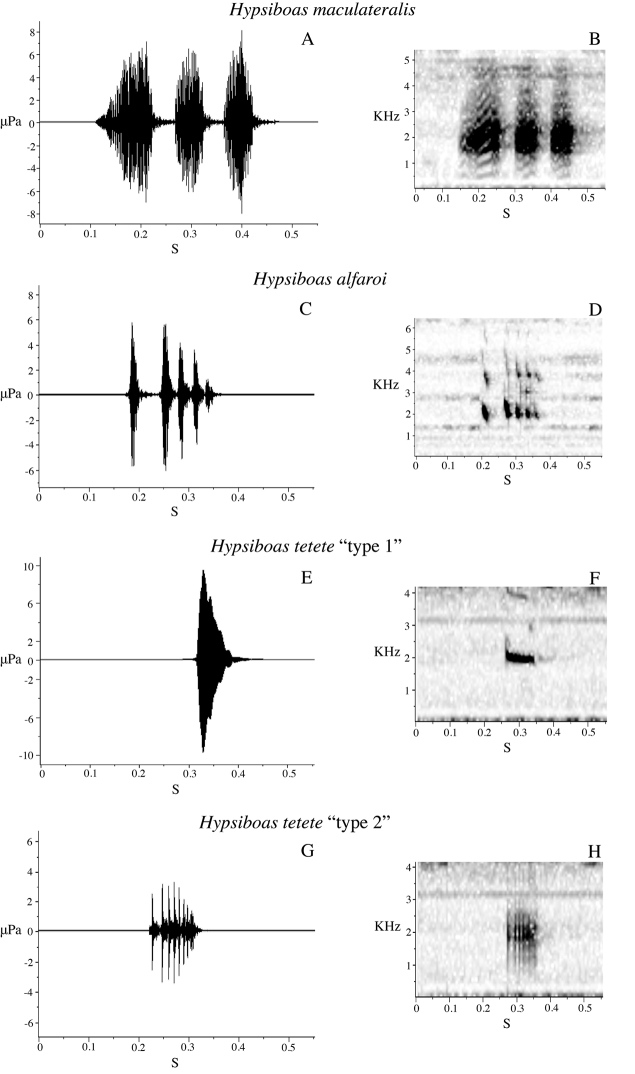
Calls of the *Hypsiboas calcaratus* species complex. **A–B** *Hypsiboas maculateralis* (QCAZ 40082) from Comunidad Santa Rosa, Provincia Napo **C–D** *Hypsiboas alfaroi* (QCAZ 43260) from Estación Científica Yasuní PUCE, Provincia Orellana **E–H** *Hypsiboas tetete* (QCAZ 40081) from Comunidad Santa Rosa, Provincia Napo. **A, C, E, G** are oscilograms and **B, D, F, H** spectrograms.

*Hypsiboas calcaratus* differs from *Hypsiboas maculateralis* sp. n. in advertisement call (lower dominant frequency, higher fundamental frequency; Figs 12E–F and 13A–B) and by the presence of transversal bars on the flanks and hidden surfaces of the thighs (dark blotches instead of bars in *Hypsiboas maculateralis* sp. n.) *Hypsiboas calcaratus* can be distinguished from *Hypsiboas alfaroi* sp. n. and *Hypsiboas tetete* sp. n. by the presence of a calcar (instead of a small tubercle on the heel) and by the absence of dark flecks on the gular region and chest (present in *Hypsiboas alfaroi* sp. n. and *Hypsiboas tetete*. sp. n.) Morphological characters useful to differentiate *Hypsiboas calcaratus* from other species are shown in Table 6.

**Table 6.** Diagnostic characters of male specimens of the *Hypsiboas calcaratus* species complex. Coloration corresponds to preserved specimens unless otherwise noted.

Characters	*Hypsiboas alfaroi*	*Hypsiboas almendarizae*	*Hypsiboas calcaratus*	*Hypsiboas fasciatus*	*Hypsiboas maculateralis*	*Hypsiboas tetete*
Flank Coloration	Creamy white or gray with dark brown irregular spots	Pale cream or creamy white with thin dark brown vertical bars	Pale cream, creamy white or light gray with dark brown vertical bars	Pale cream, creamy white, brown or gray with dark brown vertical bars	Pale cream, creamy white or gray with dark brown blotches	Creamy white or gray with dark brown irregular spots
Coloration of the hidden surfaces of thighs	Creamy white, gray or brown with dark brown irregular spots	Pale cream or creamy white with thin dark brown transversal bars	Pale cream, creamy white or light gray with dark brown transversal bars	Pale cream, creamy white, brown or gray with dark brown transversal bars	Pale cream, creamy white or gray with dark brown blotches	Creamy white or brown with dark brown irregular spots
Size and shape of the calcar	Calcar absent. Only small tubercle present	Large and conical	Large and triangular	Small and conical	Large and triangular	Calcar absent. Only small tubercle present
Brown flecks on the neck and chest	Present	Absent	Absent	Absent	Sometimes present	Present
Iris upper band, in life	Ill-defined, yellow	Well-defined, yellow to orange	Well-defined, yellow to orange	Well-defined, red to reddish brown	Ill-defined, yellow	Ill-defined, yellow

#### Variation.

Variation in dorsal and ventral coloration of preserved specimens is shown in Figure 9. Background dorsal coloration varies from cream (e.g., QCAZ 40085) to pinkish white (e.g., QCAZ 44530), reddish brown (e.g., QCAZ 14957, 43256, 44422), pale reddish brown (e.g., QCAZ 43259) or pale grayish brown (e.g., QCAZ 48718). Irregular dorsal marks may be present in diverse patterns. A dark middorsal line extends from the tip of the snout to the mid-sacrum (e.g., QCAZ 43256), but in some specimens it only extends along the head (e.g., QCAZ 25514) or on the anterior half of the body (e.g., QCAZ 43131). There is variation in the number, size, and shape of dorsal marks. Some individuals (e.g., QCAZ 43256) have five to seven brown diffuse transversal bands (sometimes interconnected). Brown transversal bars are present on the dorsal surfaces of the limbs (one or two on the upper arm and forearm and three to five on the thigh, shank, and foot). In some individuals, the dorsum and dorsal surfaces of the forearms and shanks have large black stains (e.g., QCAZ 14957) or scattered brown or white dots (e.g., QCAZ 40085, 44178, 14971). The coloration of flanks and hidden surfaces of thighs vary from pale cream to creamy white or light gray, with dark brown transversal bars. The number of bars on the flank varies from 4 to 13; the number of bars on the thigh varies from 4 to 9. The extent of the area with bars varies from the groin to the mid flank (e.g., QCAZ 43259) to from the groin to the axilla (e.g., QCAZ 43256). In some individuals, the bars can also be present on the hidden surfaces of the shanks, ventral surfaces of the forelimbs, and dorsal surfaces of the feet (e.g., QCAZ 43256).

Ventral surfaces of preserved specimens vary from creamy white (e.g., QCAZ 44530) to yellowish white (e.g., QCAZ 43256). In some individuals, scattered minute pale brown blotches are present on the lips (e.g., QCAZ 31446, 44178). Coloration of webbing and discs vary from yellowish white to brown or gray. Coloration of bones is white or green.

#### Coloration in life.

(based on photographs; Figs 4C and 8C). Dorsal surfaces vary from light brown (e.g., QCAZ 40056) to reddish brown (e.g., QCAZ 36869) or brown (e.g., QCAZ 24282) with a middorsal dark brown line (e.g., QCAZ 40985); some individuals have brown diffuse transversal bands (e.g., QCAZ 43256); the dorsal surfaces of the limbs have pale brown transversal bars (e.g., QCAZ 43256); scattered minute white and black dots can be present on the dorsum (e.g., QCAZ 40056); in some individuals there are large dark brown blotches on the dorsum, dorsal surfaces of the forearms and shanks (e.g., QCAZ 43245); flanks are white, light blue or blue with dark brown vertical bars (e.g., QCAZ 40083); hidden surfaces of thighs and shanks are white, light blue or blue with dark brown transversal bars (e.g., QCAZ 43034); in some specimens there are dark brown transversal bars on the hidden surfaces of the shanks, ventral surfaces of the upper arms, and dorsal surfaces of the feet (e.g., QCAZ 43034); a faint creamy white stripe usually is evident on the outer edge of the feet, tarsus, forearms, and hands (e.g., QCAZ 26062); venter creamy white with belly yellowish white; ventral surfaces of hindlimbs and forelimbs translucent white (e.g., QCAZ 43824) or yellowish (e.g., QCAZ 40085); in some individuals, ventral surfaces of the thighs are creamy white (e.g., QCAZ 43047); discs and webbing yellowish (e.g., QCAZ 40085) or brown (e.g., QCAZ 40985); iris creamy silver (e.g., QCAZ 40056) or bronze (e.g., QCAZ 40085) with an upper yellow to orange band (e.g., QCAZ 43047); bones are white (e.g., QCAZ 40083) or green (e.g., QCAZ 43824).

In the examined adult series, the largest male has a SVL of 42.50 mm, and the largest female 56.29 mm; mean male SVL = 37.08 mm (*n* = 35; SD = 2.09), mean female SVL = 50.92 mm (*n* = 4; SD = 4.80). Females are significantly larger than males (*t* = -5.71, df = 3, *P* = 0.009). Inter-population variation in size and other morphometric variables is shown in Tables 1 and 2.

#### Advertisement call.

Two males were recorded at Tena (Provincia Napo) on 1 March 2009 and five males at Estación Científica Yasuní PUCE (Provincia Orellana) on 20 June 2009, in vegetation next to streams or ponds. Acoustic parameters of the advertisement call are shown in Table 7. The call (Fig. 12E–F) consists of a single quack note with a mean duration of 0.05 s (SD = 0.00) and mean rise time of 0.04 s (SD = 0.01). The mean dominant frequency is 1780.50 Hz (SD = 112.73) and the mean fundamental frequency is 557.13 Hz (SD = 46.21).

**Table 7.** Descriptive statistics for call parameters of *Hypsiboas alfaroi* (QCAZ 43260–63), *Hypsiboas almendarizae* (QCAZ 39645, 39647–50), *Hypsiboas calcaratus* (QCAZ 40084–85, 43247, 43256–59), *Hypsiboas fasciatus* (QCAZ 48583–86, 48633), *Hypsiboas maculateralis* (QCAZ 40082), and *Hypsiboas tetete* (QCAZ 40060, 40080–81, 48095). The *n* values indicate the number of males analyzed. Mean ± SD is given with range in parentheses. Values for *Hypsiboas maculateralis* were obtained from three calls from a single male. See Table 3 for a description of each parameter.

	*Hypsiboas alfaroi* (*n* = 4)	*Hypsiboas almendarizi* (*n* = 5)	*Hypsiboas calcaratus* (*n* = 7)	*Hypsiboas fasciatus* (*n* = 5)	*Hypsiboas maculateralis* (*n* = 1)	*Hypsiboas tetete* (*n* = 4)	
						Type 1	Type 2
Call Duration (s)	0.20 ± 0.05 (0.16–0.19)	0.48 ± 0.07 (0.39–0.55)	0.05 ± 0.00 (0.05–0.06)	0.52 ± 0.24 (0.27–0.90)	0.35 ± 0.04 (0.317–0.398)	0.10 ± 0.02 (0.08–0.12)	0.11 ± 0.02 (0.09–0.13)
Number of notes	4–5	3–4	1	3–5	3–4	1	1
Rise time (s)	0.07 ± 0.03 (0.05–0.08)	0.30 ± 0.10 (0.20–0.45)	0.04 ± 0.01 (0.03–0.05)	0.24 ± 0.06 (0.17–0.30)	0.19 ± 0.10 (0.084–0.289)	0.03 ± 0.02 (0.02–0.06)	0.05 ± 0.02 (0.03–0.07)
Call dominant frequency (Hz)	2079.53 ± 83.43 (1924.50–2032.74)	1954.43 ± 128.43 (1828.18–2115.65)	1780.50 ± 112.73 (1619.32–1927.25)	1855.81 ± 148.08 (1712.00–2088.70)	2217.93 ± 56.94 (2174.92–2282.51)	1938.47 ± 26.24 (1830.31–1959.23)	1829.12 ± 12.61 (1815.97–1841.10)
Call fundamental frequency (Hz)	2036.31 ± 130.30 (1951.05–2146.50)	951.76 ± 61.38 (882.86–1029.95)	557.13 ± 46.21 (512.60–651.38)	884.89 ± 105.52 (722.60–1001.30)	488.10 ± 12.47 (473.71–495.33)	1940.14 ± 28.29 (1830.31–1964.23)	1821.96 ± 16.96 (1808.80–1841.10)
Number of pulses	–	14.85 ± 3.03 (11.90–19.17)	14.12 ± 0.74 (12.60–14.75)	11.80 ± 1.69 (9.60–13.56)	–	–	10.22 ± 1.68 (8.67–12.00)
Pulse repetition rate (pulses/s)	–	200.41 ± 31.89 (168.95–249.22)	268.46 ± 11.08 (250.71–280.11)	179.53 ± 18.08 (150.31–195.95)	–	–	92.97 ± 2.26 (90.72–95.24)
Dominant frequency at the beginning to the third note (Hz)	–	1869.09 ± 52.61 (1808.80–1905.70)	–	1827.51 ± 175.70 (1722.70–2140.10)	–	–	–
Fundamental frequency at the beginning to the third note (Hz)	–	826.16 ± 58.21 (766.58–882.90)	–	841.64 ± 91.67 (728.30–969.00)	–	–	–
Dominant frequency at the end to the third note (Hz)	–	2058.93 ± 33.98 (2026.28–2094.10)	–	1962.32 ± 190.41 (1798.80–2282.50)	–	–	–
Fundamental frequency at the end to the third note (Hz)	–	937.43 ± 83.53 (882.90–1033.60)	–	950.18 ± 144.22 (709.50–1055.10	–	–	–
Interval between calls (s)	7.75 ± 0.95 (7–9)	16.2 ± 6.37 (10–15)	12 ± 2.64 (6–23)	51 ± 24.72 (13–78)	152.5 ± 3.53 (150–155)	9 ± 4.24 (6–15)	–
Recording temperatures (°C)	25.9	21.5	23.9–25.6	20.1–23.6	21.5	22.2–25	22.2–25

#### Distribution and ecology.

*Hypsiboas calcaratus* has confirmed records (based on DNA sequences and specimens listed in Appendix) from French Guiana, Guyana and the Amazon basin of Brazil, Ecuador, and Peru (Fig. 17). A photograph published by De la Riva et al. (2000) confirms its presence in Bolivia. Records from Colombia and Venezuela need confirmation. Known localities range in elevation from sea level (Kaw) to 650 m (Canelos).

*Hypsiboas calcaratus* occurs in *Terra Firme* forest, flooded forests (*Várzea* and *Igapó*), and swamps. It is generally found next to streams, ponds, and lakes. Individuals have been recorded at night perching on vegetation 15 to 200 cm above the ground. Their occurrence in secondary forests and artificial open areas suggest at least some tolerance of anthropogenic habitat disturbance.

Vegetation types at known localities include Southwest Amazon Moist Forest and Napo Moist Forest for the Peruvian and Ecuadorian localities, Guianan Moist Forest for the Guyana and French Guiana localities, and Madeira-Tapajós Moist Forest for the Brazilian locality (according to the World Wildlife Fund, 2012).

#### Conservation status.

Its distribution polygon has 3’586,597 km^2^ and overlaps with protected areas and large regions of pristine forest. *Hypsiboas calcaratus* is relatively frequent in scientific collections suggesting that, at least in part of its range, it is not a rare species. For these reasons we propose assigning *Hypsiboas calcaratus* to the Red List category of Least Concern.

### Hypsiboas fasciatus

(Günther, 1858)

http://species-id.net/wiki/Hypsiboas_fasciatus

Hyla fasciata Gunther, 1858: 327. Holotype: BMNH 58.4.25.22, a female from “Anden von Ecuador” (Andes of Ecuador).

#### Diagnosis.

A member of the genus *Hypsiboas* characterized by: (1) mean SVL 35.40 mm in males (range 32.65–37.74; *n* = 19), 51.89 mm in females (range 47.16–54.84; *n* = 5); (2) basal webbing on fingers; (3) calcar small and conical; (4) dorsal coloration varying from cream to grayish brown, pinkish white or brown, with dark marks in some individuals (e.g., broad transversal bands); (5) middorsal brown stripe often present; (6) flanks pale cream or gray (in life, blue in large females and light blue or white in males) with dark brown vertical bars; (7) hidden surfaces of thighs pale cream or gray (in life, blue in large females and light blue or white in males) with dark brown transversal bars; (8) ventral surfaces of thighs creamy white, yellowish white or brown; (9) venter creamy white or yellowish white; (10) webbing on feet; (11) in life, iris creamy silver or bronze with upper red to reddish brown band; (12) prepollical spine present in males.

**Figure 14.**
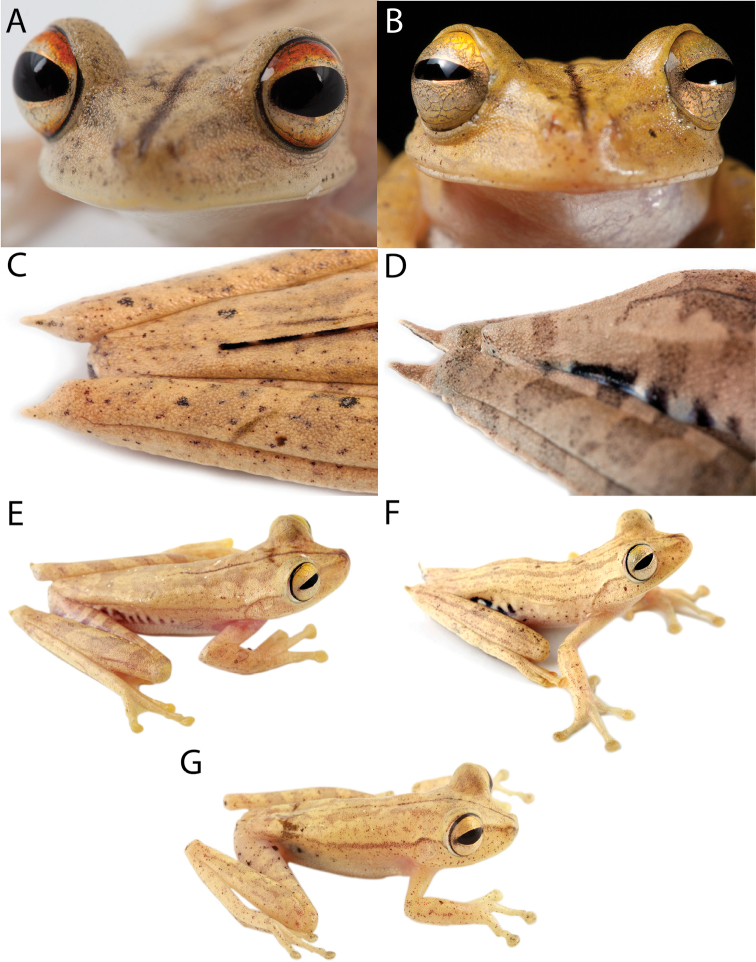
Variation in color of the iris of **A** *Hypsiboas fasciatus* (QCAZ 47051), and **B** *Hypsiboas almendarizae* (QCAZ 42055). Calcar in **C** *Hypsiboas almendarizae* (QCAZ 39639) and **D** *Hypsiboas calcaratus* (QCAZ 40055). Note that the calcar in *Hypsiboas almendarizae* is large and conical while in *Hypsiboas calcaratus* is large and triangular. Dorsolateral views of the holotypes of **E** *Hypsiboas almendarizae*, adult male (QCAZ 39650), SVL = 36.72 mm **F** *Hypsiboas maculateralis*, adult male (QCAZ 40082), SVL = 36.16 mm; and **G** *Hypsiboas tetete*, adult male (QCAZ 40081), SVL = 31.15 mm.

*Hypsiboas fasciatus* is most similar to *Hypsiboas almendarizae* sp. n. and *Hypsiboas calcaratus*. *Hypsiboas calcaratus* can be distinguished from *Hypsiboas fasciatus* by the shape of the calcar (large and triangular in *Hypsiboas calcaratus* vs. small and conical in *Hypsiboas fasciatus*), structure of the advertisement calls (Fig. 12A–B and 12E–F), and by the yellow to orange color of the upper band in the iris (red to reddish brown in *Hypsiboas fasciatus*). *Hypsiboas almendarizae* sp. n. differs from *Hypsiboas fasciatus* in having narrower transversal dark bars on the flanks and thighs (mean width of bars on thighs = 5.05% of femur length, SD = 0.61, in *Hypsiboas almendarizae* sp. n.vs. 8.58%, SD = 1.56, in *Hypsiboas fasciatus*). The color of the upper band in the iris also distinguishes both species (red to reddish brown in *Hypsiboas fasciatus* vs. yellow to orange in *Hypsiboas almendarizae* sp. n.; Fig. 14A–B).

*Hypsiboas fasciatus* differs from *Hypsiboas maculateralis* sp. n. in advertisement call (Figs 12A–B and 13A–B) and in the presence of transversal bars on the flanks and hidden surfaces of the thighs (dark blotches instead of bars in *Hypsiboas maculateralis* sp. n.) *Hypsiboas fasciatus* can easily be distinguished from *Hypsiboas alfaroi* sp. n. and *Hypsiboas tetete* sp. n. by the presence of a calcar (instead of a small tubercle on the heel) and by the absence of dark flecks on the gular region and chest (present in *Hypsiboas alfaroi* sp. n. and *Hypsiboas tetete* sp. n.) Morphological characters useful to differentiate *Hypsiboas fasciatus* from other species are shown in Table 6.

#### Variation.

Variation in dorsal and ventral coloration of preserved specimens is shown in Figure 5. Background dorsal coloration varies from cream (e.g., QCAZ 41488, 48584) to pale grayish brown (e.g., QCAZ 18271), grayish brown (e.g., QCAZ 27258), pinkish white (e.g., QCAZ 41575), pale brown (e.g., QCAZ 23148) or brown (e.g., QCAZ 26497). Irregular dorsal marks can be present in diverse patterns. A dark middorsal stripe extends from the tip of the snout to the mid-sacrum (e.g., QCAZ 26497) or to the vent (QCAZ 23144). In a few individuals, the middorsal line is absent (QCAZ 17123, 31040). In some individuals (e.g., QCAZ 41575) broad transversal bands are present on the dorsum; narrower brown transversal bars are present on the dorsal surfaces of the limbs (one or two on the upper arm and forearm and three to five on the thigh, shank, and foot). In some individuals, scattered minute brown dots may be present on dorsum (e.g., QCAZ 48584). The coloration of flanks and hidden surfaces of thighs vary from pale cream to creamy white, brown or gray, with dark brown vertical bars (4 to 12 on each flank and 6 to 12 on each thigh). The area with vertical bars extends from the groin to the axilla (e.g., QCAZ 41575) or to the mid flank (e.g., QCAZ 41576, 18271). In some individuals, similar transversal bars can be present on the hidden surfaces of the shanks, ventral surfaces of the forelimbs, and dorsal surfaces of the feet (e.g., QCAZ 41575). The shape of the calcar is small and conical and shows little variation among individuals.

Ventral areas vary from creamy white (e.g., QCAZ 48584) to yellowish white (e.g., QCAZ 41576, 41488). A narrow to wide brown stripe can be present on the outer edge of the hands, forearms, thighs, feet, and tarsal folds (e.g., QCAZ 48584, 41576, 41575, 26497). In some specimens, scattered minute pale brown blotches can be present on the lips (e.g., QCAZ 26497, 24866). Coloration of webbing and discs vary from yellowish white to brown or gray. Bones are white.

In the examined series of adults, the largest male has a SVL of 37.74 mm, and the largest female 54.84 mm; mean male SVL = 35.40 mm (*n* = 19; SD = 1.65), mean female SVL = 51.89 mm (*n* = 5; SD = 3.18). Females are significantly larger than males (*t* = -16.24, df = 22, *P* < 0.001). Inter-population variation in size and other morphometric variables is shown in Tables 1 and 2.

#### Coloration in life.

(based on digital photographs; Figs 4A and 8A). Dorsal surfaces vary from pale yellowish tan (e.g., QCAZ 47070) to brown (e.g., QCAZ 48611) with a middorsal dark brown line (e.g., QCAZ 48585) and scattered minute black dots (e.g., QCAZ 47051); some individuals have diffuse broad pale brown transversal bands on the dorsum (e.g., QCAZ 48583); pale brown transversal bars are present on the dorsal surfaces of the limbs; flanks and hidden surfaces of thighs are white, light blue or blue with dark brown vertical bars (e.g., QCAZ 47070, 47051); in some specimens, dark brown vertical bars are present on the hidden surfaces of the shanks, ventral surfaces of the upper arms and dorsal surfaces of the feet (e.g., QCAZ 48671); venter creamy white, sometimes with yellowish white on its posterior half (e.g., QCAZ 48670); ventral surfaces of hindlimbs and forelimbs translucent pinkish white (e.g., QCAZ 47051) or brown (e.g., QCAZ 48611); in some specimens, ventral surfaces of the thighs creamy white (e.g., QCAZ 47070); discs and webbing pale yellowish tan (e.g., QCAZ 48584) or brown (e.g., QCAZ 48611); a narrow to wide brown stripe is present on the outer edge of the hands, forearms, thighs, feet, and tarsal folds (e.g., QCAZ 48611); iris creamy silver (e.g., QCAZ 48584) or bronze (e.g., QCAZ 48611) with upper red to reddish brown band (e.g., QCAZ 48628); bones vary from green (e.g., QCAZ 48671) to white (e.g., QCAZ 48628).

#### Advertisement call.

We recorded calls of one male at Bombuscaro (Provincia Zamora Chinchipe) on 9 June 2010, three males at La Pradera (Provincia Morona Santiago) on 10 June 2010, and one male at Comunidad San Luis (Provincia Morona Santiago) on 13 June 2010. The advertisement call consists of three to five quack notes (Fig. 12A–B). Mean call duration is 0.52 s (SD = 0.24) and mean rise time is 0.24 s (SD = 0.06). Sound frequency increases from the beginning to the end of the note; mean dominant frequency is 1855.81 Hz (SD = 148.08) and mean fundamental frequency is 884.89 Hz (SD = 105.52). Other call parameters are listed in Table 7.

#### Distribution and ecology.

**Figure 15.**
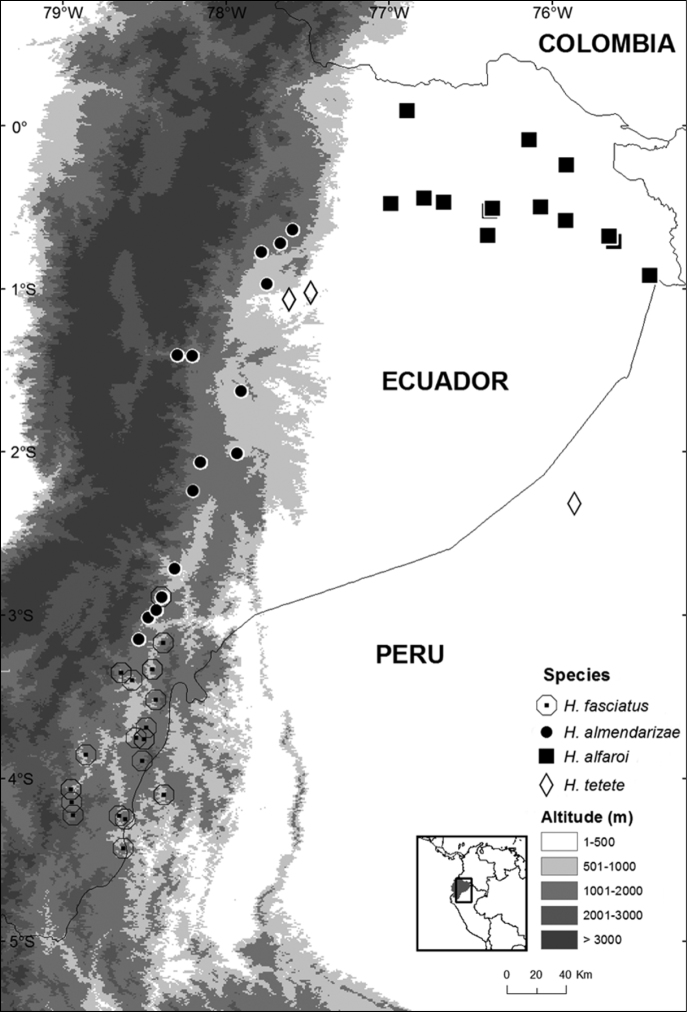
Distribution of species of the *Hypsiboas calcaratus* complex. Localities are based on museum specimens from Museo de Historia Natural Gustavo Orcés of Escuela Politécnica Nacional, Museo de Zoología of Pontificia Universidad Católica del Ecuador, Natural History Museum University of Kansas, and Smithsonian Institution National Museum of Natural History.

*Hypsiboas fasciatus* has been recorded in the Ecuadorian and Peruvian Amazon basin (Morona Santiago and Zamora-Chinchipe provinces in Ecuador and Región Amazonas in Peru) (Fig. 15). Localities with known elevation range from 730 to 1593 m above sea level. The elevation at Romerillos Alto (1593 m) is the highest known locality for *Hypsiboas fasciatus*, while Tink (730 m) is the lowest.

Specimens from Comunidad San Luis, La Pradera, La Pituca, Limón, Miazi Alto, Nueva Principal, and Tiink were found in primary and secondary forest, perching on vegetation 30 to 200 cm above the ground, in flooded areas, ponds, swamps, and near streams. Individuals in Zamora were found in grassy swamps roosting in shrubs between 30 and 110 cm above ground.

Vegetation types for Ecuadorian localities are: (1) Amazonian Evergreen Foothill Forest, characterized by a mixture of Amazonian and Andean vegetation with a canopy of 30 m, (2) Evergreen Lower Montane Forest of the Amazonian Range, characterized by trees reaching 20–30 m of height and abundant epiphytes and hemiepiphytes, with *Dictyocaryum lamarckianum* (Arecaceae) as dominant species, and (3) Evergreen Lower Montane Forest of the East of the Southern Andes, characterized by abundant epiphytes, trees reaching 30 m of height with *Podocarpus* as dominant species.

#### Conservation Status.

Its distribution polygon has 8,572 km^2^ of which 2,198 km^2^ (25.6%) have been degraded by human activities, especially agriculture and cattle raising (estimated from Ministerio de Ambiente del Ecuador 2013). Because habitat degradation is increasing, we assign *Hypsiboas fasciatus* to the Red List category Near Threatened. Its distribution polygon overlaps with three protected areas: Parque Nacional Podocarpus, Reserva Biológica el Quimi and Refugio de Vida Silvestre el Zarza. Its distribution polygon does not overlap with the polygon reported in the Red List assessment for this species by Icochea et al. (2004). The new range represents < 1% of the range reported in the IUCN Red List.

### Hypsiboas almendarizae sp. n.

http://zoobank.org/5D68CEA5-D2D1-44A9-AA7C-D30081D92C7D

http://species-id.net/wiki/Hypsiboas_almendarizae

#### Common name.

English: Almendariz’s treefrog; Spanish: Rana arbórea de Almendáriz

#### Holotype.

(Fig. 14E) QCAZ 39650 (field no. SC-PUCE 23213), adult male from Ecuador, Provincia Morona Santiago, General Leonidas Plaza, “Limón”, on the road to Gualaceo (2.9796°S, 78.4415°W), 1237 m above sea level, collected by Marcel A. Caminer on 17 January 2009.

#### Paratopotypes.

(Fig. 8B) QCAZ 39638–40, 39645–49, adult males, collected with the holotype.

#### Paratypes.

(Figs 4B, 6) ECUADOR: PROVINCIA NAPO: Pacto Sumaco (0.6339°S, 77.5922°W), 1560 m, QCAZ 10910, adult male, collected by L. E. López on 1 January 1996; Río Hollín (0.6998°S, 77.6665°W), 1068–1950 m, QCAZ 22365–66, adult males, collected by M. R. Bustamante on 27 January 2002; Río Hollín, on the road Jondachi-Loreto (0.7707°S, 77.7820°W), 1100 m, QCAZ 6905, 6910, adult males, 6889, juvenile, collected by P. Ordoñez, P. Guarderas, J. F. Freile, M. C. Terán and O. Torres-Carvajal on 3 December 1994; Río Hollín, on the road Hollín-Loreto (0.9666°S, 77.7632°W), 600 m, QCAZ 283, 782, 4177–78, adult males, collected by A. Flachier, F. Campos-Yánez, L. A. Coloma and G. Onore on 5 December 1987 and 1 February 1988; Río Hollín (0.6998°S, 77.6665°W), 1068–1950 m, QCAZ 21942, 22364, adult males, 17944, collected by M. R. Bustamante, I. G. Tapia, G. Onore, F. Ayala-Varela, S. Valdivieso, D. Tirira and J. J. Wiens on 20 March 1990 and 9 December 2001; Archidona, Estacíon Sarayacu (0.6910°S, 77.8208°W), 1320 m, QCAZ 51809, juvenile female, collected by D. Rivadeneira and X. Salazar on 23 August 2011; PROVINCIA TUNGURAHUA: Baños, Río Lagarto (1.4025°S, 78.2980°W), 1472 m, QCAZ 24392, 24394, adult males, collected by I. G. Tapia and D. Hill on 5 May 2001; Río Verde (1.4026°S, 78.2979°W), 1514 m, QCAZ 47047–48, adult males, collected by S. Poe, F. Ayala-Varela, L. Gray, J. Davis and I. Latella on 14 December 2009; Río Negro (1.4135°S, 78.2110°W), 1220 m, QCAZ 4029–30, 4907, 21273–74, adult males, 24357, adult female, 4034, metamorph, collected by F. Campos-Yánez, L. A. Coloma, C. Proaño and J. J. Camacho on 1 May 1993 and 24 September 2002; mouth of the Pastaza river (1.4128°S, 78.2688°W), 1440 m, QCAZ 31449, adult male, 31450, adult female, collected by D. Paucar on 9 March 2006; Baños, Río Lagarto (1.4025°S, 78.2980°W), 1472 m, QCAZ 24386–91, 24393, 24395, adult males, collected by I. G. Tapia and D. Hill on 5 May 2001; mouth of the Pastaza river (1.4128°S, 78.2687°W), 1440 m, QCAZ 31452, juvenile, collected by D. Paucar on 8 March 2006; PROVINCIA PASTAZA: Pomona, Reserva de Bosque Tropical Hola Vida (1.6250°S, 77.9072°W), 831 m, QCAZ 37163, adult male, collected by I. G. Tapia, L. A. Coloma, C. Proaño and M. Páez on 9 July 2007; PROVINCIA MORONA SANTIAGO: 2.2 km N San Juan Bosco (2.0070°S, 77.9348°W), 1013 m, QCAZ 26438, adult male, collected by L. A. Coloma on 12 August 2003; Parque Nacional Sangay, Sardinayacu river (2.0597°S, 78.1564°W), 1600 m, EPN 7740, adult male, collected by A. Almendáriz; Central Hidroeléctrica Abanico (2.2457°S, 78.1958°W), 1600 m, EPN 11435–36, 11438, adult males, 11437, adult female, collected by Y. Sagredo and J. Brito; 7.6 km W from Nueve de Octubre, along the road Guamote-Macas (2.2163°S, 78.2898°W), 1571 m, QCAZ 32638–39, 32645, adult females, collected by A. Pounds, L. A. Coloma, M. R. Bustamante and I. G. Tapia on 30 November 2006; 4.8 km N from Rosario (2.8858°S, 78.3880°W), 841 m, QCAZ 26474–77, adult males, 26480, metamorph, collected by L. A. Coloma, A. Merino and E. R. Wild on 13 August 2003; N from Mendez, 4 km NW from Patuca (airline distance) on the road to Logroño (2.7291°S, 78.2849°W), 600 m, QCAZ 23810, adult male, collected by S. R. Ron and G. E. Romero on 6 April 2003; El Rosario (2.9282°S, 78.4082°W), 1100 m, QCAZ 30590–92, adult males, collected by I. G. Tapia, M. R. Bustamante and A. Merino on 23 March 2004; N from General Leonidas Plaza, “Limón”, on the road Limón-Méndez (2.9046°S, 78.3869°W), 879 m, QCAZ 26300, adult male, collected by S. R. Ron, M. Guerra and I. G. Tapia on 13 January 2004; Limón Indanza, on the road to Ayanza (2.9899°S, 78.4260°W), 994 m, QCAZ 39642, adult male, collected by M. A. Caminer on 17 January 2009; near General Leonidas Plaza, “Limón”, km 74 on the road to San Antonio (2.9266°S, 78.4070°W), 1070 m, QCAZ 8573, adult male, collected by N. Acosta-Buenaño and J. Bosch on 16 July 1995.

#### Referred specimens.

ECUADOR: PROVINCIA MORONA SANTIAGO: 2.2 km N San Juan Bosco (2.0070°S, 77.9348°W), 1013 m (QCAZ 26429–37, 26439); Nueve de Octubre, on the road Guamote-Macas (2.2445°S, 78.2074°W), 1527 m (QCAZ 32268, 37639); Méndez (2.7145°S, 78.3153°W), 500 m (QCAZ 1006–07); Km 65 on the road Macas-Méndez (2.68083°S, 78.23341°W), 540 m (QCAZ 15904–06, 15999); 13 km N from Limón (2.8895°S, 78.3952°W), 800 m (QCAZ 27317–23, 27325); Limón Indanza (2.9796°S, 78.4414°W), 800–1560 m (QCAZ 8572, 25958, 26407, 26555, 26562, 31866, 32831, 32834, 32836, 32842, 39642, 40027, 41932–33, 41992, 42001, 42017–18, 42022, 42026, 42037, 42039–40, 42055, 42236); Plan de Milagro-Limón, 4.3 km WSW Limón (3.0118°S, 78.4784°W), 1373–1560 m (QCAZ 26376–85, 26400–01, 41894, 41897, 41898); 3 km Limón-Indanza, on the road to Gualaquiza (3.0489°S, 78.5007°W), 950 m (QCAZ 48176–78); on the road Indanza-Gualaquiza (3.1435°S, 78.5359°W), 1277 m (QCAZ 26421–22); Bosque Protector Abanico, 1646–1720 m (QCAZ 49029, 49034).

#### Diagnosis.

A member of the genus *Hypsiboas* characterized by: (1) mean SVL 37.64 mm in males (range 34.31–44.56; *n* = 23), 48.11 mm in females (range 37.80–51.94; *n* = 4); (2) basal webbing on fingers; (3) calcar large, conical; (4) dorsal coloration varying from cream to reddish brown, grayish brown or pale brown, sometimes with dark marks (e.g., broad transversal bands or narrow longitudinal lines); (5) dark brown middorsal line often present; (6) flanks pale cream or creamy white (in life, blue in large females and light blue or white in males) with thin dark brown vertical bars; (7) hidden surfaces of thighs pale cream or creamy white (in life, blue in large females and light blue or white in males) with thin dark brown transversal bars; (8) ventral surfaces of thighs creamy white or yellowish white; (9) venter creamy white or yellowish white; (10) webbing on feet; (11) in life, iris cream or creamy silver with an upper yellow to orange band; (12) prepollical spine present in males.

*Hypsiboas almendarizae* is most similar to *Hypsiboas fasciatus* and *Hypsiboas calcaratus*. It can be distinguished from *Hypsiboas calcaratus* by the shape of the calcar (large and conical in *Hypsiboas almendarizae* vs. large and triangular in *Hypsiboas calcaratus*; Fig. 14C–D), width of the transversal lines on the flanks (thin in *Hypsiboas almendarizae* vs. wide in *Hypsiboas calcaratus*), and advertisement call (Fig. 12C–F). *Hypsiboas almendarizae* differs from *Hypsiboas fasciatus* in having narrower transversal dark bars on the flanks and thighs (mean width of bars on thighs = 5.05% of femur length in *Hypsiboas almendarizae* vs. 8.58% in *Hypsiboas fasciatus*; differences are significant: *t* = 7.05, df = 12, *P* < 0.001) and in color of the upper band of the iris (red to reddish brown in *Hypsiboas fasciatus* vs. yellow to orange in *Hypsiboas almendarizae*; Fig. 14A–B).

*Hypsiboas almendarizae* differs from *Hypsiboas maculateralis* sp. n. in advertisement call (Figs 12C–D and 13A–B) and in the presence of vertical lines on the flanks and hidden surfaces of the thighs (dark blotches instead of lines in *Hypsiboas maculateralis* sp. n.) *Hypsiboas almendarizae* can be distinguished from *Hypsiboas alfaroi* sp. n. and *Hypsiboas tetete* sp. n. by the presence of a calcar (instead of a small tubercle on the heel) and by the absence of dark flecks on the gular region and chest (present in *Hypsiboas alfaroi* sp. n. and *Hypsiboas tetete* sp. n.) Morphological characters useful to differentiate *Hypsiboas almendarizae* from other species are shown in Table 6.

#### Description of the holotype.

Adult male, 36.72 mm SVL, foot length 15.93 mm, head length 9.18 mm, head width 11.95 mm, eye diameter 4.26 mm, tympanum diameter 2.41 mm, tibia length 21.78 mm, femur length 20.03 mm, calcar length 1.10 mm, arm length 6.17 mm, eye-nostril distance 2.7 mm, head wider than long and wider than body; snout round in lateral view, truncate in dorsal view; distance from nostril to eye shorter than diameter of eye; canthus rostralis indistinct, rounded; loreal region concave; internarial area convex; nostrils not protuberant, directed laterally; interorbital area slightly convex; eye large, strongly protuberant; diameter of eye 1.8 times diameter of tympanic annulus; tympanum concealed beneath skin;tympanic annulus evident, ovoid, longer dorsoventrally, concealed dorsally by supratympanic fold, separated from eye by ca. 1.2 times its diameter; posterior end of supratympanic fold reaches anterior border of arm insertion. Arm slender, axillary membrane absent; indistinct low tubercles present along ventrolateral edge of forearm; relative length of fingers I<II<IV<III; fingers bearing large, oval discs, that of third finger about three fourths of tympanum diameter; subarticular tubercles prominent, round to ovoid, single; supernumerary tubercles present; palmar tubercle small, elongated; prepollical tubercle large, flat, elliptical; prepollex enlarged, claw shaped; nuptial excrescences absent; webbing absent between fingers. Large conical calcar on tibiotarsal articulation; ill defined, scattered tubercles on tarsus and along ventrolateral edge of foot; toes bearing discs slightly wider than long, smaller than those of fingers; relative length of toes I<II<V<III<IV; outer metatarsal tubercle ill defined, small, round; inner metatarsal tubercle large, elliptical; subarticular tubercles single, round, flat; supernumerary tubercles restricted to the soles; webbing formula of toes I2—2¾II1¾—3^-^III2^-^—3^+^IV3^+^—2^-^V. Skin on dorsum, head, and dorsal surfaces of limbs smooth; skin on flanks smooth with weak longitudinal wrinkles posterior to the arm; skin on venter coarsely granular; skin on ventral surfaces of head and thighs granular, those of shanks smooth. Cloacal opening directed posteriorly at upper level of thighs; cloacal sheath short simple, covering cloacal opening; round tubercles below and on the sides of vent, larger proximally. Tongue cordiform, widely attached to mouth floor; vomerine odontophores triangular, narrowly separated, posteromedial to choanae, bearing six vomerine teeth on each side; choanae trapezoidal, oblique.

*Color of holotype in preservative*. Dorsum pale grayish brown with five to seven broad brown transversal bands and scattered minute black dots; few small cream dots on the posterior one third of the dorsum; dark brown middorsal line from tip of the snout to the mid-dorsum; brown transversal bars on dorsal surfaces of the limbs (two on the upper arm, five on the thigh, and three on the shank and foot); flanks pale cream with ten thin dark brown transversal bars; hidden surfaces of thighs pale cream with seven to nine thin dark brown transversal bars; narrow cream stripe present above the anus; venter yellowish white becoming creamy white on its posterior half; ventral surfaces of hindlimbs, forelimbs, and webbing yellowish white; faint narrow to wide pale brown stripe on the outer edge of the hands and forearms; minute brown blotches on lips; bones white.

*Color of holotype in life*. (Fig. 14E). Dorsum pale yellow tan with pale broad brown transversal marks and scattered minute black and white dots; dark brown middorsal line is present; pale brown transversal bars on the dorsal surfaces of the limbs; flanks white with faint thin dark brown vertical bars; hidden surfaces of thighs white with thin dark brown transversal bars; venter creamy white; ventral surfaces of hindlimbs and forelimbs translucent pinkish; discs and webbing yellowish; iris cream with upper yellow band; bones white.

#### Etymology.

The specific name is a noun in the genitive case and is a patronym for Ana Almendáriz, Ecuadorian herpetologist who for more than three decades has contributed to the study of Ecuadorian amphibians and reptiles. Ana Almendáriz is curator of Herpetology in the Museo de Historia Natural Gustavo Orcés at Escuela Politécnica Nacional del Ecuador.

#### Variation.

Variation in dorsal and ventral coloration of preserved specimens is shown in Figure 6. Background dorsal coloration varies from creamy white (e.g., QCAZ 32638, 32645) to reddish brown, grayish brown (e.g., QCAZ 24394, 39647), pale grayish brown (e.g., QCAZ 24392, 39650) or pale brown (e.g., QCAZ 24386). A dark middorsal line extends from the tip of the snout to the middle of the sacrum (e.g., QCAZ 39647) or to the vent (e.g., QCAZ 32645), but in some specimens is restricted to the head (e.g., QCAZ 26429) or to the anterior half of the body (e.g., QCAZ 39650). In few specimens, the middorsal line is faint or absent (e.g., QCAZ 24392). Irregular dorsal marks may be present in varying number, size, and shape. Five to seven broad transversal bands (sometimes interconnected) can be present on the dorsum. Some individuals (e.g., QCAZ 24394, 39650, 39647) have narrow brown transversal bars on the dorsal surfaces of the limbs (one or two on the upper arm and forearm, and three to five on the thigh, shank, and foot). Faint to well-defined narrow longitudinal lines may be present on the dorsum (e.g., QCAZ 32638, 32645). In some individuals (e.g., QCAZ 32645), there are few scattered brown or white dots on the dorsum, and dorsal surfaces of forearms, shanks and tarsi. The coloration of the flanks and hidden surfaces of the thighs vary from pale cream to creamy white, with thin dark brown transversal bars. The number of bars on the flanks and thighs varies from six to ten on each flank and six to thirteen on each thigh; the area with vertical bars extends from the groin to the mid-flank. In few individuals, the dark transversal bars of the thighs and flanks are faint (e.g., QCAZ 32645).

Ventral surfaces of preserved specimens vary from creamy white (e.g., QCAZ 39650) to yellowish white (e.g., QCAZ 39647). A narrow to wide faint pale brown stripe is present in some individuals on the outer edge of the hands, forearms, feet, thighs, and tarsal folds (e.g., QCAZ 39647). In some individuals, scattered minute pale brown blotches may be present on the lips (e.g., QCAZ 32638, 39647). Coloration of webbing and discs vary from yellowish white to brown or gray. Vomerine odontophores are triangular (with arched base in some specimens). Bones white.

In the adult type series, the largest male has a SVL 44.56 mm, and the largest female 51.94 mm; mean male SVL = 37.64 mm (*n* = 23; SD = 2.01), mean female SVL = 48.11 mm (*n* = 4; SD = 6.88). Females were not significantly larger than males (*t* = -3.02, df = 3, *P* = 0.055). Inter-population variation in size and other morphometric variables is shown in Tables 1 and 2.

#### Coloration in life.

(based on photographs; Figs 4B and 8B). Dorsal surfaces vary from pale yellowish tan (e.g., QCAZ 39640) to reddish brown (e.g., QCAZ 32638) with a middorsal dark brown line (e.g., QCAZ 42055); sometimes broad pale brown transversal bands are present on the dorsum; the dorsal surfaces of the limbs often have pale brown transversal bars (e.g., QCAZ 39640); in some specimens, the dorsum has faint to well-defined narrow brown longitudinal lines (e.g., QCAZ 39649); scattered minute black, yellow or white dots can be present on the dorsum (e.g., QCAZ 39648); flanks and hidden surfaces of thighs white, light blue or blue with thin dark brown vertical bars (e.g., QCAZ 39646, 42055); in some individuals there are thin dark brown transversal bars on the hidden surfaces of the shanks (e.g., QCAZ 42055); venter creamy white with yellowish white belly (e.g., QCAZ 48177); ventral surfaces of hindlimbs and forelimbs translucent pinkish (e.g., QCAZ 39650) or yellowish (e.g., QCAZ 39638); in some specimens, ventral surfaces of the thighs creamy white (e.g., QCAZ 49029); discs and webbing yellowish (e.g., QCAZ 48177); iris cream (e.g., QCAZ 39648) or creamy silver (e.g., QCAZ 39639) with upper yellow to orange band (e.g., QCAZ 39646); bones white (e.g., QCAZ 48177).

#### Advertisement call.

We recorded calls from five males at Limón Indanza (Provincia Morona Santiago) on 17 January 2009 (Table 7). The advertisement call consists of three to four quack notes (Fig. 12C–D). Mean call duration is 0.48 s (SD = 0.007) and mean rise time is 0.30 s (SD = 0.10). Sound frequency increases from the beginning to the end of the note; mean dominant frequency is 1954.43 Hz (SD = 128.43) and mean fundamental frequency is 951.76 Hz (SD = 61.38). The advertisement calls of *Hypsiboas almendarizae* and *Hypsiboas fasciatus* are similar to each other (Fig. 12A–D). The distribution ranges of these species are parapatric with a small area of sympatry in the surroundings of Leonidas Plaza (Provincia Morona Santiago). The analyzed calls from *Hypsiboas fasciatus* were from a region of allopatry. Future studies should aim to compare calls from the region of sympatry to explore the possibility of reproductive character displacement.

#### Distribution and ecology.

*Hypsiboas almendarizae* occurs on the eastern Andean slopes of central and southern Ecuador (Morona Santiago, Napo, and Tungurahua provinces) (Fig. 15). Localities with known elevation range from 500 to 1950 m above sea level. The elevation at Río Hollín (1950 m) is the highest known locality for *Hypsiboas almendarizae*, while Méndez (500 m) is the lowest.

Most specimens of *Hypsiboas almendarizae* were collected at Río Napinaza, a river surrounded by secondary forest, pastures, and agricultural lands. Frogs were perching on vegetation 25 to 80 cm above the ground. Individuals from Limón Indanza, Río Hollín, Río Lagarto, Méndez, Río Pastaza, and Nueve de Octubre were found in flooded areas with pastures, on vegetation 30 to 70 cm above the ground. Few individuals were found near river Hollín, in small ponds in primary and secondary forest, roosting on branches and leaves 20 to 150 cm above the ground. All the specimens from Rosario and Plan de Milagro were found next to a highway, in swamps and streams with grass.

Vegetation types are: (1) Amazonian Evergreen Foothill Forest, characterized by a mixture of Amazonian and Andean vegetation with a canopy of 30 m, (2) Evergreen Lower Montane Forest of the Amazonian Range, characterized by trees reaching 20–30 m of height and abundant epiphytes and hemiepiphytes, with dense vegetation, and *Dictyocaryum lamarckianum* (Arecaceae) as dominant species, (3) Evergreen Lower Montane Forest of the East of the Southern Andes, characterized by abundant epiphytes, trees reaching 30 m of height with *Podocarpus* trees as dominant species, (4) Evergreen Lower Montane Forest of the East of the Northern and Central Andes, characterized by a canopy of 25 to 30 m, with abundant epiphytes and by the absence of species of trees characteristic of the lowlands like the family Bombacaceae and Myristicaceae, and (5) Amazonian Lowland Evergreen Forest, characterized by high plant alpha-diversity and a canopy of 30 m with emergent trees that reach 40 m.

#### Conservation status.

Its distribution polygon has 14,983 km^2^. Within this area, 4,864 km^2^ (32.4%) of its habitat has been degraded by human activities, especially agriculture and cattle raising (estimated from Ministerio de Ambiente del Ecuador 2013). Current habitat degradation within the range of *Hypsiboas almendarizae* is extensive and its increase may threaten its survival in the future. Therefore, we propose that *Hypsiboas almendarizae* is assigned to the Near Threatened category. Its distribution polygon overlaps with Parque Nacional Sangay, Parque Nacional Llanganates, and Parque Nacional Sumaco Napo-Galeras.

### Hypsiboas maculateralis sp. n.

http://zoobank.org/BB94092B-695D-4747-ADFD-313747D7FAE1

http://species-id.net/wiki/Hypsiboas_maculateralis

#### Common name.

English: Stained treefrog; Spanish: Rana arbórea manchada

#### Holotype.

(Fig. 14F) QCAZ 40082 (field no. SC-PUCE 23221), adult male from Ecuador, Provincia Napo, Comunidad Santa Rosa (1.02337°S, 77.48359°W), 354 m above sea level, collected by Marcel A. Caminer and Edwin Carrillo Ponce on 28 February 2009.

#### Paratypes.

ECUADOR: PROVINCIA ORELLANA: Río Napo, Santa Teresita, 4 km NW from Nuevo Rocafuerte (0.9008°S, 75.4135°W), 186 m, QCAZ 44651, adult female, 44673, adult male, collected by S. R. Ron, E. Toral, and I. G. Tapia on 9 July 2009; Río Napo, Huiririma (0.7116°S, 75.6239°W), 194 m, QCAZ 44636, adult male, collected by S. R. Ron, E. Toral and I. G. Tapia on 8 July 2009; Río Napo, San Vicente (0.6790°S, 75.6511°W), 203 m, QCAZ 44531, adult female, 44532, adult male, collected by S. R. Ron, E. Toral and I. G. Tapia on 7 July 2009; Río Napo, Chiroisla (0.5756°S, 75.8998°W), 203 m, QCAZ 44452, adult male, collected by S. R. Ron, E. Toral and I. G. Tapia on 5 July 2009; Río Napo, Edén (0.4983°S, 76.0711°W), 216 m, QCAZ 44182–83, 44186, 44188–90, adult males, collected by S. R. Ron, E. Toral and I. G. Tapia on 4 July 2009; Río Napo, sector La Primavera (0.4442°S, 76.7868°W), 244 m, QCAZ 43825, adult female, 43827, adult male, QCAZ 43800, juvenile, collected by S. R. Ron, E. Toral and I. G. Tapia on 29 June 2009; Río Napo, El Descanso (0.4310°S, 76.7864°W), 244 m, QCAZ 43897, adult male, collected by S. R. Ron, E. Toral and I. G. Tapia on 30 June 2009; El Coca (0.4778°S, 76.9898°W), 267 m, QCAZ 43712, adult male, 43709–10, 43715, juveniles, collected by S. R. Ron, E. Toral and I. G. Tapia on 27 June 2009; Guiyero community (0.6248°S, 76.4944°W), 227 m, EPN 10319, adult male, collected by A. Almendáriz and J. Hernández; Guiyero community, Nambai km 32 (0.6248°S, 76.4944°W), 240 m, EPN 10887, adult male, collected by A. Almendáriz, J. Awa and P. Ima; Parque Nacional Yasuní, km 74 on the road Pompeya-Iro (0.8331°S, 76.3416°W), 257 m, QCAZ 5238, adult female, collected by S. de la Torre and S. R. Ron on 20 February 1994; Nuevo Rocafuerte, Tambococha (0.9783°S, 75.4256°W), 177 m, QCAZ 55359, adult male, 55395, 55374, adult females, collected by F. Ayala-Varela, E. Carillo, J. Brito, A. Varela and D. Quirola on 13 March 2013; PROVINCIA Sucumbíos: Zábalo, Familia Criollo (0.3181°S, 75.7662°W), 220 m, QCAZ 28004, 28024, adult females, collected by M. R. Bustamante, N. Acosta-Buenaño, M. Guerra and C. Proaño on 30 September 2003 and 2 October 2003; Playas de Cuyabeno (0.2654°S, 75.8917°W), 230 m, QCAZ 28401, adult female, collected by M. R. Bustamante, N. Acosta-Buenaño, M. Guerra and C. Proaño on 8 October 2003; Playas de Cuyabeno, Agurico Pañacoha (0.3106°S, 76.0335°W), 200 m, EPN 13452, adult male, collected by P. Meza Ramos; Putumayo, Güepi camp 20 km N of Zábalo (0.1779°S, 75.6767°W), 220 m, EPN 7189, adult female, collected by A. Almendáriz; Reserva Faunística Cuyabeno (0.0849°N, 76.1344°W), 273 m, QCAZ 2148, 29454, adult males, 2156, adult female, collected by S. de la Torre and F. Campos on 13 May 1990 and 9 May 2004; Río Napo, La Selva Lodge (0.5086°S, 76.3649°W), 229 m, QCAZ 44020–21, adult males, collected by S. R. Ron, E. Toral and I. G. Tapia on 2 July 2009; Indillana, (0.43370°S, 76.5419°W), 265 m, QCAZ 24672, subadult, collected by F. Ayala-Valera on 11 January 2002; Campo Vinita, on the road Palma Roja-Pto El Carmen de Putumayo (0.1220°N, 75.8611°W), 217 m, QCAZ 29280–82, adult males, collected by S. Aldás-Alarcón on 7 May 2004; PROVINCIA PASTAZA: Bataburo Lodge, S of the road from Cononaco (1.2083°S, 76.7166°W), 250 m, QCAZ 39430, 39452, adult males, collected by S. D. Padilla and C. Meyer on 10 October 2008 and 13 October 2008.

#### Referred specimens.

ECUADOR: PROVINCIA SUCUMBÍOS: Reserva de Producción Faunística Cuyabeno, Estación Científica Universidad Católica del Ecuador, Laguna Grande (0.0195°S, 76.1712°W), 220 m (QCAZ 6141–43, 6146–51); PERU: REGION MADRE DE DIOS: Manu National Park, Cocha Cashu Biological Station, Río Manu (11.9166°S, 71.3°W), 340 m (USNM 299774).

#### Diagnosis.

A member of the genus *Hypsiboas* characterized by: (1) mean SVL 36.00 mm in males (range 31.86–39.17; *n* = 22), 45.18 mm in females (range 32.04–55.31; *n* = 6); (2) basal webbing on fingers; (3) calcar large, triangular; (4) dorsal coloration varying from creamy white to reddish brown, pinkish white or brown, sometimes with dark markings (e.g., narrow longitudinal lines, broad transversal marks, or large black stains); (5) dark brown middorsal line restricted to the head or the anterior half of the body often present; (6) flanks pale cream or creamy white (in life, blue in large females and light blue or white in males) with dark brown blotches; (7) hidden surfaces of thighs pale cream or gray (in life, blue in large females and light blue or white in males) with dark brown blotches; (8) ventral surfaces of thighs yellowish white or brown; (9) ventral coloration varying from creamy white to yellowish white with or without brown flecks on the neck and chest; (10) webbing on feet; (11) in life, iris cream silver, yellowish or cream with faint yellow to orange color on its upper quarter; (12) prepollical spine present in males.

**Figure 16.**
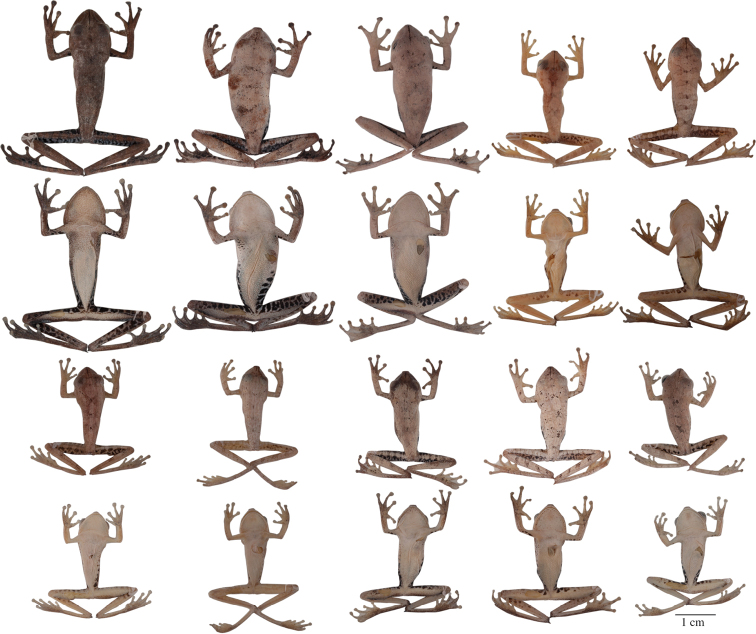
Adult *Hypsiboas maculateralis* showing variation in dorsal and ventral coloration of preserved specimens. From left to right, first and second rows: QCAZ 43825, 44531, 44651, 28004, 28401 (females); third and fourth rows: QCAZ 40082 (holotype), 43897, 44020–21, 44184 (males). See Appendix for locality data. All specimens are shown at the same scale.

*Hypsiboas maculateralis* (Figs 4D, 8D and 16) differs from *Hypsiboas fasciatus*, *Hypsiboas almendarizae*, and *Hypsiboas calcaratus* in advertisement call (Figs 12 and 13A–B) and by having dark blotches on the flanks and thighs (dark lines in *Hypsiboas almendarizae*, *Hypsiboas calcaratus*, and *Hypsiboas fasciatus*). *Hypsiboas maculateralis* can be distinguished from *Hypsiboas alfaroi* sp. n. and *Hypsiboas tetete* sp. n. by the presence of a calcar (instead of a small tubercle on the heel). Morphological characters useful to differentiate *Hypsiboas maculateralis* from other species are shown in Table 6.

#### Description of holotype.

Adult male, 36.16 mm SVL, foot length 14.33 mm, head length 8.98 mm, head width 11.71 mm, eye diameter 4.07 mm, tympanum diameter 2.44 mm, tibia length 20.29 mm, femur length 18.44 mm, calcar length 1.44 mm, arm length 6.36 mm, eye-nostril distance 2.86 mm, head wider than long and wider than body; snout rounded in lateral view, truncate in dorsal view; distance from nostril to eye shorter than diameter of eye; canthus rostralis indistinct, rounded; loreal region concave; internarial area convex; nostrils not protuberant, directed laterally; interorbital area slightly convex; eye large, strongly protuberant; diameter of eye 1.7 times diameter of tympanic annulus; tympanum concealed beneath skin;tympanic annulus evident, ovoid, longer dorsoventrally and concealed dorsally by supratympanic fold, separated from eye by ca. 1.1 times its diameter; posterior end of supratympanic fold reaches anterior border of arm insertion. Arm slender, axillary membrane absent; indistinct low tubercles along ventrolateral edge of forearm; relative length of fingers I<II<IV<III; fingers bearing large, oval discs, that of third finger about three fourths of tympanum diameter; subarticular tubercles prominent, round to ovoid, single; supernumerary tubercles present; palmar tubercle small, elongated; prepollical tubercle large, flat, elliptical; prepollex enlarged, claw shaped; nuptial excrescences absent; webbing absent between fingers. Large triangular calcar on tibiotarsal articulation; ill defined, scattered tubercles on tarsus and along ventrolateral edge of foot; toes bearing discs slightly wider than long, smaller than those of fingers; relative length of toes I<II<V<III<IV; outer metatarsal tubercle ill defined, small, round; inner metatarsal tubercle large, elliptical; subarticular tubercles single, round, flat; supernumerary tubercles restricted to the soles; webbing formula of toes I2—2^+^II1¾—3^-^III2^-^—3IV3—1½V. Skin on dorsum, head, and dorsal surfaces of limbs smooth; skin on flanks smooth with weak longitudinal wrinkles posterior to the arm; skin on venter coarsely granular; skin on ventral surfaces of head and thighs granular, those of shanks smooth. Cloacal opening directed posteriorly at upper level of thighs; cloacal sheath short and simple, covering cloacal opening; round tubercles below and on sides of vent, larger proximally. Tongue ovoid, widely attached to mouth floor; vomerine odontophores triangular with arched base, narrowly separated, posteromedial to choanae, each bearing 8–12 teeth; choanae ovoid.

*Color of holotype in preservative*. Dorsum pale reddish brown with two faint brown narrow longitudinal lines interconnected in the scapular region (Fig. 16); scattered minute black dots distributed on dorsum; dark brown middorsal line along snout; dorsal surfaces of limbs pale reddish brown with five pale brown narrow transversal bars on each thigh; flanks and hidden surfaces of thighs pale cream with dark brown blotches; narrow white stripe above vent; venter creamy white; ventral surfaces of limbs and webbing yellowish white; narrow to wide pale brown stripe on the outer edge of the hands, forearms, thighs, tarsal folds, and feet; minute brown blotches on lips; bones white.

*Color of holotype in life*. (Fig. 14F). Dorsum creamy white with six narrow pale brown longitudinal lines; a dark brown middorsal line; five pale brown faint transversal bars on dorsal surface of each thigh and two narrow pale brown longitudinal lines on dorsal surface of each shank; scattered minute black dots on dorsum; flanks and hidden surfaces of the thighs light-bluish white with dark brown blotches; discs and webbing pale yellowish tan; iris cream with faint yellow coloration on its upper margin.

#### Etymology.

The specific name is derived from the Latin words *macula* = stain, and *lateralis* = lateral, in reference to the brown dark blotches on the flanks of these frogs.

#### Variation.

Variation in dorsal and ventral coloration of preserved specimens is shown in Figure 16. Background dorsal coloration varies from creamy white (e.g., QCAZ 44020) to reddish brown (e.g., QCAZ 28401), pale reddish brown (e.g., QCAZ 28004), pale pinkish white (e.g., QCAZ 44021, 43897), pinkish white (e.g., QCAZ 44531, 44651, 44184) or brown (e.g., QCAZ 43825). A dark middorsal line extends from the tip of the snout to the middle of dorsum (e.g., QCAZ 2156), but in some specimens is restricted to the snout (e.g., QCAZ 43825, 44651, 28004) or is absent (e.g., QCAZ 44531). There is variation in the number, size, and shape of the dorsal marks. Five to seven broad transversal bands on the dorsum (sometimes interconnected) and narrower brown transversal bars on the dorsal surfaces of the limbs (one or two each on upper arm and forearm and three to five each on thigh, shank and foot) may be present (e.g., QCAZ 28401). In some individuals, faint, narrow longitudinal dark lines may be present (e.g., QCAZ 43897). In a few specimens, the dorsum, dorsal surfaces of forearms, and shanks may present large black stains (e.g., QCAZ 44021) and/or few scattered brown dots (e.g., QCAZ 43897, 44020). The coloration of the flanks and hidden surfaces of thighs vary from pale cream to creamy white or gray, with dark brown blotches. There is variation in the distribution of blotches on flanks; the extent covered by the blotches ranges from groin to the mid-flank (e.g., QCAZ 28004) or to the axilla (e.g., QCAZ 55374). In some individuals, similar blotches can also be present on the hidden surfaces of the shanks, ventral surfaces of the forelimbs, and dorsal surfaces of the feet (e.g., QCAZ 43825). In few individuals, the dark blotches of the thighs and flanks are faint (e.g., QCAZ 43897).

Ventral surfaces of preserved specimens vary from creamy white (e.g., QCAZ 44020, 44184; Fig. 16) to yellowish white (e.g., QCAZ 28004). Some individuals have brown flecks on the gular region, chest, and lips (e.g., QCAZ 43825). A narrow to wide brown stripe is present in some individuals on the outer edge of the hands, forearms, thighs, tarsal folds and feet (e.g., QCAZ 28004, 28401, 44021). Coloration of webbing and disc varies from yellowish white to brown or gray. Vomerine odontophores are triangular (with arched base in some specimens). Coloration of bones is white.

In the adult type series, the largest male has a SVL of 39.17 mm, and the largest female 55.31 mm; mean male SVL = 36 mm (*n* = 22; SD = 1.92), mean female SVL = 45.18 mm (*n* = 6; SD = 9.39). Females are significantly larger than males (*t* = -3.43, df = 4, *P* = 0.025). Inter-population variation in size and other morphometric variables are shown in Tables 1 and 2.

#### Coloration in life.

(based on photographs; Figs 4D and 8D). Dorsal surfaces vary from pale yellowish tan (e.g., QCAZ 43827) to yellowish cream (e.g., QCAZ 44636) with a middorsal dark brown line (e.g., QCAZ 43827); sometimes, ill-defined broad pale brown transversal bands (e.g., QCAZ 43827) or narrow pale brown longitudinal lines are present on the dorsum (e.g., QCAZ 44636); in some individuals, there are pale brown transversal bars on the dorsal surfaces of the limbs (e.g., QCAZ 44636); scattered minute black dots may be present on the dorsum (e.g., QCAZ 44636); flanks white, light blue or blue with dark brown blotches (e.g., QCAZ 43825, 43827, 44636); hidden surfaces of thighs white, gray, orange, light blue or blue with dark brown blotches (e.g., QCAZ 43825, 43825, 44636); in some specimens, hidden surfaces of the shanks, ventral surfaces of the upper arms and dorsal surfaces of the feet are blue with dark brown blotches (e.g., QCAZ 43825); venter creamy, sometimes with yellowish on its anterior half and with brown flecks on the neck, chest, and lips (e.g., QCAZ 43827); ventral surfaces of hindlimbs and forelimbs translucent white (e.g., QCAZ 43827) or translucent orange (e.g., QCAZ 44636); in some specimens, ventral surfaces of the thighs creamy white (e.g., QCAZ 43825); discs and webbing brown (e.g., QCAZ 43825), orange (e.g., QCAZ 44636) or pale yellowish tan (e.g., QCAZ 43827); a narrow to wide brown stripe may be present on the outer edge of the hands, forearms, thighs, feet, and tarsal folds (e.g., QCAZ 43825, 43827); iris yellowish (e.g., QCAZ 43715) or cream with faint yellow to orange color on its upper quarter (e.g., QCAZ 43825); bones vary from white to green (e.g., QCAZ 43827, 44636). Juveniles have similar coloration to adults, except that the blotches on the flanks and hidden surfaces of thighs are absent (e.g., QCAZ 43709–10, 43715, 43800).

#### Advertisement call.

A call from a male (QCAZ 40082) at Comunidad Santa Rosa (Napo Provincia) recorded on 28 February 2009 (Table 7) consists of three to four notes that sound like a high-pitched chuckle. The mean call duration is 0.35 s (SD = 0.04) and the mean rise time is 0.19s (SD = 0.1). The dominant frequency is higher (mean = 2217.93 Hz, SD = 56.94) and the fundamental frequency lower (mean = 488.10 Hz, SD = 12.47) compared to *Hypsiboas fasciatus*, *Hypsiboas almendarizae*, *Hypsiboas calcaratus*, *Hypsiboas alfaroi* sp. n. and *Hypsiboas tetete* sp. n.

#### Distribution and ecology.

**Figure 17.**
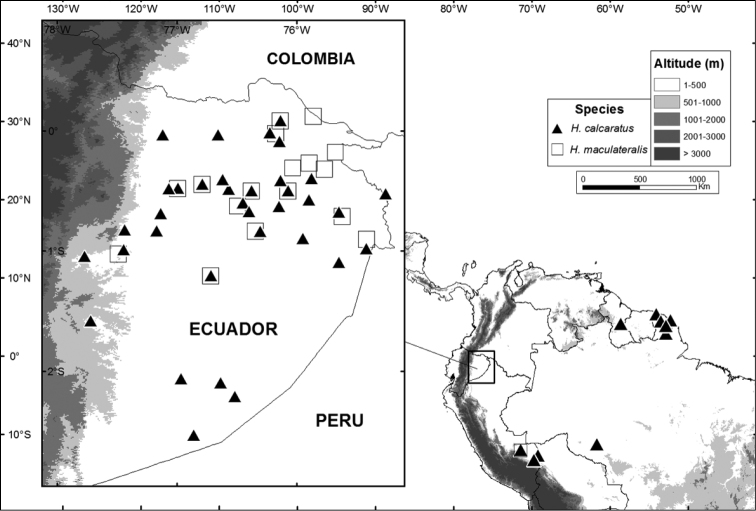
Distribution of *Hypsiboas calcaratus* and *Hypsiboas maculateralis*. Localities are based on museum specimens from Museo de Historia Natural Gustavo Orcés of Escuela Politécnica Nacional, Museo de Zoología of Pontificia Universidad Católica del Ecuador, Natural History Museum University of Kansas, and Smithsonian Institution National Museum of Natural History.

*Hypsiboas maculateralis* inhabits the Amazon basin of Ecuador (Napo, Orellana, Pastaza, and Sucumbíos provinces) and Peru (Región de Madre de Dios) (Fig. 17). Localities with known elevation range vary between 186 and 354 m of elevation. The elevation of Comunidad Santa Rosa (354 m) is the highest known and Santa Teresita (186 m) is the lowest.

All the specimens in Zábalo, Reserva Cuyabeno, Santa Teresita, Bataburo Lodge, and km 74 Pompeya-Iro road were found at night, in primary or secondary forests, perching on vegetation between 40 and 200 cm above the ground, in areas of pasture and flooded forest.

Vegetation types for Ecuadorian localities are: (1) Amazonian Lowland Evergreen Forest, characterized by high plant alpha-diversity and a canopy of 30 m with emergent trees that reach 40 m, (2) Floodplain Lowland Forest of White-Waters, characterized by periodical flooding with white-waters from large rivers, the vegetation reaches 35 m of height, and there are several horizontal strata of vegetation, and (3) Lowland Forest of Palms and black-waters, swamps characterized by a canopy of 30 m with dens e understory and a dominance of the palm *Mauritia flexuosa*.

Vegetation type of the Peruvian locality is Southwest Amazon Moist Forest.

#### Conservation status.

The distribution polygon of *Hypsiboas maculateralis* has an area of 209,304 km^2^. Because its distribution range is large and includes extensive areas of undisturbed forest, we propose that *Hypsiboas maculateralis* be assigned to the category Least Concern.

### Hypsiboas alfaroi sp. n.

http://zoobank.org/6D92050D-8BC3-44FD-A749-5B50DD8D2F73

http://species-id.net/wiki/Hypsiboas_alfaroi

#### Common name.

English: Alfaro’s treefrog; Spanish: Rana arbórea de Alfaro

#### Holotype.

QCAZ 43262 (field no. SC-PUCE 23245), adult male from Ecuador, Provincia Orellana, Parque Nacional Yasuní, 1 km from Northern Production Facilities (NPF) oil camp (0.6893°S, 76.4290°W), 238 m above sea level, collected by Marcel A. Caminer and Edwin Carrillo Ponce on 20 June 2009.

#### Paratopotypes.

QCAZ 43260–61, 43263, adult males, collected with the holotype.

#### Paratypes.

ECUADOR: PROVINCIA ORELLANA: Río Napo, Nuevo Rocafuerte, (0.9192°S, 75.4010°W), 187 m, QCAZ 44788–89, 44790–91, adult males, collected by S. R. Ron, E. Toral and I. G. Tapia on 10 July 2009; Nuevo Rocafuerte, Alta Florencia (0.8966°S, 75.4370°W), 187 m, QCAZ 39510, adult male, collected by S. D. Padilla and P. Vargas Mina on 29 October 2008; Río Napo, Huiririma (0.7116°S, 75.6239°W), 194 m, QCAZ 44634–35, adult males, collected by S. R. Ron, E. Toral and I. G. Tapia on 8 July 2009; Río Napo, San Vicente (0.6790°S, 75.6511°W), 203 m, QCAZ 44527, adult male, 44528, adult female, collected by S. R. Ron, E. Toral and I. G. Tapia on 7 July 2009; Río Napo, Chiroisla (0.5756°S, 75.8998°W), 203 m, QCAZ 44351, adult female, 44425, adult male, collected by S. R. Ron, E. Toral and I. G. Tapia on 5 July 2009; Río Napo, Edén (0.4983°S, 76.0711°W), 216 m, QCAZ 39631, 44180, adult females, 44191, adult male, collected by S. Aldas, S. R. Ron, E. Toral and I. G. Tapia on 21 January 2009 and 4 July 2009; Río Napo, Añangu (0.5249°S, 76.3844°W), 255 m, QCAZ 43977–78, adult males, collected by S. R. Ron, E. Toral and I. G. Tapia on 1 July 2009; Estación Científica Yasuní, Universidad Católica del Ecuador (0.6748°S, 76.3844°W), 231 m, QCAZ 43249–50, 43253, 43255 adult males, 43251–52, 43254, adult females, same collectors as the holotype between 18–19 June 2009; Estación Científica Yasuní, Universidad Católica del Ecuador (0.6748°S, 76.3844°W), 219 m, QCAZ 19328, adult female, collected by I. G. Tapia and G. Carotti on 20 February 2002; Río Napo, sector La Primavera (0.4442°S, 76.7868°W), 244 m, QCAZ 43826, adult male, collected by S. R. Ron, E. Toral and I. G. Tapia on 29 June 2009; Río Napo, sector La Primavera, El Descanso (0.4310°S, 76.7864°W), 244 m, QCAZ 43894, adult female, collected by S. R. Ron, E. Toral and I. G. Tapia on 30 June 2009; El Coca (0.4778°S, 76.9898°W), 267 m, QCAZ 559, adult male, collected by Giovanni Onore on 1 April 1984, QCAZ 43682, adult male, 43683–84, adult males, collected by S. R. Ron, E. Toral and I. G. Tapia and 27 June 2009; PROVINCIA SUCUMBÍOS: Playas del Cuyabeno (0.2654°S, 75.8917°W), 230 m, QCAZ 28272, 28398, adult females, 28278, adult male, collected by M. R. Bustamante, N. Acosta-Buenaño, M. Guerra and C. Proaño between 7–8 October 2003; Puerto Bolívar (0.0886°S, 76.1420°W), 240 m, QCAZ 28240, adult male, collected by P. Menéndez and M. R. Bustamante on 5 August 2003; Río Napo, La Selva Lodge (0.5086°S, 76.3649°W), 229 m, QCAZ 44025, 44027, adult males, collected by S. R. Ron, E. Toral and I. G. Tapia on 2 July 2009; Río Napo, 2.5 km S from Pañacocha (0.4712°S, 76.6667°W), 225 m, QCAZ 44851, 44853, 44858, adult males, 44856, adult female, collected by S. R. Ron, E. Toral and I. G. Tapia on 3 July 2009; 2.5 km N from Nueva Loja (0.0917°N, 76.8901°W), 350 m, QCAZ 33983, adult female, 33984, adult male, collected by S. Valdivieso, D. Tirira, J. Wiens and L. A. Coloma on 17 March 1990; Pañacocha, Moretal Sur (0.2758°S, 75.9352°W), 212 m, QCAZ 50785, juvenile, collected by R. Betancourt on 11 February 2011.

#### Referred specimens.

ECUADOR: PROVINCIA ORELLANA: Río Napo, Nuevo Rocafuerte, (0.9192°S, 75.4010°W), 187 m (QCAZ 44789); Río Napo, Huiririma (0.7116°S, 75.6239°W), 194 m (QCAZ 44632–33); Río Napo, San Vicente (0.6790°S, 75.6511°W), 203 m (QCAZ 44525–26); Río Napo, Chiroisla (0.5756°S, 75.8998°W), 203 m, (QCAZ 44424, 44426–27, 44429); Río Napo, Edén (0.4983°S, 76.0711°W), 216 m, (QCAZ 44181, 44249); Estación Científica Yasuní, Universidad Católica del Ecuador (0.6702°S, 76.4376°W), 231 m (QCAZ 8466, 8469, 11883, 19203, 49153); Río Napo, sector La Primavera, El Descanso (0.4310°S, 76.7864°W), 244 m (QCAZ 43895–96); Parque Nacional Yasuní, Pozo SPF, 250 m (QCAZ 31257–59); Nuevo Rocafuerte, Tambococha (0.9783°S, 75.4256°W), 177 m (QCAZ 55226, 55232, 55296, 55363, 55432–33); PROVINCIA SUCUMBÍOS: Playas de Cuyabeno (0.2654°S, 75.8917°W), 230 m (QCAZ 28383–84, 28391, 28397); Puerto Bolívar (0.0886°S, 76.1420°W), 240 m (QCAZ 27815, 28190, 28211, 28230, 28232, 28310, 28315); Río Napo, entrance to La Selva Lodge (0.5086°S, 76.3649°W), 229 m (QCAZ 44022–24, 44026, 44028); La Selva Lodge, Limoncocha (0.4981°S, 76.3738°W), 245 m (QCAZ 25401, 25408, 25410, 25417); Río Napo, 2.5 km S from Pañacocha (0.4712°S, 76.6667°W), 225 m (QCAZ 44848–50, 44852, 44854–55, 44857); 2.5 km N from Nueva Loja (0.0917°N, 76.8901°W), 350 m (QCAZ 2797).

#### Diagnosis.

**Figure 18.**
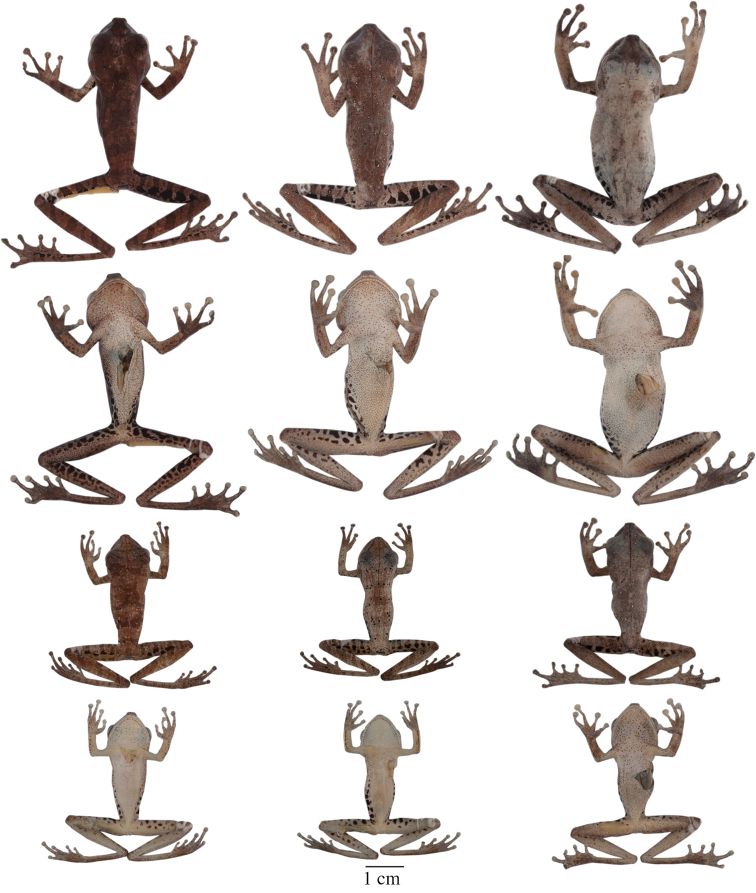
Adult *Hypsiboas alfaroi* showing variation in dorsal and ventral coloration of preserved specimens. From left to right, first and second rows: QCAZ 28272, 28398, 44180 (females); third and fourth rows: QCAZ 43262 (holotype), 43263, 43826 (males). See Appendix for locality data. All specimens are shown at the same scale.

*Hypsiboas alfaroi* (Fig. 4E, 8E and 18) is characterized by: (1) mean SVL 32.80 mm in males (range 27.91–36.27; *n* = 32), 44.51 mm in females (range 39.68–49.21; *n* = 12); (2) basal webbing on fingers; (3) tubercle on heel; (4) dorsal coloration varying from creamy white to grayish brown or brown, sometimes with dark markings (e.g., broad transversal bands, narrow longitudinal lines); (5) faint brown middorsal line often present; (6) flanks creamy white or gray (in life, blue in large females and light blue or white in males) with dark brown dots or blotches; (7) hidden surfaces of thighs creamy white, gray or brown (in life, blue in large females and light blue or white in males) with dark brown dots or blotches; (8) ventral surfaces of thighs creamy white, yellowish white or brown; (9) ventral areas creamy white or yellowish white with brown flecks on the head, neck, and chest; (10) webbing on feet; (11) in life, iris yellowish, bronze or cream with faint yellow coloration on its upper margin; (12) prepollical spine present in males.

*Hypsiboas alfaroi* is most similar to *Hypsiboas tetete* sp. n. but it can be distinguished by having a markedly different advertisement call (Fig. 13C–H). Although with overlapping values, *Hypsiboas alfaroi* has a smaller tympanum (relative to SVL, mean male TD/SVL = 0.06, SD = 0.01, *n* = 32; *Hypsiboas tetete* sp. n. mean male TD/SVL = 0.08, SD = 0.009, *n* = 5; differences are significant: *t* = –3.21, df = 35, *P* = 0.003). *Hypsiboas alfaroi* differs from *Hypsiboas fasciatus*, *Hypsiboas almendarizae*, *Hypsiboas calcaratus*, and *Hypsiboas maculateralis* in advertisement call (Figs 12A–F and 13A–D) and by having a small tubercle on the heel instead of a large calcar. *Hypsiboas alfaroi* further differs from *Hypsiboas fasciatus*, *Hypsiboas almendarizae*, and *Hypsiboas calcaratus* by having dark brown dots on the flanks (vertical dark lines in *Hypsiboas fasciatus*, *Hypsiboas almendarizae*, and *Hypsiboas calcaratus*). Morphological characters useful to differentiate *Hypsiboas alfaroi* from other species are shown in Table 6.

#### Description of holotype.

Adult male, 32.06 mm SVL, foot length 13.22 mm, head length 8.77 mm, head width 10.02 mm, eye diameter 3.69 mm, tympanum diameter 2.19 mm, tibia length 18.74 mm, femur length 16.24 mm, arm length 5.71 mm, eye-nostril distance 2.59 mm, head wider than long and wider than body; snout rounded in lateral view, truncate in dorsal view; distance from nostril to eye shorter than diameter of eye; canthus rostralis indistinct, rounded; loreal region concave; internarial area convex; nostrils not protuberant, directed laterally; interorbital area slightly convex; eye large, strongly protuberant; diameter of eye 1.7 times diameter of tympanic annulus; tympanum concealed beneath skin;tympanic annulus evident, ovoid, longer dorsoventrally and concealed dorsally by supratympanic fold, separated from eye by ca. 1.01 times its diameter; posterior end of supratympanic fold reaches anterior border of arm insertion. Arm slender, axillary membrane absent; indistinct low tubercles present along ventrolateral edge of forearm; relative length of fingers I<II<IV<III; fingers bearing large, oval discs, that of third finger about three fourths of tympanum diameter; subarticular tubercles prominent, round to ovoid, single; supernumerary tubercles present; palmar tubercle small, elongated; prepollical tubercle large, flat, elliptical; prepollex enlarged, claw shaped; nuptial excrescences absent; webbing absent between fingers. Small tubercle on tibiotarsal articulation; ill defined, scattered tubercles on tarsus and along ventrolateral edge of foot; toes bearing discs slightly wider than long, smaller than those of fingers; relative length of toes I<II<V<III<IV; outer metatarsal tubercle ill defined, small, round; inner metatarsal tubercle large, elongated, elliptical; subarticular tubercles single, round, flat; supernumerary tubercles restricted to the soles; webbing formula of toes I2—2II2—3III2—3IV3—1¾V. Skin on dorsum, head, and dorsal surfaces of limbs smooth; skin on flanks smooth with weak longitudinal wrinkles posterior to the arm; skin on venter coarsely granular; skin on ventral surfaces of head and thighs granular, those of shanks smooth. Cloacal opening directed posteriorly at upper level of thighs; short simple cloacal sheath covering cloacal opening; round tubercles below and on the sides of vent. Tongue ovoid, widely attached to mouth floor; vomerine odontophores triangular, narrowly separated, posteromedial to choanae, bearing 10 vomerine teeth on each side; choanae trapezoidal, oblique.

*Color of holotype in preservative*. Dorsum grayish brown with five to six broad diffuse brown transversal bands; scattered minute black dots on dorsal surfaces; few small cream dots restricted to the posterior dorsum; faint brown narrow middorsal line restricted to the head; flanks creamy white with dark brown dots; dorsal surfaces of hindlimbs and forelimbs grayish brown with narrow transversal brown bars (one or two on each upper arm and forearm, and three or four on each thigh, shank, and tarsus); hidden surfaces of thighs grayish brown with dark brown dots; venter creamy white with brown flecks on head, neck, and chest; ventral surfaces of hindlimbs and forelimbs yellowish white with a narrow to wide brown stripe on the outer edge of the hands, forearms, tarsal folds, and feet; bones white.

#### Etymology.

The specific name is a noun in the genitive case and is a patronym for Eloy Alfaro Delgado, former Ecuadorian president (1897–1901 and 1906–1911) and leader of the liberal revolution in Ecuador. His government promoted the separation between church and state and the modernization of Ecuador by supporting education and large-scale systems of transportation and communication.

#### Variation.

Variation in dorsal and ventral coloration of preserved specimens is shown in Figure 18. Background dorsal coloration varies from creamy white (e.g., QCAZ 44429) to pale grayish brown (e.g., QCAZ 19328), grayish brown (e.g., QCAZ 44180), pale brown (e.g., QCAZ 28398) or brown (e.g., QCAZ 28272). Irregular dorsal marks may be present in diverse patterns. A faint dark middorsal line extends from the tip of the snout to the mid-dorsum (e.g., QCAZ 43895) or the sacral region (QCAZ 44025), but in some specimens it is restricted to the head (e.g., QCAZ 55926) or is altogether absent (e.g., QCAZ 28272). There is variation in the number, size, and shape of the dorsal marks. Five to seven diffuse broad transversal bands on the dorsum (sometimes interconnected), and narrower brown transversal bars on the dorsal surfaces of the limbs (one or two each on the arm and forearm, and three to five each on the thigh, shank, and foot) may be present (e.g., QCAZ 28272). Faint, narrow longitudinal lines may be present on the dorsum (e.g., QCAZ 44858, 43263). The dorsum and dorsal surfaces of limbs can have scattered black or white dots (e.g., QCAZ 43263, 28398). The coloration of flanks varies from creamy white to gray with irregular dark brown spots distributed from the groin to the mid-flank. In some individuals, similar spots can also be present in the hidden surfaces of the thighs, shanks, ventral surfaces of the forelimbs, and dorsal surfaces of the feet (e.g., QCAZ 28272). The hidden surfaces of thighs sometimes have dark brown blotches (QCAZ 28272).

Ventral areas of preserved specimens vary from creamy white (e.g., QCAZ 28398, 44180) to yellowish white (e.g., QCAZ 43263) with dark flecks on the head, neck, and chest (Fig. 18). In some individuals, the flecks are also present on hindlimbs, forelimbs, and belly (e.g., QCAZ 28272, 28398). A narrow to wide brown stripe is present in some individuals on the outer edge of the hands, forearms, thighs, tarsal folds, and feet (e.g., QCAZ 43263). Coloration of webbing and disc vary from brown to gray. Vomerine odontophores are triangular (with arched base in some specimens). Bones are white.

In the adult type series, the largest male has a SVL of 36.27 mm, and the largest female 49.21 mm; mean male SVL = 32.80 mm (*n* = 32; SD = 1.97), mean female SVL = 44.51 mm (*n* = 12; SD = 3.09). Females were significantly larger than males (*t* = -14.94, df = 42, *P* < 0.001). Inter-population variation in size and other morphometric variables are shown in Tables 1 and 2.

#### Coloration in life.

(based on photographs; Figs 4E and 8E). Dorsal surfaces vary from pale creamy white (e.g., QCAZ 39631) to yellowish tan (e.g., QCAZ 43261), reddish brown (e.g., QCAZ 43254) or brown (e.g., QCAZ 43978) with a faint brown middorsal line (e.g., QCAZ 43978); sometimes, broad pale brown transversal bands are present on the dorsum (e.g., QCAZ 43978); narrow pale brown transversal bars (e.g., QCAZ 43683) or narrow pale brown longitudinal lines (e.g., QCAZ 44635) may ornament the dorsal surfaces of the limbs; scattered minute black dots on the dorsum may be present (e.g., QCAZ 44635); flanks are white, light blue or blue with irregular dark brown spots (e.g., QCAZ 43683) or blotches (e.g., QCAZ 43252); hidden surfaces of thighs are gray, white, light blue or blue with dark brown irregular spots (e.g., QCAZ 43683) or blotches (e.g., QCAZ 39631); in some specimens, the hidden surfaces of the shanks and dorsal surfaces of the feet have similar brown spots (e.g., QCAZ 43254); venter creamy white (e.g., QCAZ 43254) or yellowish with creamy white belly (e.g., QCAZ 43260); conspicuous brown flecks are present on the ventral areas of the head, neck, and chest (e.g., QCAZ 44635); ventral surfaces of hindlimbs and forelimbs are translucent white (e.g., QCAZ 43683) or yellowish (e.g., QCAZ 43260); in some specimens, the ventral surfaces of the thighs are creamy white (e.g., QCAZ 43254); discs and webbing are brown (e.g., QCAZ 43978), yellowish (e.g., QCAZ 43261) or pale cream (e.g., QCAZ 39631); a narrow to wide brown stripe may be present on the outer edge of the hands, forearms, thighs, feet, and tarsal folds (e.g., QCAZ 43978); iris yellowish (e.g., QCAZ 43683), bronze (e.g., QCAZ 43254) or cream with a subtle yellow tone on its upper quarter (e.g., QCAZ 44635); coloration of bones varies from white (e.g., QCAZ 43252) to green (e.g., QCAZ 43683).

#### Advertisement call.

Three males were recorded at Estación Científica Yasuní PUCE (Provincia Orellana) on 20 June 2009. The call (Fig. 13C–D) consists of four to five trill-like notes with a mean duration of 0.20 s (SD = 0.05) and mean rise time of 0.07 s (SD = 0.03). Other call parameters are listed in Table 7.

#### Distribution and ecology.

*Hypsiboas alfaroi* occurs in the Ecuadorian northern Amazon region (Napo, Orellana, and Sucumbíos provinces; Fig. 15). Localities with known elevation range from 176 m (Nuevo Rocafuerte) to 350 m (Nueva Loja). Nuevo Rocafuerte is on the border between Ecuador and Peru; the occurrence of *Hypsiboas alfaroi* in Peru is highly likely.

Specimens from Nuevo Rocafuerte, Playas de Cuyabeno, Puerto Bolívar, Estación Científica Yasuní of Universidad Católica del Ecuador, and Nueva Loja were found at night in primary and secondary forest, perching on vegetation 50 to 180 cm above the ground, in flooded areas, swamps, near streams and in forest away from water bodies. Individuals at San Vicente were found in pastures, secondary forests, flooded grassland, and ponds.

Vegetation types for Ecuadorian localities are: (1) Amazonian Lowland Evergreen Forest, characterized by high plant alpha-diversity and a canopy of 30 m with emergent trees that reach 40 m, (2) Floodplain Lowland Forest of White Waters, characterized by periodical flooding with white waters from large rivers and vegetation that reaches 35 m of height with several vegetation strata, and (3) Lowland Forest of Palms and black-waters, swamps characterized by a canopy of 30 m with dense understory and a dominance of the palm *Mauritia flexuosa*.

#### Conservation status.

The distribution polygon of *Hypsiboas alfaroi* has 47,524 km^2^. Within it, 4,287 km^2^ (9.0%) have been degraded by human activities, especially agriculture and cattle raising (estimated from Ministerio de Ambiente del Ecuador 2013). Because its distribution range is relatively large and has a low proportion of degraded habitat, we propose that *Hypsiboas alfaroi* be assigned to the Least Concern category.

### Hypsiboas tetete sp. n.

http://zoobank.org/D9448B7C-4778-4912-A21A-3E32E4266EF1

http://species-id.net/wiki/Hypsiboas_tetete

#### Common name.

English: Tetete’s treefrog; Spanish: Rana arbórea de los Tetetes

#### Holotype.

(Fig. 14G) QCAZ 40081 (field no. SC-PUCE 23220), adult male from Ecuador, Provincia Napo, Comunidad Santa Rosa, road to Tena (1.0214°S, 77.4782°W), 344 m above sea level, collected by Marcel A. Caminer and Edwin Carrillo Ponce on 28 February 2009.

#### Paratopotypes.

QCAZ 40060–61, 40079–80, adult males, collected with the holotype.

#### Paratypes.

ECUADOR: PROVINCIA NAPO: Jatun Sacha (1.0649°S, 77.6142°W), 420 m, QCAZ 48094, adult male, 48095–96, adult females, collected by S. R. Ron and Morley Read on 31 March 2010.

#### Referred specimens.

PERU: REGIÓN LORETO: San Jacinto (2.3125°S, 75.8628°W), 180 m (KU 221864).

#### Diagnosis.

**Figure 19.**
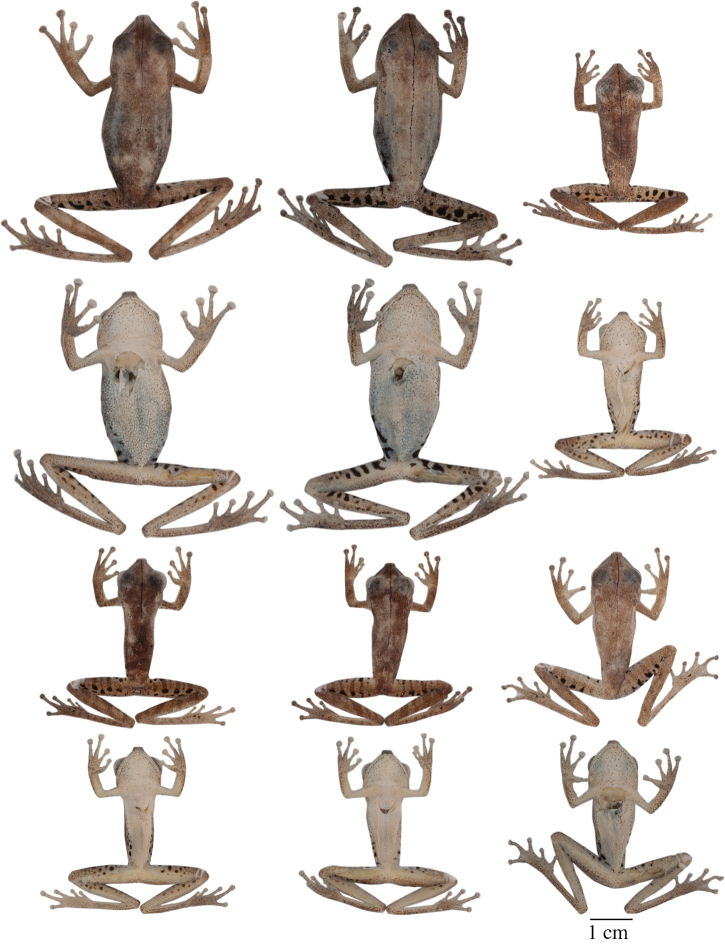
Adult *Hypsiboas tetete* showing variation in dorsal and ventral coloration of preserved specimens. From left to right, first and second rows: QCAZ 48095–96 (females), 40060 (male); third and fourth rows: QCAZ 40079, 40081 (holotype), 48094 (males). See Appendix for locality data. All specimens are shown at the same scale.

*Hypsiboas tetete* (Figs 8F and 19) is characterized by: (1) mean SVL 31.72 mm in males (range 31.15–32.24; *n* = 5), 45.59 mm in females (range 45.33–45.85; *n* = 2); (2) basal webbing on the fingers; (3) tubercle on the heel; (4) dorsal background coloration varying from grayish brown to pale brown, sometimes with dark markings (e.g., diffuse broad transversal bands); (5) faint brown middorsal line often present; (6) flanks creamy white or gray (in life, light blue in large females and white in males) with dark brown irregular spots; (7) hidden surfaces of thighs creamy white or brown with dark brown irregular spots or blotches; (8) ventral surfaces of thighs creamy white or yellowish white; (9) ventral areas creamy white or yellowish white with brown flecks on the head, neck, and chest; (10) webbing on the feet; (11) in life, iris yellow or cream with yellow on its anterior half; (12) prepollical spine present in males.

*Hypsiboas tetete* differs from *Hypsiboas fasciatus*, *Hypsiboas almendarizae*, *Hypsiboas calcaratus*, and *Hypsiboas maculateralis* in advertisement call (Figs 12A–F and 13A–H) and by having a tubercle on the heel instead of a calcar. *Hypsiboas tetete* is most similar to *Hypsiboas alfaroi* from which differs in advertisement call (Fig. 13C–H). Although with overlapping values, *Hypsiboas tetete* has a statistically significant larger tympanum than *Hypsiboas alfaroi* (see *Hypsiboas alfaroi* diagnosis). Morphological characters useful to differentiate *Hypsiboas tetete* from other species are shown in Table 6.

#### Description of holotype.

Adult male, SVL 31.15 mm, foot length 12.01 mm, head length 7.48 mm, head width 10.31 mm, eye diameter 3.51 mm, tympanum diameter 2.25 mm, tibia length 17.76 mm, femur length 16.09 mm, arm length 5.50 mm, eye-nostril distance 1.97 mm, head wider than long and wider than body; snout rounded in lateral view, truncate in dorsal view; distance from nostril to eye shorter than diameter of eye; canthus rostralis indistinct, rounded; loreal region concave; internarial area convex; nostrils not protuberant, directed laterally; interorbital area slightly convex; eye large, strongly protuberant; diameter of eye 1.6 times diameter of tympanic annulus; tympanum concealed beneath skin;tympanic annulus evident, ovoid, longer dorsoventrally and concealed dorsally by supratympanic fold, separated from eye by ca. 1.03 times its diameter; posterior end of supratympanic fold reaches anterior border of arm insertion. Arm slender, axillary membrane absent; indistinct low tubercles present along ventrolateral edge of forearm; relative length of fingers I<II<IV<III; fingers bearing large, oval discs, that of third finger about three fourths of tympanum diameter; subarticular tubercles prominent, round to ovoid, single; supernumerary tubercles present; palmar tubercle small, elongated; prepollical tubercle large, flat, elliptical; prepollex enlarged, claw shaped; nuptial excrescences absent; webbing absent between fingers. Small tubercle on tibiotarsal articulation; ill defined, scattered tubercles on tarsus and along ventrolateral edge of foot; toes bearing discs slightly wider than long, smaller than those of fingers; relative length of toes I<II<V<III<IV; outer metatarsal tubercle ill defined, small, round; inner metatarsal tubercle large, elongated and elliptical; subarticular tubercles single, round, flat; supernumerary tubercles restricted to the soles; webbing formula of toes I2^-^—2½II2—3^+^III2—3^+^IV3^+^—2^-^V. Skin on dorsum, head, and dorsal surfaces of limbs smooth; skin on flanks smooth with weak longitudinal wrinkles posterior to the arm; skin on venter coarsely granular; skin on ventral surfaces of head and thighs granular, those of shanks smooth. Cloacal opening directed posteriorly at upper level of thighs; short simple cloacal sheath covering cloacal opening; round tubercles below and on the sides of vent. Tongue ovoid, widely attached to mouth floor; vomerine odontophores triangular with arched base, barely separated, posteromedial to choanae, bearing eight vomerine teeth on each side; choanae ovoid.

*Color of holotype in preservative*. Dorsum grayish brown with scattered minute black dots; faint brown narrow middorsal line extends from the tip of the snout to the mid-sacrum; dorsal surfaces of hindlimbs and forelimbs grayish brown with narrow transversal brown bars (one or two on each forearm and three or four on each thigh, shank, and tarsus); flanks creamy white with dark brown irregular spots; hidden surfaces of thighs grayish brown with dark brown irregular spots; venter creamy white with brown flecks on the neck, chest, and lips; ventral surfaces of hindlimbs and forelimbs yellowish white with a narrow to wide brown stripe on the outer edge of the hands, forearms, thighs, tarsal folds, and feet; bones white.

*Color of holotype in life*. (Fig. 14G). Dorsum pale yellowish tan with four narrow pale brown longitudinal lines; a dark brown middorsal line extends from the tip of snout to mid-sacrum; dorsal surfaces of hindlimbs yellowish tan with pale brown transversal bars; scattered minute dark brown dots on the dorsal surfaces of limbs and dorsum; flanks white with well-defined dark brown irregular spots; hidden surfaces of thighs pale yellowish tan with dark brown spots; venter creamy white with yellowish white belly; ventrally, scattered brown flecks on the chest, gular region, and jaw margin; ventral surfaces of hindlimbs and forelimbs creamy white; discs and webbing pale yellow tan; iris cream with faint yellow coloration on its upper half; bones white.

#### Etymology.

The specific name is a noun and refers to the Tetete, a Western Tucanoan indigenous group that inhabited the Colombian and Ecuadorian Amazon. It was decimated by the rubber exploitation and became extinct during the 1970s (Wasserstrom et al. 2011). Its recent disappearance parallels the destruction of increasingly large areas of forest in the Ecuadorian Amazon with the ensuing decline of biodiversity.

#### Variation.

Variation in dorsal and ventral coloration of preserved specimens is shown in Figure 19. Background dorsal coloration varies from grayish brown (e.g., QCAZ 48094) to pale grayish brown (e.g., QCAZ 48096) or pale brown (e.g., QCAZ 48094). Irregular dorsal marks may be present in diverse patterns. A faint middorsal line extends from the tip of snout to mid-dorsum (e.g., QCAZ 40079), mid-sacrum (e.g., QCAZ 40060) or to the cloaca (e.g., QCAZ 48095–96). There is variation in the number, size and shape of the dorsum marks. Five to seven broad transversal bands (sometimes interconnected) may be present on the dorsum; the dorsal surfaces of the limbs have brown transversal bars (one or two each on upper arm and forearm and three to five each on thigh, shank, and foot) (e.g., QCAZ 40080). Some individuals have scattered black or white dots on the dorsum (e.g., QCAZ 40060). The coloration of flanks varies from creamy white to gray with conspicuous dark brown irregular spots distributed from the groin to mid-flank. In some individuals, similar spots can also be present on the hidden surfaces of shanks and dorsal surfaces of feet (e.g., QCAZ 48096). The coloration of the hidden surfaces of thighs varies from creamy white to brown, with dark brown spots (e.g., QCAZ 40060) or blotches (QCAZ 48096).

Ventral areas of preserved specimens vary from creamy white (e.g., QCAZ 48094, 48096) to yellowish white (e.g., QCAZ 40079) with scattered flecks on the head and chest. In some individuals, the flecks are also present on hindlimbs, forelimbs, and belly (e.g., QCAZ 48095). Some individuals (e.g., QCAZ 48096) have a narrow to wide brown stripe on the outer edge of the hands, forearms, thighs and tarsal folds. Coloration of webbing and discs vary from brown to gray. Vomerine odontophores are triangular (with arched base in some specimens). Bones white.

In the adult type series, the largest male has a SVL of 32.24 mm, and the largest female 45.85 mm; mean male SVL = 31.72 mm (*n* = 5; SD = 0.42), female SVL range is 45.33 to 45.85 mm (*n* = 2). Inter-population variation in size and other morphometric variables are shown in Tables 1 and 2.

#### Coloration in life.

(based on photographs; Fig. 8F). Dorsal surfaces vary from pale yellowish tan (e.g., QCAZ 40060) to reddish brown (e.g., QCAZ 40079), with a brown middorsal line (e.g., QCAZ 48096); narrow pale brown longitudinal lines (e.g., QCAZ 40060, 48094) may be present. Some individuals have pale brown transversal bars on the dorsal surfaces of hindlimbs (e.g., QCAZ 40080); scattered minute black dots can be present on the dorsal surfaces of limbs and dorsum (e.g., QCAZ 40060); flanks are white (light blue in large females) with dark brown irregular marks with rounded (e.g., QCAZ 40060) or elongated shapes (e.g., QCAZ 48096); hidden surfaces of thighs are white or gray with dark brown spots (e.g., QCAZ 40060); in some specimens, the hidden surfaces of the shanks and dorsal surfaces of feet also have dark brown irregular spots (e.g., QCAZ 48096); venter creamy (e.g., QCAZ 48096) or yellowish white (e.g., QCAZ 40080) with scattered brown flecks on the ventral surfaces of the head and chest (e.g., QCAZ 48094); ventral surfaces of hindlimbs and forelimbs creamy white (e.g., QCAZ 40060) or yellowish white (e.g., QCAZ 40061); discs and webbing pale yellow tan; iris bronze (e.g., QCAZ 48096) or cream with faint yellow coloration on its upper half (e.g., QCAZ 40060); bones are white (e.g., QCAZ 40080).

#### Calls.

We recorded the calls of four malesat Comunidad Santa Rosa (Provincia Napo) on 28 February 2009, in flooded areas of secondary forest. Call parameters are shown in Table 7. Two call types were recorded. Type one (Fig. 13E–F) was the most common and consisted of a beep-like note with a mean duration of 0.10 s (SD = 0.02), average rise time 0.03 s (SD = 0.02) and average dominant frequency 1938.47 Hz (SD = 26.24). Type two (Fig. 13G–H) consisted of a single pulsed note with a mean duration of 0.11 s (SD = 0.02), mean rise time 0.05 s (SD = 0.02), and mean dominant frequency 1829.12 Hz (SD = 12.61). Call type two was alternated between calls of type one. Only males QCAZ 40060, 40080–81 produced this type of call.

#### Distribution and ecology.

*Hypsiboas tetete* is distributed in the Ecuadorian (Provincia Napo) and Peruvian Amazon basin (Región Loreto) (Fig. 15). Known localities range in elevation from 180 m (San Jacinto) to 420 m (Jatun Sacha). It is likely to have a larger distribution. Unfortunately, the lack of distinctive morphological characters relative to *Hypsiboas alfaroi*, preclude the unequivocal identification of museum specimens not associated with advertisement calls or genetic data. All specimens from Comunidad Santa Rosa and Jatun Sacha were found in flooded areas, in secondary forest, roosting on vegetation, 50 to 80 cm above ground.

Vegetation type for the Ecuadorian localities is Amazonian Lowland Evergreen Forest characterized by high plant alpha-diversity and a canopy of 30 m with emergent trees that reach 40 m.

Vegetation type at the Peruvian locality is Napo Moist Forest.

#### Conservation status.

The distribution polygon has 2,950 km^2^ of which 106 (3.5%) have been degraded by human activities (estimated from Ministerio de Ambiente del Ecuador 2013). Because its known distribution range is small with less than five localities and habitat degradation is increasing, *Hypsiboas tetete* is assigned to the Endangered category under criteria B1ab(iii).

### Morphometric comparisons between species

The following comparisons pertain to male SVL. There are significant differences between most species pairs. *Hypsiboas alfaroi* and *Hypsiboas tetete* are smaller than *Hypsiboas almendarizae*, *Hypsiboas fasciatus*, *Hypsiboas calcaratus*, and *Hypsiboas maculateralis* (all *P* values for *t* tests < 0.001); *Hypsiboas alfaroi* is larger than *Hypsiboas tetete* (*t* = 2.74, df = 31, *P* = 0.01); *Hypsiboas fasciatus* issmaller than *Hypsiboas almendarizae* (*t* = -3.87, df = 40, *P* < 0.001) and *Hypsiboas calcaratus* (*t* = -3.02, df = 52, *P* = 0.004); while *Hypsiboas almendarizae* is larger than *Hypsiboas maculateralis* (*t* = 2.79, df = 43, *P* = 0.008).

Two components with eigenvalues > 1.0 were extracted from the PCA for 136 males. The two components accounted for 61.30% of the total variation. The highest loadings for PC I were tibia length, femur length, and foot length; for PC II it was tympanum diameter (Table 8). The morphometric space shows high overlap among species (Fig. 7B). The first PC shows a partial segregation between *Hypsiboas maculateralis* (shorts limbs) and *Hypsiboas calcaratus* (long limbs). Comparisons of PC I between males of the six species show significant differences between *Hypsiboas fasciatus* and *Hypsiboas almendarizae* relative to *Hypsiboas calcaratus* and *Hypsiboas alfaroi* (all *P* values for *t* tests < 0.02); *Hypsiboas calcaratus* is significantly different from *Hypsiboas maculateralis* (*t* = 4.56, df = 55, *P* < 0.001), *Hypsiboas alfaroi* (*t* = 6.29, df = 65, *P* < 0.001), and *Hypsiboas tetete* (*t* = 3.43, df = 20, *P* = 0.003); *Hypsiboas fasciatus* is different from *Hypsiboas maculateralis* (*t* = 2.14, df = 39, *P* = 0.038); sister species *Hypsiboas alfaroi* and *Hypsiboas tetete* are also significantly different along PC I (*t* = -3.35, df = 15, *P* = 0.004). Comparisons of PC II (mainly tympanum diameter) shows that *Hypsiboas tetete* differs from all the others species (all *P* values for *t* tests < 0.05); while *Hypsiboas fasciatus* and *Hypsiboas almendarizae* are different relative to *Hypsiboas calcaratus*, *Hypsiboas maculateralis*, and *Hypsiboas alfaroi* (all *P* values for *t* tests < 0.05).

**Table 8.** Character loadings, eigenvalues, and percentage of explained variance for Principal Components (PC) I–II. The analysis was based on six morphometric variables of adult *Hypsiboas alfaroi*, *Hypsiboas almendarizae*, *Hypsiboas calcaratus*, *Hypsiboas fasciatus*, *Hypsiboas maculateralis*, and *Hypsiboas tetete*. Bold figures indicate highest loadings.

Variable	PCA Males		PCA Females	
	PC I	PC II	PC I	PC II
Foot length	**0.75**	–0.35	0.61	–0.58
Head length	0.22	0.01	0.17	**0.87**
Head width	0.66	0.32	**0.70**	0.33
Tympanum diameter	0.30	**0.89**	0.39	0.01
Tibia length	**0.81**	–0.37	**0.68**	0.12
Femur length	**0.81**	0.10	**0.75**	–0.15
Eigenvalue	2.49	1.18	2.10	1.25
%	41.55	19.74	35.06	20.83

Two components with eigenvalues > 1.0 were extracted from the PCA for 34 females. The two PCs accounted for 55.89% of the total variation. Principal Component I (35.06% of the variance) is positively correlated with head width, tibia length, and femur length, while PC II (20.83% of the variance) is correlated with head length (Table 8). Overall, there is wide overlap in morphometric space among species. However, there is segregation between *Hypsiboas almendarizae* and *Hypsiboas calcaratus* along PC I and *Hypsiboas almendarizae* and *Hypsiboas maculateralis* along PC II (Fig. 7A). In the PCA for females, PC I (mainly head shape and tibia length) showed significant differences between *Hypsiboas maculateralis* and *Hypsiboas calcaratus* (*t* = 3.24, df = 8, *P* = 0.012) and *Hypsiboas alfaroi* (*t* = -2.39, df = 16, *P* = 0.029); PC II (foot length) shows significant differences between *Hypsiboas almendarizae* relative to *Hypsiboas fasciatus* (*t* = 2.66, df = 7, *P* = 0.032), *Hypsiboas calcaratus* (*t* = -2.62, df = 6, *P* = 0.039), *Hypsiboas maculateralis* (*t* = -4.39, df = 8, *P* = 0.002), and *Hypsiboas alfaroi* (*t* = -2.84, df = 14, *P* = 0.013).

### Call comparisons between species

**Figure 20.**
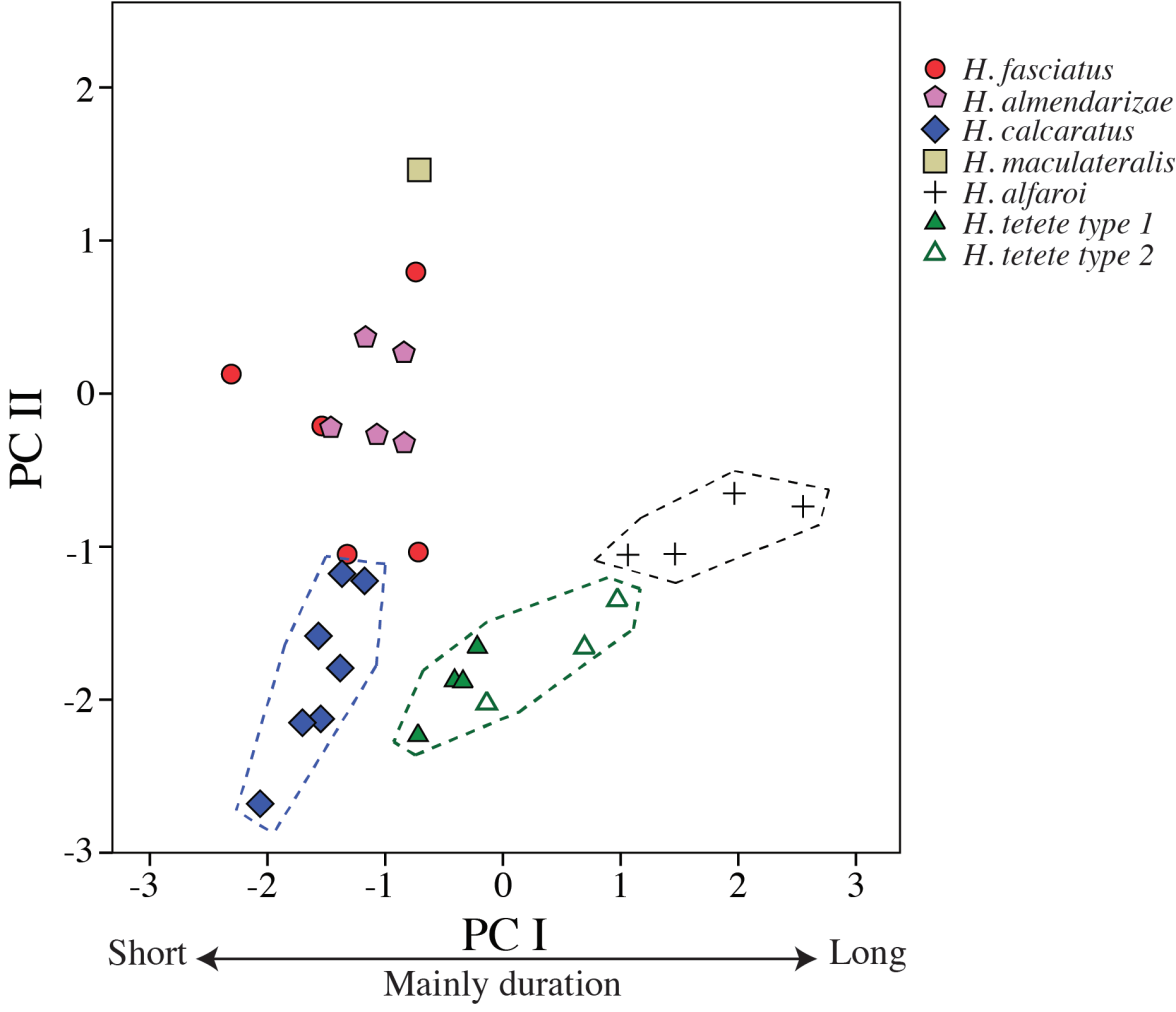
Axes I and II from Principal Components Analysis based on five acoustic variables from the advertisement calls of *Hypsiboas fasciatus* (**5** males), *Hypsiboas almendarizae* (**5**), *Hypsiboas calcaratus* (**7**), *Hypsiboas maculateralis* (**1**), *Hypsiboas alfaroi* (**4**), and *Hypsiboas tetete* (**4**). See Table 9 for character loadings on each component.

Two components with eigenvalues > 1.0 were extracted from the PCA of calls from 25 males. Both PCs accounted for 72.74% of the total variation. Principal Component I (46.27% of the variance) has high loadings on call duration and fundamental frequency; PC II (26.47% of the variance) has high loadings on number of notes and dominant frequency (Table 9). The acoustic space (as represented by PC I and PC II) shows segregation among the advertisement calls of all species except *Hypsiboas fasciatus* and *Hypsiboas almendarizae*, which are overlapping (Fig. 20). There are also qualitative differences among the advertisement calls. The closely related *Hypsiboas fasciatus*, *Hypsiboas almendarizae*, and *Hypsiboas calcaratus* share calls consisting of varying numbers of quack-like notes. These calls are markedly different from those of *Hypsiboas maculateralis*, *Hypsiboas alfaroi*, and *Hypsiboas tetete*. Although the calls of *Hypsiboas alfaroi* and *Hypsiboas tetete* appear close in acoustic space, they have a markedly different structure (Fig. 13C–H). Variation in recording temperature between species was low (< 6 C degrees; Table 7) and cannot explain the marked interspecific structural differences in advertisement calls.

**Table 9.** Character loadings, eigenvalues, and percentage of explained variance for Principal Components (PC) I–II. The analysis was based on five acoustic variables from the advertisement calls of *Hypsiboas alfaroi*, *Hypsiboas almendarizae*, *Hypsiboas calcaratus*, *Hypsiboas fasciatus*, *Hypsiboas maculateralis*, and *Hypsiboas tetete*. Bold figures indicate highest loadings.

Variable	Character Loading	
	PC I	PC II
Rise time	0.64	0.28
Call dominant frequency	0.35	**0.75**
Call duration	**0.95**	0.06
Number of notes	–0.42	**0.78**
Call fundamental frequency	0.82	–0.23
Eigenvalue	2.31	1.32
%	46.27	26.47

## Discussion

The use of genetic characters in the study of the systematics of Amazonian amphibians has resulted in the discovery of an unexpectedly large proportion of undescribed species. Most of those new species were previously considered populations of a single species with a large distribution range. Examples of these complexes include *Bolitoglossa peruviana*, *Engystomops petersi*, *Osteocephalus buckleyi*, *Osteocephalus taurinus*, *Pristimantis ockendeni*, *Rhinella margaritifera*, and *Scinax ruber* (Elmer et al. 2013; Elmer and Cannatella 2008; Fouquet et al. 2007; Funk et al. 2012; Jungfer et al. 2013; Ron et al. 2012). *Hypsiboas calcaratus* and *Hypsiboas fasciatus* show a similar pattern because both were considered to have a widespread distribution comprising most of the Amazon Basin (IUCN 2011). Our results and those from Funk et al. (2012) indicate that they form a complex of eleven candidate species. Available morphological and advertisement call data allowed us to confirm the species status of six of them. Two correspond to *Hypsiboas calcaratus* and *Hypsiboas fasciatus* and four are new species described herein. The five remaining candidate species remain unconfirmed although geographic distribution suggests that clade J from Buenavista, Bolivia (Fig. 1) could correspond to *Hyla steinbachi* (type locality Sara province, Departamento Santa Cruz, Bolivia). Ichilo province (where Buenavista is found, the source of genetic samples for this study) is adjacent to Sara province.

### Implications of the discovery of hidden species richness in our current understanding of the biology of Amazonian amphibians

The discovery of hidden species richness could require a review of our current understanding of the biogeography, evolution and conservation status of Amazonian amphibians (Icochea and Angulo 2010, Peloso 2010). This need arises from the anticipated increase in the total number of described species and the availability of more accurate assessments of the geographic distribution of the species. Although the number of genetic-based studies on the systematics of Amazonian amphibians is still limited, our results and those from other species complexes (see above) suggest that the changes could be substantial. In the *Hypsiboas calcaratus*-*Hypsiboas fasciatus* complex the number of species increased three to five times and there was a sizeable decrease in the distribution range of *Hypsiboas fasciatus*. According to the distribution polygon of the IUCN Red List (Icochea et al. 2004), *Hypsiboas fasciatus* has a distribution of 5’867,000 km^2^. Our results show that the distribution polygon of *Hypsiboas fasciatus*, based on the localities shown in Fig. 15, does not overlap with the Red List polygon and has less than 10,000 km^2^, about 0.014% of Red List estimate. If these changes are typical among Amazonian amphibians, the reliability of large global databases, like those of the IUCN Red List, Global Amphibian Assessment (http://www.iucnredlist.org/initiatives/amphibians) could be compromised. The same problem would affect large scale analyses of biogeography, evolution, and conservation of amphibians that have relied those databases (e.g., Buckley and Jetz 2007; Pyron and Wiens 2013).

The conservation status of Amazonian amphibians will also require a reassessment under the new taxonomy. The aggregated results for the *Hypsiboas fasciatus* species complex show a decrease in the proportion of Least Concern species from 100% to 50%. If this amount of change is representative of Amazonian amphibians, we would expect an increase in the proportion of species under threatened categories.

The use of molecular tools is revolutionizing and reinvigorating the fields of taxonomy and systematics. By combining genetic data with other sets of independent characters, a practice that has been recently referred as “integrative taxonomy” (Dayrat 2005; Padial and De la Riva 2009), species boundaries can be defined objectively. These new tools have allowed documenting large numbers of undescribed Amazonian amphibians, which highlight the need for dense scientific collections (including genome samples) and comprehensive molecular-based taxonomic reviews. The completion of the inventory of species should be considered a priority because other scientific endeavors cannot succeed without reliable taxonomic data.

## Supplementary Material

XML Treatment for Hypsiboas calcaratus

XML Treatment for Hypsiboas fasciatus

XML Treatment for Hypsiboas almendarizae

XML Treatment for Hypsiboas maculateralis

XML Treatment for Hypsiboas alfaroi

XML Treatment for Hypsiboas tetete

## Appendix

### Examined specimens

*Hyla leptoscelis*. BRAZIL: AMAZONAS: Lago do Tachy, above São Paulo Olivença, Rio Solimöes (BMNH 1947.2.23.10, holotype).

*Hyla steinbachi*. BOLIVIA: PROVINCIA SARA: Departamento Santa Cruz de la Sierra (BMNH 1947.2.13.61–63, syntypes).

*Hypsiboas calcaratus*. ECUADOR: PROVINCIA ORELLANA: Río Napo, San Vicente (0.6790°S, 75.6511°W), 203 m (QCAZ 44529–30); Río Napo, Chiroisla (0.5756°S, 75.8998°W), 203 m (QCAZ 44422); Río Napo, Añangu (0.5249°S, 76.3844°W), 255 m (QCAZ 43933–36, 43979); Río Napo, Edén (0.4983°S, 76.0711°W), 216 m (QCAZ 44176–79, 44225, 44246–47, 44286); Estación Científica Tiputini, Universidad San Francisco de Quito (0.6387°S, 76.1492°W), 230 m (QCAZ 12343–44); Río Rumiyacu (0.89566°S, 75.94783°W), 250 m (QCAZ 20547–51); Parque Nacional Yasuní, between km 80 and km 75, on the road Pompeya-Iro (0.8401°S, 76.3024°W), 243 m (QCAZ 43057, 43060, 43062–63); Pompeya-Iro road, 38.8 km from Pompeya (0.6535°S, 76.4535°W), 237 m (QCAZ 8201); Parque Nacional Yasuní, km 9 Pompeya (0.4598°S, 76.5931°W), 253–271 m (QCAZ 43034–35, 43038, 43046–47); Estación Científica Yasuní, Universidad Católica del Ecuador (0.6713°S, 76.4005°W), 230–250 m (QCAZ 8210, 8812, 11912–13, 12386, 14815–17, 16792–97, 16798–99, 17825, 18264, 19202, 19204–05, 20290, 20305, 20837, 22488–89, 22561–66, 22866, 22988, 23038–39, 23065–69, 23848–54, 24214, 24282, 31445–46, 36869–78, 40985–86, 41005–06, 41015, 43242–48, 43256–59, 49205); Río Napo, sector La Primavera (0.4442°S, 76.7868°W), 244 m (QCAZ 43824); El Coca (0.4778°S, 76.9898°W), 257 m (QCAZ 43713, 43789); La Belleza, Comunidad Bocana del Suno (0.6922°S, 77.1353°W), 309 m (QCAZ 33522, 33524); Aguarico, confluence of the Yasuní and Jatuncocha rivers (0.9836°S, 75.4183°W), 200 m (EPN 5085–86); Aguarico, Ishpingo II oil well (1.0947°S, 75.6494°W), 178 m (EPN 3865); Loreto, San José Daguano (0.8255°S, 77.4347°W), 450 m (EPN 5674); San Luis de Armenia (0.4822°S, 77.0683°W), 300 m (EPN 11739); PROVINCIA SUCUMBÍOS: Cuyabeno, Caña de Canangüeno (0.3990°S, 75.8753°W), 222 m (QCAZ 11924); Reserva Faunística Cuyabeno (0.08498°N, 76.13444°W), 273 m (QCAZ 2034, 2046–49, 2242, 4612); Laguna de Mateococha (0.01846°N, 76.22155°W), 220 m (QCAZ 26062); Puerto Bolívar (0.0886°S, 76.1420°W), 240 m (QCAZ 28181, 28185, 28197); La Selva Lodge (0.4981°S, 76.3738°W), 245 m (QCAZ 4333, 25419, 25434); La Selva Lodge, Mandi Cocha (0.41666°S, 76.1333°W), 250 m (QCAZ 11540–41, 11545, 12005–07, 12009); Reserva Limoncocha (0.4062°S, 76.6194°W), 261 m (QCAZ 43100, 43131,43268); Shushufindi (0.0331°S, 76.6535°W), 270 m (QCAZ 15188); Zancudococha (QCAZ 4535); Tarapoa (QCAZ 23095); Cofán Duvuno community (0.0333°S, 77.1166°W), 340 m (EPN 4917); PROVINCIA NAPO: Reserva Yachana (0.8333°S, 77.1666°W), 300 m (QCAZ 48833); Comunidad Santa Rosa, on the road to Tena (0.9895°S, 77.4412°W), 341–439 m (QCAZ 40055–56, 40083); Tena, on the road to Jatun Sacha (1.0449°S, 77.76951°W), 428 m (QCAZ 18173, 40084–85); on the road Hollín-Loreto (QCAZ 649); La Selva (QCAZ 7436–40, 7443); PROVINCIA PASTAZA: Canelos Sacha (1.5822°S, 77.7155°W), 500–650 m (QCAZ 14956–75, 48712, 48718–20); Bataburo Lodge (1.2083°S, 76.7166°W), 220 m (QCAZ 39442); Kapawi Lodge (2.5386°S, 76.8583°W), 239 m (QCAZ 9018, 25513–14); Misión (2.2143°S, 76.5142°W), 240 m (EPN 885); Montalvo (2.0666°S, 76.9666°W), 305 m (EPN 884); Shionayacu (2.1001°S, 76.6334°W), 360 m (EPN 886); FRENCH GUIANA: Kaw (4.7166°N, 52.1333°W), 3 m (105PG); Cayenne, Crique Arataye (4°N, 52.66°W), 70 m (USNM 247780); Lac Toponow (3.0527°N, 52.7102°W), 152 m (123PG); Trinite (4.5833°N, 53.35°W), 290 m (192BM); Crique Margot (5.4666°N, 53.95°W), 44 m (131MC); GUYANA: Rupununi, Iwokrama Forest Reserve, 5 hrs S (downstream) of Kurupukari (4.285°N, 58.50944°W), 99 m (USNM 531370, 531372); BRAZIL: RONDONIA: Nova Brasilia (11.15°S, 61.566 °W), 431 m (USNM 304048); PERU: REGIÓN MADRE DE DIOS: Cuzco Amazónoico, 15 km E Puerto Maldonado (12.583°S, 69.083°W), 200 m (MUSM 14456, 14547, KU 215205); Río Tambopata, W bank of Zona Reservada Tambopata-Candamo (13.1416°S, 69.6066°W), 211 m (USNM 332415); Pakiza, Manu National Park (11.866°S, 71.3°W), 360 m (USNM 345216–17); REGIÓN LORETO: Río Lagarto Cocha, Aguas Negras (0.5230°S, 75.2569°W), 183 m (USNM 520924).

*Hypsiboas fasciatus*. ECUADOR: Andes of Ecuador (BMNH 58.4.25.22; holotype); PROVINCIA MORONA SANTIAGO: 7.9 km N Limón, on the road Limón-Macas (2.8858°S, 78.3970°W), 1571 m (QCAZ 26496–97); Comunidad Nueva Principal, 3 km near to the town (3.1686°S, 78.3870°W), 1059 m (QCAZ 48611, 48628); Tiink, Río Zamora (3.3330°S, 78.4518°W), 730 m (QCAZ 17016, 17123, 17128, 17030–31, 17040–41, 17148); Comunidad San Luis (3.3420°S, 78.4677°W), 792 m (QCAZ 48633, 48636, 48639, 48670–71); La Pradera, on the road to Gualaquiza (3.3365°S, 78.6504°W), 1015–1036 m (QCAZ 18270–71, 48584–93); road to Río Abanico Jimbitano, 1000 m (QCAZ 21188–89); road between Gualaquiza and Limón, 3 km from Limón (3.3990°S, 78.5786°W), 1140 m (QCAZ 27610); San Juan Bosco, Cóndor Mirador (3.5192°S, 78.4314°W), 1100 m (EPN 14060–65); 3 km Gualaquiza-Limón (3.3700°S, 78.5680°W), 1140 m (QCAZ 21707, 21957, 33976); PROVINCIA ZAMORA-CHINCHIPE: Gravel road East to Sarsa (3.8078°S, 78.6059°W), 1500 (QCAZ 47051); Piuntza, Finca Don Mesías (3.8564°S, 78.8646°W), 1154–1192 m (QCAZ 40797, 40802); near to Zamora (4.0491°S, 78.9256°W), 927 m (QCAZ 27258); Zamora (4.0669°S, 78.9508°W), 1060 m (QCAZ 23144, 23147–49, 48583); Bombuscaro (4.0893°S, 78.9609°W), 1091 m (QCAZ 12444, 12446, 12448, 12452, 12494–97); La Pituca (4.1461°S, 78.9446°W), 1343 m (QCAZ 24650); Romerillos Alto, 26 km of Zamora (4.1850°S, 78.9352°W), 1300–1593 m (QCAZ 24866, 47070); Las Orquidias, around the town (4.2290°S, 78.6577°W), 874 m (QCAZ 41488); Miazi Alto, “Tepuy” camp (4.2562°S, 78.6222°W), 1250–1300 m (QCAZ 41575–76, 41659, 41518); Destacamento Militar Miasi (4.2833°S, 78.6333°W), 800 m (EPN 4112–13, 4115–16, 4118); Paquisha, Blanco river (3.8945°S, 78.5161°W), 1630 m (EPN 14218); Yantzaza, Colibrí Goldmarca (3.7665°S, 78.5055°W), 1377 m (EPN 12672, 12674, 13288, 13290–92); Yantzaza, Concesión Colibrí (3.7588°S, 78.5009°W), 1424 m (EPN 11387–91, 12316); Yantzaza, Sachavaca sector (3.7042°S, 78.4827°W), 1280 m (EPN 14216–17); Yantzaza, road to Pindal (3.7522°S, 78.5528°W), 1224 m (EPN 13677); Centro Shuar Yawi (4.4301°S, 78.6489°W), 945 m (QCAZ 31037–40); near to Tundaime (QCAZ 16466); PERU: REGIÓN AMAZONAS: Cordillera del Cóndor, Upper Río Comainas, Puesto Vigilancia (4.1°S, 78.3833°W), 1100 m (USNM 525495, 525499).
